# The financial consequences of undiagnosed memory disorders

**DOI:** 10.1016/j.jfineco.2025.104149

**Published:** 2025-08-19

**Authors:** Carole Roan Gresenz, Jean M. Mitchell, Belicia Rodriguez, Crystal Wang, R. Scott Turner, Wilbert van der Klaauw

**Affiliations:** a McCourt School of Public Policy and School of Health, Georgetown University, 125 E St NW, Washington DC, 20001, USA; b McCourt School of Public Policy, Georgetown University, 125 E St NW, Washington DC, 20001, USA; c Federal Reserve Bank of New York, 33 Liberty St, 10045, NY, USA; d Department of Neurology, Georgetown University, 4000 Reservoir Rd NW, Washington DC, 20057, USA

**Keywords:** Household finance, Credit behavior, Cognitive function, Alzheimer’s disease and related dementia

## Abstract

We examine the effect of undiagnosed memory disorders on credit outcomes using individually-matched nationally representative credit reporting and Medicare data. We find effects of early stage disease, years before diagnosis, on a wide range of financial outcomes, including credit card account payment delinquency and amount of delinquent balance, credit utilization among credit card account holders, mortgage delinquency and delinquent balance amount, and credit scores. Effects are pervasive, affecting seniors in single and coupled households, racial/ethnic minorities and non-minorities, and older adults living in areas with higher and lower education levels. Early stage effects are greater among singles and Black individuals.

For households with older adults, financial decisions are imbued with particular consequence, given increasing reliance with age on largely fixed assets (versus new income flows) and shifting resource demands for health-related needs (Jacobson et al., 2021; Machlin and Carper, 2018). Among recent cohorts of older-adult households, declining defined retirement benefits, lengthening average periods of retirement, and increasing expected health and long-term care costs (Agarwal et al., 2009) have added to the consequential nature of financial decision-making.

The range and complexity of financial decisions in which households engage underscore the role of cognitive function in financial behavior. These include balancing consumption and savings over an uncertain time horizon, optimizing performance while mitigating risk in an asset investment strategy, and determining whether to initiate debt and the structure of such debt (Gomes et al., 2021). Previous studies find cognitive ability is related to financial decisions regarding assets, including wealth and wealth composition (McArdle et al., 2011); investment performance (Kuo et al., 2014); trading behavior and performance (Grinblatt et al., 2012); and stockholding (Christelis et al., 2010). With respect to liability, cognitive function has been linked to optimal credit card use (Agarwal and Mazumder, 2013), the probability of mortgage default (Gerardi et al., 2013), and repayment behavior (Brown et al., 2016).

Alzheimer’s disease and related dementias (ADRD) present a threat to cognitive function among older adults, and, consequently, may compromise financial decision making. These memory disorders affect a substantial share (more than 11 percent) of the older adult population (Alzheimer’s Association, 2023). In addition to affecting cognitive function, ADRD is also associated with personality changes and neuropsychiatric symptoms (Eikelboom et al., 2021; Robins Wahlin and Byrne, 2011) that may influence financial behavior (Parise and Peijnenburg, 2019; Costello et al., 2019; Peterson, 2007; Kuchler and Stroebel, 2021). ADRD is progressive, with increasing symptoms over time (Jack et al., 2018). This suggests the possibility of greater effects on financial behavior in the disease’s later stages when symptoms are more severe; however, adverse consequences once diagnosis is established may be partially offset by additional oversight that family members and loved ones provide. By contrast, the mild to moderate symptoms characterizing the early stages of disease may be difficult for others to recognize. As a result, individuals may experience heightened financial vulnerability during the years between the onset of first symptoms and ADRD diagnosis.

This study examines how ADRD, in its early stage prior to diagnosis, affects financial outcomes. We begin with the creation of a unique, massive data set with nationally representative, longitudinal credit data spanning nearly two decades (Lee and van der Klaauw, 2010) that is matched at the individual level with Medicare claims and enrollment data using a unique identifier (Social Security number). Our matching approach results in a high quality 91.8 percent match rate, and our analytic sample includes nearly a half million individuals ever diagnosed with ADRD, approximately 2 million individuals never diagnosed with ADRD, and a total of more than 137 million quarterly observations. The large sample size ensures ample statistical power for detecting potentially small effects of ADRD many years prior to diagnosis and supporting sub-population analyses, such as by household composition, race, and education level.

Methodologically, we implement an event study model with individual and time fixed effects, with varying event dates and inclusion of never-treated individuals (Miller, 2023). Identification of early stage disease effects on financial outcomes relies on variation in the timing of diagnosis and within-individual changes over time in outcomes, as well as a comparison group. The inclusion of individual fixed effects allows us to account for observed and unobserved non time-varying personal characteristics that are correlated with the probability of developing ADRD and financial outcomes, and we control for a robust set of time-varying characteristics, including household structure, health status, and state-by-quarter covariates that account for local macroeconomic effects on financial outcomes. We adjust for observed differences in measured covariates by applying propensity score weighting (Abadie, 2005; Hirano et al., 2003) to address concerns about the comparability of those who are ever or never diagnosed with ADRD.

Additional analyses further buttress interpretation of our results as causal estimates. First, we institute a test for differences in financial outcomes between those ever and never diagnosed with ADRD in the “pre” period before the likely onset of early symptoms. Findings from these analyses lend further support to the comparability of the groups we compare. Second, we show that our results are robust in unweighted analyses and analyses that drop the comparison group of individuals never diagnosed with ADRD.

Third, we conduct placebo treatment tests (Eggers et al., 2024) in which independent variables measuring time to ADRD diagnosis are replaced with indicators of time to diagnosis for other conditions. The results support the validity of the underlying assumption that variables measuring the time to diagnosis of ADRD are capturing symptoms associated with the disease as opposed to other unobserved factors that are associated with time to diagnosis of health conditions more generally.

Our research investigates a range of outcomes to provide insight into the pathways through which ADRD affects finances prior to diagnosis. We examine credit score, which is an overall measure of an individual’s credit worthiness, as well as any payment delinquency across the full range of an individual’s credit accounts. We also investigate payment delinquency and delinquent balances among credit card and mortgage accounts and credit utilization among credit card account holders. Payments on installment accounts such as mortgages are typically fixed amounts paid on a regularly scheduled basis, whereas payments for revolving accounts such as credit cards vary with spending against an ongoing line of credit. Both may be affected by early stage ADRD, with the latter more likely to be sensitive to day-to-day spending and financial management.

We additionally investigate heterogeneity in the effects of early stage ADRD on outcomes by household composition (single vs coupled), race/ethnicity, and education. Previous research points to the potential for adverse financial effects of cognitive impairment on individuals in coupled households (Gresenz et al., 2019; Angrisani and Lee, 2019). Nearly half of seniors live in coupled households (Ausubel, 2020), making the effects on these individuals important to understand. With respect to race, although access to Medicare at age 65 reduces racial and ethnic disparities in health and access to care, it does not eliminate them (Wallace et al., 2021), and previous studies document delayed diagnosis among Black and Hispanic individuals (Lin et al., 2021; Chen et al., 2024). A delayed diagnosis is likely to affect the average symptoms an individual is experiencing at given points in time relative to diagnosis. We account for this with race/ethnicity-stratified analyses. We also conduct analyses stratified by education measured at the Census tract level. Average symptoms relative to diagnosis are likely to be more pronounced among individuals with lower educational attainment, as higher levels of education have been shown to offer some protection against cognitive decline (Amieva et al., 2005).

Our findings establish a broad foundation for understanding the effects of ADRD, before it is diagnosed, on financial outcomes. First, we show that the effects of early stage ADRD on credit scores are consistent and progressive over time, with small but detectable effects many years before diagnosis and effects that are larger in magnitude in quarters closer to diagnosis. The same holds for payment delinquency. We document that early stage ADRD affects credit scores in every quarter during the 6.5 years prior to diagnosis and the probability of delinquency in every quarter during the 7 years prior to diagnosis. The increases in payment delinquency and declines in credit score are large. One year prior to diagnosis, the probability of delinquency is 19.5 percent (1.5 percentage points) higher than baseline, for example, and credit scores are on average 5.3 to 7.3 points lower during the year just preceding ADRD diagnosis.

Second, our findings highlight the range of the impact of early stage ADRD on financial outcomes. The disease affects the management of predictable repayment obligations, such as mortgages, as well as accounts with more repayment variability, such as credit cards. For the latter, effects occur earlier in the disease life cycle and affect the probability of delinquency, delinquent balances, the credit utilization rate, and the probability of being “maxed-out” on credit card accounts. These findings point to the likely channels through which effects operate, including forgotten payments, individuals’ ability to navigate interruptions to automatic bill payment systems, account management, and spending behavior. Later in the disease life cycle, as many as three years prior to diagnosis, we find effects of ADRD on mortgages. Beyond forgotten payments, these effects may occur when individuals with early stage ADRD have become financially overextended and unable to meet their scheduled repayment obligations.

Third, our findings show that the effects of early stage ADRD on financial outcomes are pervasive, affecting seniors in both single and coupled households, individuals who are racial/ethnic minorities and non-minorities, and those living in areas with higher and lower education levels. Individuals in coupled households enjoy some, but not complete protection against the effects of ADRD prior to diagnosis. This likely reflects benefits from shared household financial decision making (Fonseca et al., 2012). We find more pronounced adverse financial outcomes among Black compared to White adults with early stage ADRD. In addition to a more fragile financial standing and a greater vulnerability to cognitive decline, the differences we observe derive in part from delays in diagnosis and differences in the speed of disease progression. The timing and magnitude of effects of ADRD are similar prior to diagnosis among individuals living in areas with lower or higher levels of education. Following diagnosis, effects of ADRD are greater among individuals in lower education areas.

Although ours is not the first study to examine this research question, our unique data allow us to explore this question more comprehensively and definitively than has been previously possible, and we address key weaknesses in previous studies. Gresenz et al. (2019), for example, examine the effect of early stage ADRD on liquid assets and net wealth using time to diagnosis as a composite measure of symptoms associated with ADRD. The study relies on financial outcomes from the Health and Retirement Study (HRS) survey data merged with information on the timing of ADRD diagnosis from Medicare claims data, and finds that early stage ADRD places households at significant risk for large adverse changes in liquid assets, regardless of who in the household is affected, and reduces net wealth, with a more pronounced effect when the financial head of household is affected. However, financial outcomes are self-reported, measured only every other year, and limited in terms of their specificity.

Nicholas et al. (2021) analyze the effects of time to ADRD diagnosis on two outcomes (the probability of payment delinquency and having a subprime credit score) among seniors living alone using merged credit and Medicare data. For the same two outcomes using a comparable sample of single households, we find effect sizes that are two to three times larger prior to diagnosis and more than four times as large at diagnosis. Our data are matched using a unique identifier (SSN) whereas previous estimates are based on data merged using a set of household characteristics. Deterministic matching on non-unique identifiers results in measurement error and estimation bias, often requires arbitrary decisions of researchers, and cannot quantify match uncertainty (Enamorado et al., 2019). These issues are amplified when the datasets being merged are large (Enamorado et al., 2019). Previous findings are not generalizable to the majority of seniors because only 27 percent of adults ages 60 and older live alone (Ausubel, 2020), whereas we study all households and document statistically significant effects of ADRD among coupled households. Additionally, the comparatively small sample previously used (81,364 in total vs. our sample of 2.4 million) affects the precision of estimates, particularly for subgroup analyses. We find consistent patterns of non-zero effects of ADRD prior to diagnosis among individuals in low and high education areas. We also find similarity in the timing of effects by education. By contrast, Nicholas et al. (2021) find statistically significant effects of ADRD among those in higher education areas in only a few time periods close (within 1–2.5 years) to diagnosis. In addition, our data permit analysis of variability in the effects of ADRD by race/ethnicity, which was not previously studied.

Our findings are important because the adverse effects of undiagnosed ADRD on credit scores, credit card delinquency, mortgage delinquency, high credit utilization, and delinquency on other accounts increase immediate pecuniary costs such as late fees and interest charges. These changes also reduce access to credit and affect credit limits and interest rates on credit cards and personal loans, all at a time when the demand for household financial resources is likely to increase to pay for the substantial caregiving and related costs associated with the later stages of memory disorders (Hurd et al., 2013). Our findings highlight the need to understand whether recent legal and regulatory changes have helped mitigate adverse outcomes among those with yet-to-be diagnosed memory disorders, who are susceptible both to compromised decision-making on their own and to financial abuse and exploitation from others (Carlin et al., 2023; DeLiema et al., 2020), and to consider additional opportunities for financial institutions and government agencies to reduce or prevent poor financial outcomes. Our findings also highlight the importance of tools to facilitate early diagnosis of ADRD. Previous work has shown that money management difficulty can help identify vulnerable individuals with early stage dementia (Agarwal and Muckley, 2024). Our findings lend further support to the possibility that information on changes in financial behavior could be effectively used to facilitate early identification of those at risk for ADRD.

## 1. Background on Alzheimer’s disease and data

### 1.1. Alzheimer’s disease and related dementia

Older households are those most vulnerable to memory disorders such as Alzheimer’s disease and related dementias (ADRD) which impair cognitive function. Alzheimer’s disease (AD) is the most common type of dementia and in more than 90 percent of Alzheimer’s disease cases, symptoms first occur after age 65 (National Institute on Aging (NIA), 2019). ADRD is a broader construct which includes AD as well as other types of dementia that are clinically similar.^3^ The stages of ADRD include the preclinical stage, when symptoms are not present but changes may be occurring in the brain; early stage, when symptoms are mild; middle stage, when symptoms are moderate; and late stage when symptoms are severe (National Institute on Aging (NIA), 2021). Previous research profiles declining cognitive function during the 9 years prior to Alzheimer’s diagnosis using measures of general cognitive function, visual memory, verbal fluency, and abstract thinking (Amieva et al., 2005). Other research documents 12 year patterns in executive function, semantic memory, verbal and visual episodic memory and finds evidence of decline starting more than 10 years prior to diagnosis of AD (Mistridis et al., 2015). Amieva et al. (2005) also document a steeper gradient in cognitive decline among individuals with less education.

ADRD has a substantial and growing reach, affecting more than 11 percent of individuals age 65 and over in the U.S., and the size of the population affected is projected to more than double by 2050 as a result of epidemiologic and demographic trends (Alzheimer’s Association, 2023). Certain sub-populations are particularly at risk. For women, the estimated lifetime risk for Alzheimer’s disease at age 45 is double that for men (Chêne et al., 2015), and the prevalence of ADRD is decidedly more pronounced among Black and Hispanic populations (Chen and Zissimopoulos, 2018; Lines et al., 2014) and lower for Asian American populations compared to non-minority populations (Mayeda et al., 2017). In addition, research has identified fourteen potentially modifiable risk factors that are associated with ADRD: less education, hypertension, hearing impairment, vision impairment, high LDL cholesterol, smoking, obesity, depression, physical inactivity, diabetes, low social contact, excessive alcohol consumption, traumatic brain injury, and air pollution (Livingston et al., 2024).

### 1.2. ADRD and financial behavior

Studies have identified specific cognitive domains that are affected by ADRD during the disease lifecycle, including semantic memory (storing and processing general knowledge), episodic memory (the ability to recall specific events) and executive function (skills for planning, problem solving and emotional management) in earlier stages of disease and working memory processing speed (how quickly the brain understands information) and global cognition in later stages (Jutten et al., 2021). In addition, ADRD can affect a range of neuropsychiatric dimensions such as disinhibition, irritability, apathy, and agitation (Eikelboom et al., 2021), and impact personality traits, including reducing conscientiousness, increasing neuroticism, and reducing extraversion (Robins Wahlin and Byrne, 2011). Like cognitive function, neuropsychiatric symptoms and personality traits have been linked to financial behavior. Parise and Peijnenburg (2019) find individuals with lower levels of conscientiousness and emotional stability have a higher likelihood of experiencing financial distress; Costello et al. (2019) show an association between disinhibition and maladaptive financial behaviors; and Peterson (2007) reviews evidence on the influences of moods, attitudes, and emotions on financial decision-making. The diminished extraversion and increased apathy characteristics of ADRD may also affect social interactions, which are important in shaping financial decisions (Kuchler and Stroebel, 2021). Previous studies suggest financial decision-making deficits are often among the first functional changes attributable to early stage ADRD and that the effects of ADRD on financial behavior are magnified as symptoms progress (Marson et al., 2000; Martin et al., 2008; Triebel et al., 2009).

These changes to financial behavior can result in financial vulnerability among those in early stage disease through two pathways. First, early stage disease ADRD may make individuals more susceptible to compromised financial decision-making on their own (Han et al., 2015b), and second, it may render individuals more susceptible to financial exploitation by others (Han et al., 2015a; Wood and Lichtenberg, 2017). The potential financial vulnerability created by early stage ADRD is compounded by the challenges of detecting and diagnosing mild cognitive impairment (Sabbagh et al., 2020) and the years-long time span of the prodromal stage, when mild symptoms may be manifesting but diagnosis may not yet be possible (Vermunt et al., 2019). Relatedly, Mazzonna and Peracchi (Mazzonna and Peracchi, 2024) show that older individuals often underestimate their own cognitive decline and Ameriks et al. (2023) point to the difficulty of optimizing when to delegate control over finances.

### 1.3. Data sources

We use data from the Federal Reserve Bank of New York’s (FRBNY) Consumer Credit Panel (CCP) spanning 2000–2017 merged at the individual level using a unique common identifier (Social Security number) with data from the Base, Cost & Use, and Chronic Conditions segments of the Medicare Beneficiary Summary File (Centers for Medicare & Medicaid Services, 2023a) covering the same time period. We also merge information from the American Community Survey (U.S. Census Bureau, 2010–2017) and County Health Rankings & Roadmaps (2024) on characteristics of the local areas in which individuals reside.

#### 1.3.1. Credit report data

The CCP was designed to provide an anonymized, nationally representative sample of U.S. residents with a credit history and an SSN associated with their credit file (Lee and van der Klaauw, 2010). The CCP derives from credit data collected and stored by Equifax, one of three primary credit bureaus to collect such information in the U.S. These agencies compile and maintain credit histories for all U.S. residents who have applied for or opened a loan account—an estimated 89 percent of all U.S. adults (Brevoort et al., 2016; Lee and van der Klaauw, 2010). The credit data are highly granular, including an overall derived credit score as well as detailed information on loan origination, amount, balance, term, credit limit and payment status/delinquencies for, separately, mortgages, home equity loans and revolving accounts, auto loans, credit card accounts, consumer finance and retail loans, and other loan accounts. These variables are updated continuously, and, for most adults, over the time span from early adulthood forward. Avery et al. (2003) provide a comprehensive overview and additional detail on consumer credit reports content. A key advantage of the CCP for this research is that it tracks financial outcomes from administrative as opposed to self-reported data. Self-reported borrowing has been shown to lead to underestimation of some debt amounts (Brown et al., 2015). The administrative nature of the data is especially important because the primary population of interest in the research includes individuals with increasing cognitive impairments whose ability to self-report outcomes may be compromised.

To create the CCP, a first stage random sample of 5 percent of all individuals with a credit history and SSN was selected, based on the last four (random) digits of SSN. For each primary sample member in the CCP, information is then collected on other individuals living at the exact same address (secondary sample members). The first stage 5 percent sample includes approximately 12 million individuals. The total sample, after second stage sampling, includes approximately 38.1 million individuals representing 11.2 million households. The sampling scheme was repeated for every quarter beginning with quarter 1 of 1999 through the present day in order to create a longitudinal panel. New individuals are added (e.g., adults who establish a credit history for the first time) and others are dropped (e.g., deaths or emigrations) in each quarter so that the sample is representative of the target population in each quarter, while at the same time comprising a representative panel over time. Our data comprise 72 quarters (18 years) of CCP panel data (2000–2017). More details regarding the CCP sample design can be found in Lee and van der Klaauw (2010).

#### 1.3.2. Medicare claims and enrollment data

We use multiple segments of the Master Beneficiary Summary File (MBSF) from 2000–2017, which contain data on all Medicare enrollees for a given calendar year (Centers for Medicare & Medicaid Services, 2023b). The MBSF Base Segment contains socio-demographic and enrollment data for all enrollees, including state/county/zip code of residence; date of birth; date of death (if applicable); gender; race/ethnicity; dual Medicaid-Medicare enrollment status; and plan enrollment type (traditional Medicare vs. Medicare Advantage). The MBSF Chronic Conditions and Cost & Use segments are based on claims data and are for traditional Medicare enrollees only, as health care utilization among managed care (known as Medicare Advantage) enrollees does not generate claims. The MBSF Chronic Conditions segment, known as the Chronic Conditions Warehouse, includes indicators of the presence or absence of various clinical conditions and the date of diagnosis of each. The MBSF Cost & Use segment contains a measure of out-of-pocket health care spending.

Clinical indicators in the Chronic Conditions Warehouse are based on algorithms CMS has established for each chronic condition (Chronic Conditions Data Warehouse, 2023). Conditions identified in the Chronic Conditions segment include Alzheimer’s Disease, ADRD and 25 other chronic conditions.^4^ For each condition, the date of first occurrence is measured, and flags earmark mid- and end-of year presence of the condition.^5^ Previous research has employed a claims-based approach to identifying ADRD (Mucha et al., 2008; Joyce et al., 2007; Gresenz et al., 2019) and examined the sensitivity and specificity of claims data for identifying ADRD (Taylor et al., 2009; Grodstein et al., 2022). Sensitivity represents the probability that a person who has ADRD is identified as having ADRD from claims data whereas specificity represents the probability that a person who does not have ADRD is accurately identified as cognitively healthy. Thus, a high sensitivity rate indicates few false negatives and a high specificity rate indicates few false positives. Taylor et al. (2009) report sensitivity and specificity of 0.85 and 0.89, respectively, for identifying ADRD using claims data and Grodstein et al. (2022) report sensitivity and specificity of 0.79 and 0.88.

### 1.4. Data construction

**Fig. 1.**
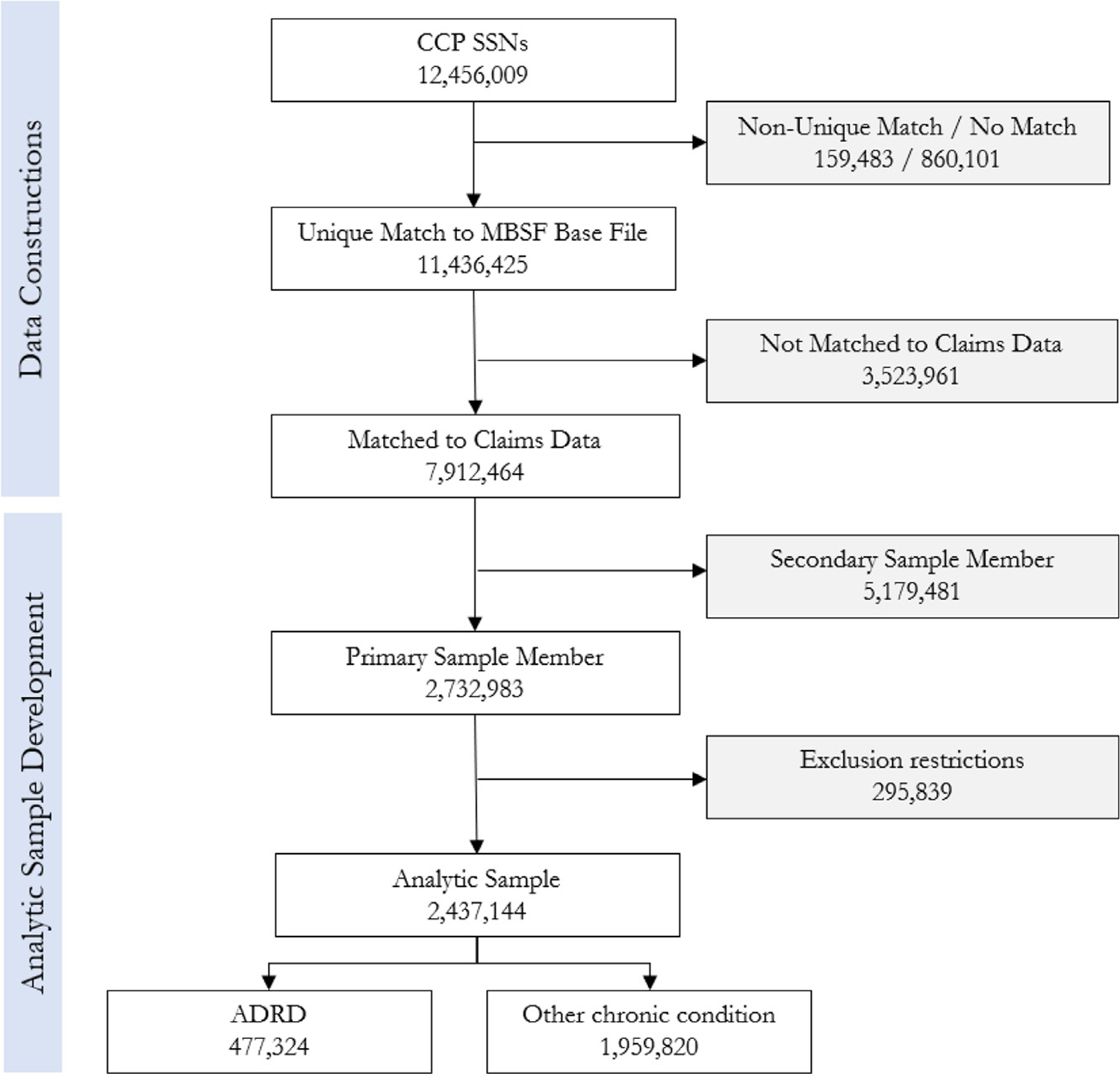
Construction of data and development of analytic sample.

We select a target sample from the CCP that includes individuals age 65 or over during the 2000–2017 timeframe. This sample comprises a “finder file” for searching MBSF data for SSN matches. Fig. 1 profiles information on the data construction process.

The CCP finder file, prepared by Equifax, consists of 12,456,009 SSNs. 91.8% of SSNs (n=11,436,425) in the finder file uniquely match to an SSN in the MBSF Base file. A non-match may occur if the individual is not eligible for or not enrolled in Medicare or if the SSN is not legitimate (6.9% of individuals in the finder file, n=860,101). Additionally, 1.3% of SSNs in the finder file (n=159,483) have a non-unique match, which may occur when multiple individuals claim Medicare benefits under the same SSN or an individual changes the SSN under which they claim benefits (Virnig, 2023).

We obtained Medicare Chronic Conditions and Cost & Use data for enrollees in the matched sample who were ever diagnosed with ADRD or any of the other 25 chronic conditions tracked in the Chronic Conditions Warehouse. Because diagnoses are based on fee-for-service claims, they are available only for individuals enrolled in traditional Medicare, and not for individuals enrolled in Medicare Advantage (MA). During the time frame of our data, between 17 and 35 percent of Medicare enrollees were enrolled in MA and, conversely, between 65 and 83 percent were enrolled in traditional (fee-for-service) Medicare each year (Freed et al., 2021). Correspondingly, we are able to merge Chronic Conditions and Cost & Use data to 69 percent of the enrollees in our matched sample of 11.4M (n=7,912,464).^6^

## 2. Research design

### 2.1. Analytic sample

Our sample includes primary sample members in the CCP (n=2,732,983). These individuals are consistently tracked over time in the CCP, whereas secondary sample members enter or leave the sample depending on whether they remain in the same household as the primary sample member. However, we use information on secondary sample members to characterize the household structure of the primary sample member. We exclude primary sample members who have no credit report data during the study time period or if they only have credit data following their death; individuals who reside in U.S. territories; individuals who are never observed in a household setting (defined as always living in a setting with 100 or more individuals); individuals diagnosed with ADRD prior to age 65, and individuals with ADRD who have an observed date of diagnosis that is immediately preceded by either ineligibility for Medicare due to age or MA enrollment, to avoid potential measurement error in date of diagnosis (specifically, a measured date of onset that is later than in actuality). Our resulting analytic sample includes 2,437,144 individuals. Of these, 477,324 are diagnosed with ADRD during the study time frame (2000–2017) and the remainder have one or more of the other 25 chronic conditions measured in the Chronic Conditions segment, but are never observed with a diagnosis of ADRD. For each sample member, the observation window spans at most 72 quarters (from 2000–2017), but some individuals have shorter windows of observation. We exclude from analysis quarters after death and, for individuals who switch from traditional Medicare to MA, quarters after that transition because we do not observe whether individuals are diagnosed with ADRD while enrolled in MA.^7^

### 2.2. Outcome variables

We examine two outcomes that cross-cut all of an individual’s credit accounts–credit score and any payment delinquency. The credit score measure, Equifax Risk Score 3.0, was developed by Equifax and assesses the likelihood of a consumer becoming seriously delinquent (90+ days past due) over the next 12 months. It is a multi-dimensional construct that can be viewed as an important indicator of an individual’s overall creditworthiness. The score not only captures behavior regarding paying bills (including whether any payment is delinquent as well as the number, length and amount of delinquencies), but also reflects new account origination, debt balance relative to credit available (credit utilization ratio), and credit mix (type of accounts). The score ranges from 280–850, with higher scores representing better credit risk. Generally, credit scores are classified as super-prime (above 720), prime (660–719), near-prime (620–659) and subprime (below 620). We measure delinquency status according to whether the individual is current (paid as agreed) on all accounts or has at least one account that is at least 30 or more days late. For comparison to previous research, we also analyze a dichotomous outcome indicating whether a person has a subprime credit score.

Additionally, we analyze credit card accounts, which are the most common type of revolving account, and mortgage accounts, which are typically installment accounts. With installment accounts, individuals borrow a lump sum and payments on the loan are made on a regularly scheduled basis, while revolving accounts offer access to an ongoing line of credit and a minimum monthly payment is usually required. Credit card accounts and mortgages represent the two largest components of debt among individuals 70 and over (Federal Reserve Bank of New York, 2022). For each of these accounts, we examine any delinquency and a measure of the dollar amount of the delinquent balance among account holders. We also examine the credit card utilization rate, which is the total credit card balance divided by the total credit card limit, and a variable capturing whether the individual’s credit card utilization rate is over 90 percent, which we refer to as being “maxed-out”. High credit utilization is considered a strong predictor of future missed payments.

### 2.3. Empirical strategy

We implement an event study model with individual and time fixed effects following Eq. (1).


(1) $$F_{it}=\gamma X_{it}+\sum_{\tau =t_{i}-J}^{t_{i}+J}{\text{ADRD}}_{i}\cdot 1\left(t=\tau \right)\beta_{\tau }+\lambda_{i}+\theta_{st}+\epsilon_{it}$$

Where *F*_*it*_ represents financial outcomes for individual *i* in time period *t*. *X*_*it*_ includes time-varying demographic and health variables. These include a set of indicator variables capturing age at each observation as well as indicators for household size and probable spouse constructed from information on secondary CCP sample members. We base the presence or absence of a probable spouse on whether there is another adult in the household aged within ±15 years of the primary sample member for households in which there are fewer than 9 members. In addition, we include indicators for each of a set of chronic conditions along with a count of total chronic conditions for each individual in each time period.

*ADRD*_*i*_ is an indicator of whether an individual is ever diagnosed with ADRD, while *t*_*i*_ represents the date of this diagnosis. The specification includes a set of indicators, representing time-distance from the date of ADRD diagnosis, capturing lead and lag effects on the financial outcome. For example, *β*_−1_ represents the change in outcome the quarter prior to diagnosis, while *β*_8_ captures the change in the outcome 8 quarters after diagnosis. The non-parametric event study specification allows the coefficients on each of the lag/lead indicators to vary freely, providing for flexibility in the pattern of outcomes over time relative to diagnosis (Clarke and Tapia-Schythe, 2021). We set the maximum *J* to 28, thus allowing for estimation of the potential effects of early stage ADRD up to 7 years prior to diagnosis. We set the maximum number of lag effects similarly. The variables indicating the timing of the observation prior to diagnosis represent a composite measure of cognitive, personality and neuropsychiatric changes associated with the disease during its lifecycle. Quarters just before diagnosis are associated with greater average symptoms and quarters farther before diagnosis with more limited symptoms based on the typical profile of progression of the disease (Jack et al., 2018; Amieva et al., 2005; Mistridis et al., 2015).

The effects of early stage ADRD on financial outcomes *β*_*τ*_*, τ* = −1,…,−*J* are identified from individual-specific changes over time among those ever diagnosed with ADRD, relative to individual-specific changes over time among those not yet (for at least another 7 years) or never diagnosed with ADRD. These coefficients are the key coefficients of interest and measure the average effect of such changes among individuals who are affected by the disease at each point prior to diagnosis. Some individuals in the comparison group may have had ADRD but never received a diagnosis, which could result in our underestimating the true effects of early stage ADRD. We investigate this in a robustness analysis by estimating our model without the comparison group of the never-diagnosed. In these analyses, the effect of early stage ADRD is identified only from variation in timing of diagnosis and within-individual changes over time in outcomes, as the comparison group is dropped. A limitation of this approach (and the reason we include a comparison group in our main analyses) is that individuals in the comparison group no longer contribute to estimation of the coefficients on other model covariates (such as controls for state-time effects, household structure variables, and chronic conditions). We find qualitatively consistent and statistically significant patterns of results, with coefficient estimates that are generally somewhat larger in magnitude for any delinquency (see Fig. 12).

*λ*_*i*_ represent individual fixed effects, and *θ*_*st*_ is a vector of state-time fixed effects that account for within-state macroeconomic trends.

### 2.4. Estimation

We estimate fixed-effect, propensity score weighted linear regression models as specified in Eq. (1) for continuous outcomes (credit score, delinquent balances). For dichotomous outcomes (delinquency flags), we use fixed-effect, propensity-score weighted linear probability models (Konetzka et al., 2018; Marton et al., 2014). We choose this estimation approach over alternatives (e.g., fixed effect logit or conditional logit models) to avoid issues associated with the incidental parameter problem and facilitate estimation of marginal effects (Angrist and Pischke, 2009; Greene, 2004; Neyman and Scott, 1948). We cluster the standard errors at the individual level in all analyses.

We employ propensity score weighting to adjust for observed differences in covariates between individuals who are ever or never diagnosed with ADRD, reducing the potential for bias in the effects of early stage ADRD we estimate. We develop a propensity score model that includes variables that in theory are likely to influence the probability a person develops ADRD and which may be correlated with financial outcomes (Caliendo and Kopeinig, 2008). Specifically, we account for gender, race/ethnicity, birth cohort, household structure (at age 65), the presence (at age 65) of chronic conditions that are associated with ADRD (depression, hypertension, high cholesterol, and diabetes) (Livingston et al., 2024), state of residence (at age 65), and time period of observation. We also include a set of area level variables, measured at either the Census tract or county level (using location of residence at age 65), designed to proxy for other modifiable risk factors that are associated with dementia (Livingston et al., 2024). These variables capture whether the area is below the median for adult smoking, excessive drinking, adult obesity, physical inactivity, adults who have a high school education or more, and air pollution. Additionally, we include a lagged financial measure – credit score at age 65 – in the propensity score model to control for fixed but unobserved differences in initial conditions across individuals.

We ensure common support by comparing the minimum and maximum of the propensity score distribution for the two groups (those ever diagnosed with ADRD and those never diagnosed with ADRD) and setting the propensity score to missing if it is lower than the minimum or higher than the maximum of the propensity score distribution of the alternative group (Caliendo and Kopeinig, 2008). We trim extreme tails of the distribution of the predicted propensity scores (less than .0001 and higher than .999) and weight our analyses on the (inverse of the) propensity score to obtain a balanced sample of treated and untreated individuals (Abadie, 2005; Imbens, 2004; Wooldridge, 2010). Raw and propensity score-weighted means and the propensity score distribution before and after matching are provided in Supplemental Appendix Table A.1 and Fig. A.1.

Furthermore, we assess the comparability of people who have ever been diagnosed with ADRD and those who have never been diagnosed with ADRD by investigating financial outcome patterns in the two groups in the period before the probable onset of symptoms associated with early stage ADRD (the “pre” period in our context). We examine coefficients on indicators of time to diagnosis for each quarter from 29 to 40 quarters (8 to 10 years) prior to ADRD diagnosis. We use a plausible time period based on clinical findings on the symptom trajectories prior to diagnosis (Vermunt et al., 2019), but a limitation is that we do not know precisely when the average symptoms of early stage ADRD manifest. We find no statistically significant differences in credit scores between those who are never diagnosed with ADRD and those who are ever diagnosed with ADRD during the pre-period. We find no differences in payment delinquency 9–10 years prior to diagnosis and very small differences in the eighth year prior to diagnosis (see Fig. 13), which are detectable given the large size of our data and consistent with the possibility of the presence of some symptoms during this time period. These results provide additional support regarding the comparability of our treatment (ever diagnosed with ADRD) and comparison (never diagnosed with ADRD) group.

### 2.5. Stratified analyses

We also estimate stratified analyses that allow for potential heterogeneity in the effect of early stage ADRD on financial outcomes by household composition, race/ethnicity, and education. We stratify individuals into single and coupled households depending on whether there is another person in the household within +/− 20 years of age in their last quarter of observation. As defined, “coupled” captures seniors who share a household together with other adults of a similar age and may include both traditional (e.g. spouses, romantic partners) and non-traditional partners (e.g. siblings living together). We use this definition to facilitate the comparison of our estimates for single individuals with those from previous research (Nicholas et al., 2021). We measure education using an indicator for whether the Census tract for the location in which an individual resides at age 65 is below the median level of the percentage of adults who have a high school education or more.

Compared to individuals in single households, those who are part of a coupled household may experience more protection against the effects of early stage disease on financial outcomes, depending on the nature of household financial decision-making (Fonseca et al., 2012; Angrisani and Lee, 2019). Among racial minorities and individuals with less education, diagnosis tends to occur later in the cognitive life cycle of the disease and the rate of cognitive decline can be more rapid (Chen et al., 2019; Amieva et al., 2005). As a consequence, symptoms are likely to be more pronounced in any given quarter prior to diagnosis. These differences in symptoms may affect the magnitude of the effect of early stage ADRD at any given point in time on financial outcomes.

### 2.6. Robustness checks and sensitivity analyses

In addition to the robustness checks already described (i.e., analyses that examine differences among the ever or never diagnosed with ADRD in the pre-period before the likely onset of symptoms associated with early stage ADRD), we estimate unweighted models for comparison to our propensity score weighted models. We also estimate models that use only individuals who ever are diagnosed with ADRD. These analyses rely solely on variation in the timing of diagnosis and within-person changes in outcomes among individuals who are ever diagnosed with ADRD to identify the effects of early stage disease.

We also conduct placebo treatment tests (Eggers et al., 2024) in which the independent variables measuring time to ADRD diagnosis are replaced with indicators of time to diagnosis for another condition. These analyses are designed to test the assumption that variables indicating the time to diagnosis of ADRD are capturing cognitive and neuropsychiatric symptoms associated with the disease, and not other unobserved factors that could be associated with time to diagnosis and financial outcomes. Our main placebo tests use cancer (which includes prostate, breast, colorectal, endometrial, and lung), hip/pelvis fracture, and rheumatoid or osteoarthritis (RA/OA). These conditions have not been identified as major risk factors for ADRD (Livingston et al., 2024) and represent placebo test candidates with strong conceptual credibility. We perform additional placebo treatment tests using other conditions including hypertension, hyperlipidemia, depression, diabetes, glaucoma (which causes vision impairment), ischemic heart disease and acute myocardial infarction (AMI), although these conditions have been identified as risk factors for, or share common risk factors with, ADRD (Livingston et al., 2024; Bisciglia et al., 2019; National Heart, Lung, and Blood Institute (NHLBI), 2022). Our analyses of placebo conditions exclude individuals who ever receive a diagnosis of ADRD to help mitigate against potential comorbidity concerns.

We conduct sensitivity analyses in which we include a control for out-of-pocket health care spending. Our main analyses omit this variable because redundant or otherwise unnecessary health care spending represents one channel through which impaired cognitive function from ADRD prior to diagnosis may affect credit outcomes. Nonetheless, we control for out-of-pocket health care spending in a supplemental analysis to account for possible additional health care costs that may be associated with obtaining an ADRD diagnosis or for unusual cases in which treatment is prescribed prior to diagnosis.

Similarly, our main analyses include an indicator for depression at age 65 in our propensity score model, but do not include quarterly indicators for the presence of depression in the main regression model (as we do other chronic conditions) because depression represents a pathway through which ADRD may affect financial outcomes. In additional sensitivity analyses, we include quarterly depression indicators.

Lastly, recognizing the possibility that individuals in the comparison group may be diagnosed with ADRD shortly after the endpoint of the observation window (2017), we also re-estimate our models using only observations through 2013, although the comparison group remains identified using information on health status through 2017.

## 3. Effect of early stage ADRD on financial outcomes

### 3.1. Descriptive results

Tables 1 and 2 provide descriptive results. Our main analytic sample includes 2,437,144 individuals, 477,324 (19.6 percent) of whom are ever diagnosed with ADRD and 1,959,820 of whom have never been diagnosed with ADRD. On average, individuals are observed for 56.3 quarters (approximately 14 years), yielding 137.3 million person-quarter observations in total. The percentage of our sample ever diagnosed with ADRD is 19.6 percent. We use annual ADRD incidence ratios to benchmark our sample. Our estimated ADRD incidence ratios among individuals ages 65–74 (0.9%), 75–84 (2.8%) and 85 and over (6.2%) align with published estimates for AD (Rajan et al., 2019) and for dementia (Olfson et al., 2021).^8^

Race and gender differences are apparent between the ADRD and the non-ADRD sample, underscoring the importance of propensity score weighting. Individuals in the sample ever diagnosed with ADRD are more likely than those never diagnosed with ADRD to be female (60 vs 53.7 percent of person-quarter observations) and Black (8.6 vs 7.9 percent) and a lower percentage of the ADRD sample is Asian (1.3 vs 2.2 percent). Observed race and gender differences are consistent with well-established patterns of prevalence of ADRD (Alzheimer’s Association, 2023). Household structure also varies across individuals in the ever ADRD and no ADRD samples. Individuals in the ever ADRD sample are observed in single person households in 21.3 percent of the quarters they are observed, compared to 17.7 percent of quarters among individuals in the non-ADRD sample. Likewise, in 50.8 percent of observed quarters individuals in the ADRD sample have a probable spouse vs 61.2 percent among the non-ADRD sample. The presence of other health conditions also varies across the ADRD and non-ADRD samples. With few exceptions, the percentage of observations in which other chronic conditions are present is higher among the ADRD sample. For example, glaucoma is present in 11.4 percent of observations in the ADRD sample, compared to 8.6 percent in the non-ADRD sample. Our analyses control for the presence of each of these chronic conditions in each quarter, with the exception of depression which we exclude or include depending on the specification as described in Section 2.6.

Table 2 provides person-quarter level descriptive statistics for outcomes. The mean credit score among the full sample is 745.8.^9^ A 30 day or more delinquency is recorded in 8.1 percent of observations. Credit card delinquency (5.7 percent) is more common than mortgage delinquency (2.5 percent) among account holders of each type although the average balance in delinquency (among delinquent and non-delinquent account holders combined) is larger for mortgages ($2998) vs. credit cards ($352). The mean credit card utilization rate among the full sample of individuals with credit cards is 22 percent and 7.8 percent of individuals with credit cards have a utilization rate greater than 90 percent. Rates of mortgage and credit card delinquency are higher in the ever ADRD compared to the non-ADRD sample.

### 3.2. Effect of ADRD on credit score and any delinquency across all accounts

**Fig. 2.**
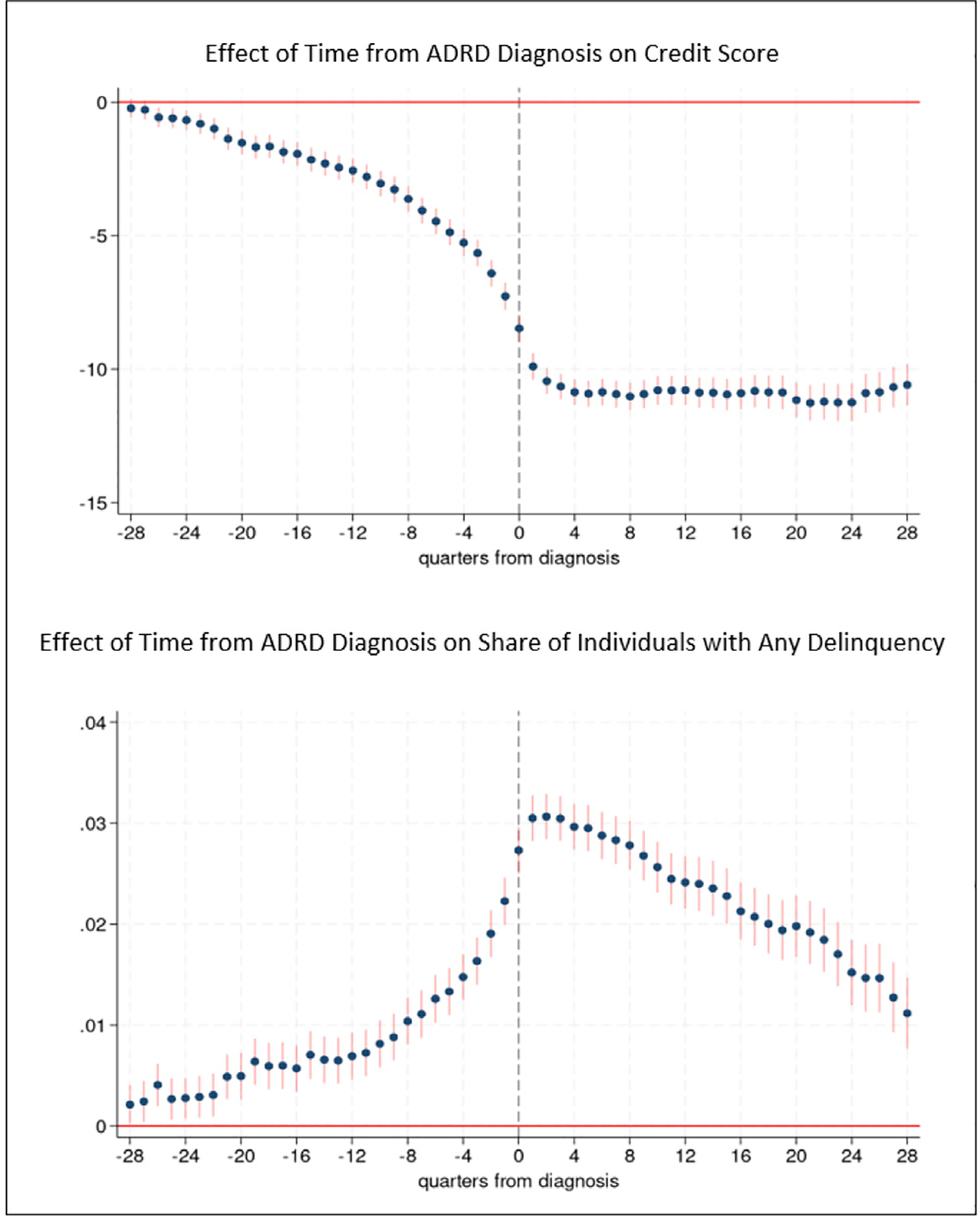
Effect of time from ADRD diagnosis on credit score and share of individuals with any delinquency. Credit score is Equifax riskscore 3.0.

Fig. 2 provides results for the analyses of credit score and any payment delinquency. Tables with coefficient estimates from these models are provided in Supplemental Appendix Table A.2 along with estimates of the propensity score models (Supplemental Appendix Table A.3). In Fig. 2 and other figures, the *x*-axis measures the number of quarters from diagnosis, where *t* = 0 indicates quarter of diagnosis. Although our primary interest is the time period prior to ADRD diagnosis, we also provide post-diagnosis findings. We evaluate the magnitude of effect sizes using baseline averages measured 6 years prior to ADRD diagnosis.

Credit scores decline leading up to ADRD diagnosis (Fig. 2). We observe consistently statistically significant effects during the 6.5 years leading up to diagnosis. In the three to five and one-half years before diagnosis (quarters −9 to −22 in Fig. 2), credit scores are on average 1 to 3.3 points lower than baseline. The magnitude of the effect increases with proximity to diagnosis. In the second year prior to diagnosis (quarters −5 to −8 in Fig. 2), credit scores are on average 3.6 to 4.9 points lower than baseline and are on average 5.3 to 7.3 points lower during the year (quarters −1 to −4 in Fig. 2) just preceding ADRD diagnosis. The spread between the thresholds for super-prime and sub-prime credit scores is 100 points, and between prime and sub-prime is 40 points, which point to the practical significance of the 5 point drop in credit score prior to diagnosis. A 5 point reduction in credit score represents an 0.06 standard deviation decrease in credit score. After diagnosis, credit scores further descend and remain more than 9 points (0.1 standard deviation decrease) below baseline levels during the seven years following diagnosis.

The probability of any payment delinquency increases leading up to ADRD diagnosis (Fig. 2), and, as with credit scores, we find evidence of an effect in the very early stage of the disease, in all quarters up to 7 years prior to diagnosis. The probability of delinquency is 0.49 percentage points higher five years prior to diagnosis compared to baseline, for example. The magnitude of the effect increases for time periods closer to diagnosis. The probability of delinquency is 0.69 percentage points higher three years before diagnosis, 1.0 percentage points higher two years prior to diagnosis, and is between 1.5 and 2.2 percentage points higher during the year just prior to diagnosis. A 1 percentage point change in the probability of delinquency represents a 13.2 percent increase, evaluated at the mean baseline probability of delinquency among individuals with ADRD (7.6 percent). In the six months after diagnosis, the probability of delinquency reaches its highest level, 3.1 percentage points (40 percent) higher than baseline. The probability of delinquency remains more than 2 percentage points higher than baseline during the subsequent three years and then declines but remains elevated compared to baseline levels in the years thereafter.

**Fig. 3.**
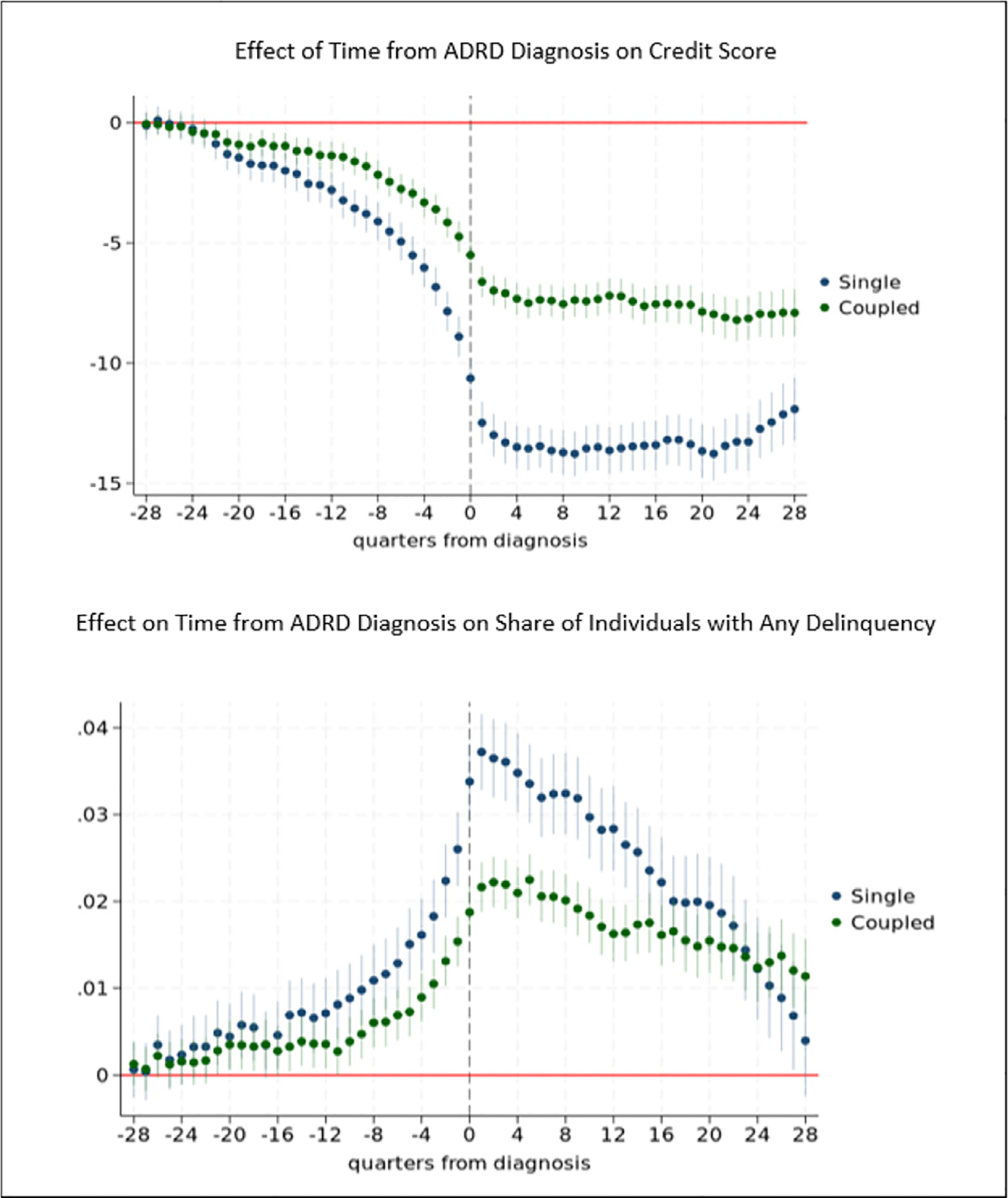
Effect of time from ADRD diagnosis on credit score and share of individuals with any delinquency by household composition.

Fig. 3 shows results stratified by household composition (single vs coupled households). We find that early stage ADRD affects both individuals in single households and those in coupled households and find no or very small (one quarter) differences in the timing of effects of ADRD prior to diagnosis among single and coupled households. The size of effects are frequently larger among single households. For example, two years prior to diagnosis, ADRD decreases credit scores by 4.1 points among singles vs 2.2 points among individuals in coupled households. Effect sizes on the probability of delinquency are similar for single and coupled households until 1.25 years (5 quarters) prior to diagnosis, when effects for single households are larger than those for coupled households.

**Fig. 4.**
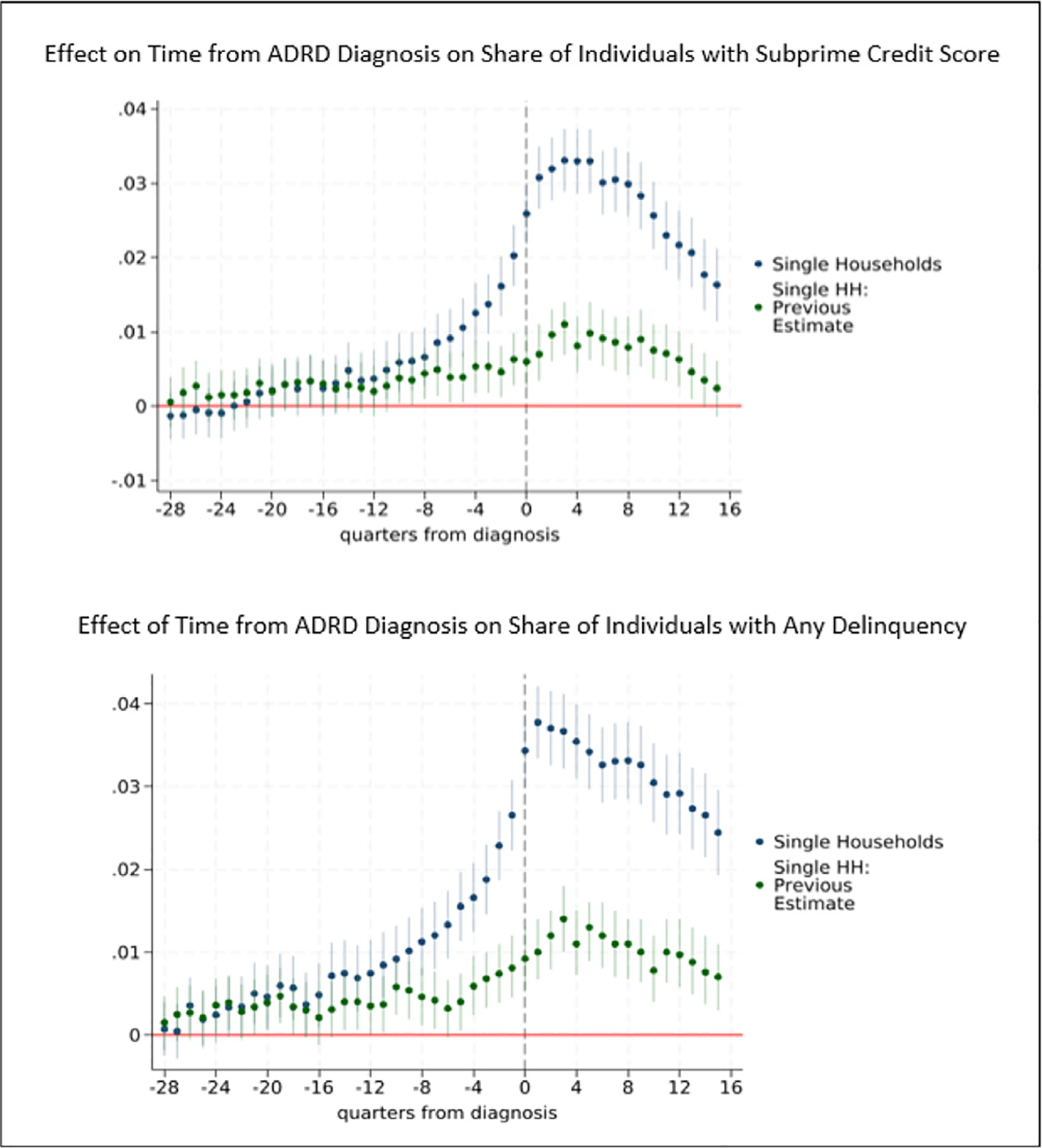
Effect of time from ADRD diagnosis among single individuals on share of individuals with a subprime credit score and share of individuals with any delinquency: Estimate comparison.

Fig. 4 compares our findings for single individuals using the two outcomes previously analyzed and limiting the post-diagnosis quarter indicators to 16 for comparability (Nicholas et al., 2021). Our estimates reveal much larger effects during the year before diagnosis as well as after diagnosis. A year before diagnosis, we find the effect of ADRD on the probability of a subprime credit score is 2.4 times larger and on the probability delinquency is 2.8 times larger. In the quarter prior to diagnosis, our estimated effects are more than three times as large, and at diagnosis the magnitude of our estimated effects are 3.7 to 4.3 times larger.

### 3.3. Effect of ADRD on credit card accounts and mortgages

**Fig. 5.**
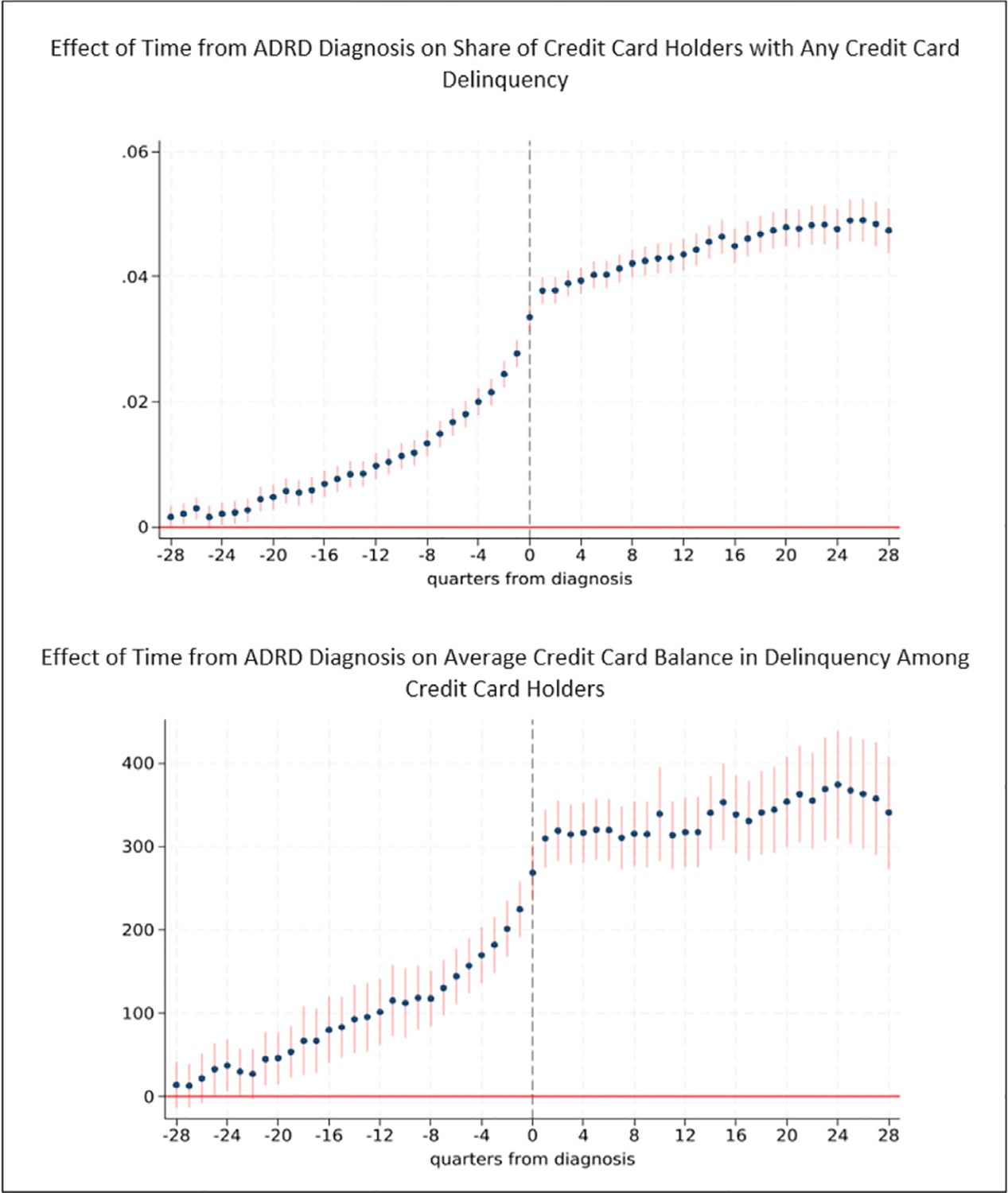
Effect of time from ADRD diagnosis on share of credit card account holders with any credit card delinquency and average credit card balance in delinquency among credit card account holders.

Fig. 5 provides results for the probability of credit card delinquency and average credit card balance in delinquency. Among those with a credit card account we find a consistently higher probability of credit card delinquency during the 24 quarters (6 years) prior to diagnosis, with larger probabilities as diagnosis nears. In terms of magnitude, the probability of credit card delinquency is 0.98 percentage points higher than baseline three years prior to diagnosis. This represents a 17.5 percent increase over the mean baseline probability of credit card delinquency among those with ADRD (5.6 percent). The probability of credit card delinquency is 1.3 percentage points higher two years prior to diagnosis, and in the year prior to diagnosis is 2.0 to 2.8 percentage points (36 to 50 percent) higher than baseline.

Average credit card balances in delinquency, which reflect both the probability of delinquency and the amount delinquent, follow a similar pattern. Balances in delinquency are consistently higher during the 21 quarters prior to diagnosis compared to baseline, and increase with proximity to diagnosis. Three years prior to diagnosis, for example, average credit card balances in delinquency are $101 (30 percent) higher compared to baseline and are $118 (35 percent) higher two years prior to diagnosis. A year prior to diagnosis, average credit card balances in delinquency are $170 (more than 50 percent) higher.

The probability of credit card delinquency among credit card holders continues to increase following diagnosis after diagnosis, and remains more than 4 percentage points higher than baseline thereafter. Average credit card balances in delinquency also remain elevated post-diagnosis, at more than $300 higher per credit card account holder in all quarters compared to baseline.

**Fig. 6.**
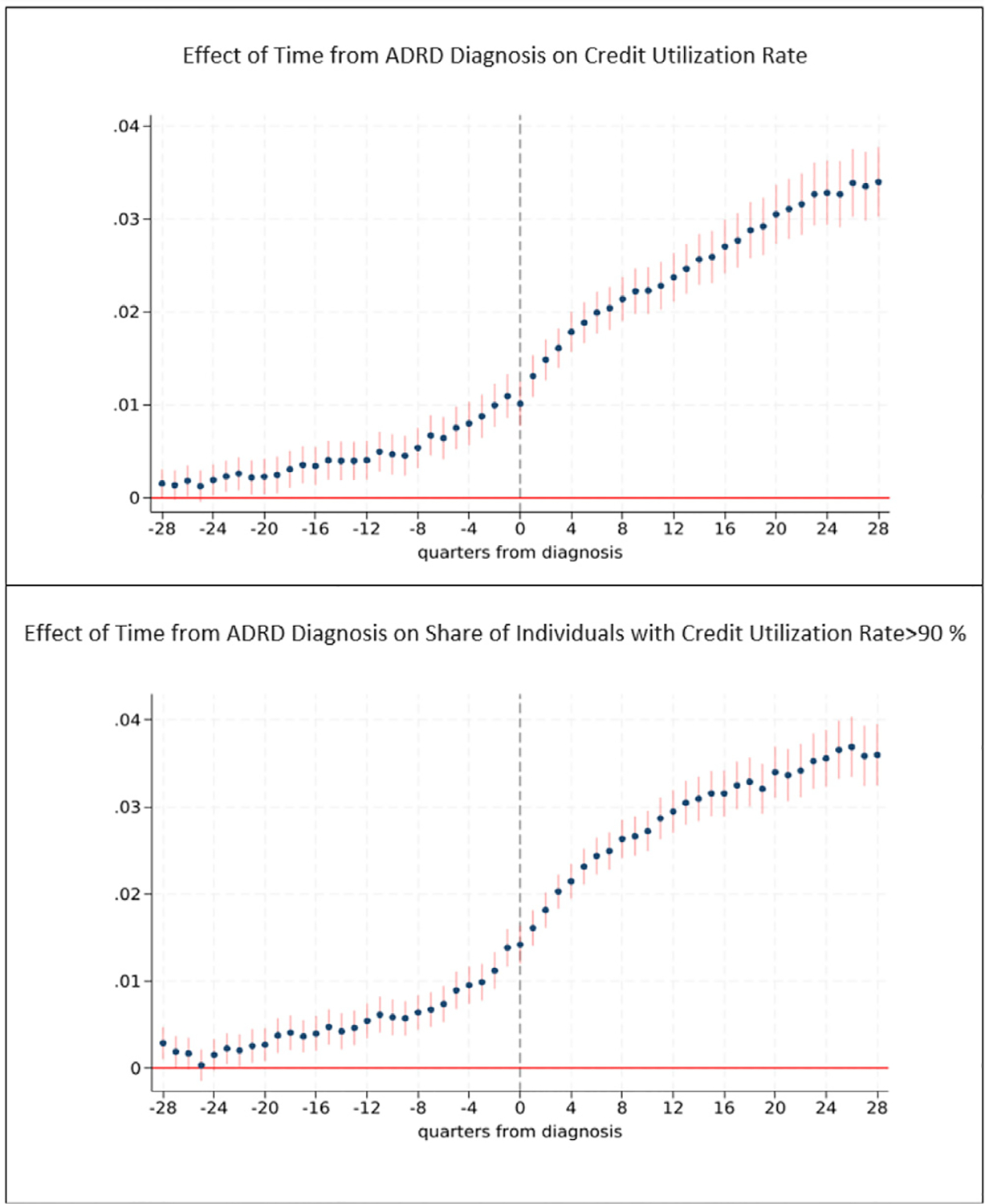
Effect of time from ADRD diagnosis on credit utilization rate and share of individuals with credit utilization rate over 90 percent.

Fig. 6 shows results for the credit card utilization rate. The utilization rate consistently increases starting 24 quarters (six years) prior to diagnosis. A year prior to diagnosis, credit utilization rates are 4.3 percent higher among individuals with ADRD compared to baseline and are nearly 6 percent higher just prior to diagnosis. The bottom panel of Fig. 6 shows results for the effect of ADRD on the probability that a person has a utilization rate over 90 percent, which we refer to as “maxed out” credit card borrowers. The share of individuals who are maxed-out increases 23 quarters (nearly 6 years) prior to diagnosis. The probability that an individual with ADRD is maxed-out is 5.6 percent higher 4 years prior to diagnosis, 13.4 percent higher one year prior to diagnosis, and 19.4 percent higher in the quarter before diagnosis compared to baseline (7.1 percent).

**Fig. 7.**
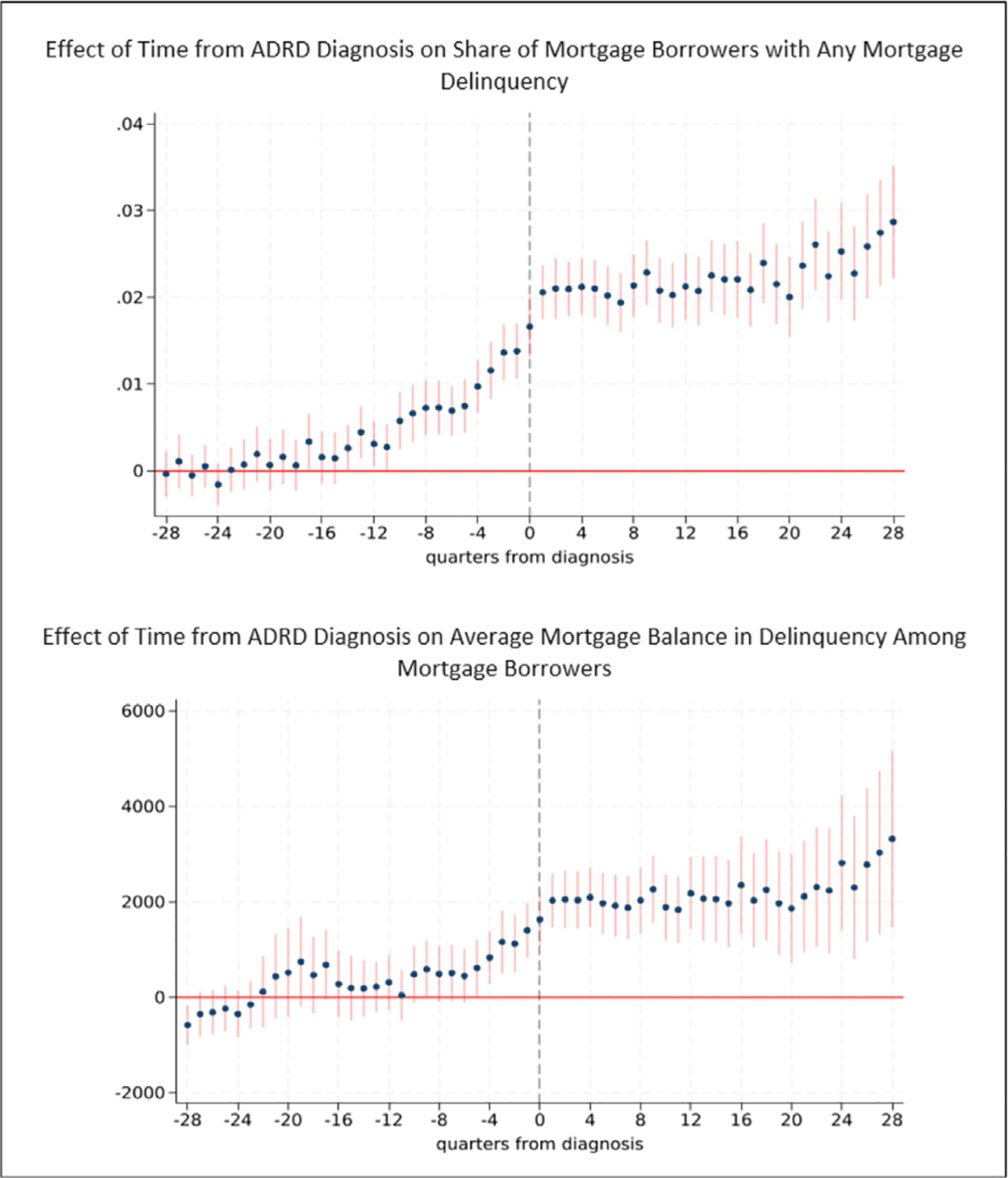
Effect of time from ADRD diagnosis on share of mortgage borrowers with any mortgage delinquency and average mortgage balance in delinquency among mortgage borrowers.

Fig. 7 provides results for the probability of mortgage delinquency and the average mortgage balance in delinquency. The probability of mortgage delinquency is also heightened during early stage ADRD, with consistent effects in the 13 quarters (3.25 years) prior to diagnosis. Effects increase with proximity to diagnosis. Three years prior to diagnosis, the probability of mortgage delinquency is 0.31 percentage points higher than baseline, representing an 11.1 percent increase in the mortgage delinquency rate. One year prior to diagnosis, the probability of a mortgage delinquency is 0.97 percentage points (34.9 percent) higher than baseline. Average mortgage balances in delinquency (which are the product of the probability of delinquency and the amount delinquent) among individuals with a mortgage are higher than baseline during the year prior to diagnosis. They are $829 (24.5 percent) higher one year prior to diagnosis, for example, compared to baseline ($3383). Both the probability of mortgage delinquency and average balance in delinquency for mortgages remain elevated in the years following diagnosis. For instance, five years following diagnosis, average mortgage balances in delinquency are on average $1860 (55 percent) higher compared to baseline.

### 3.4. Effect of ADRD by race/ethnicity and education level

**Fig. 8.**
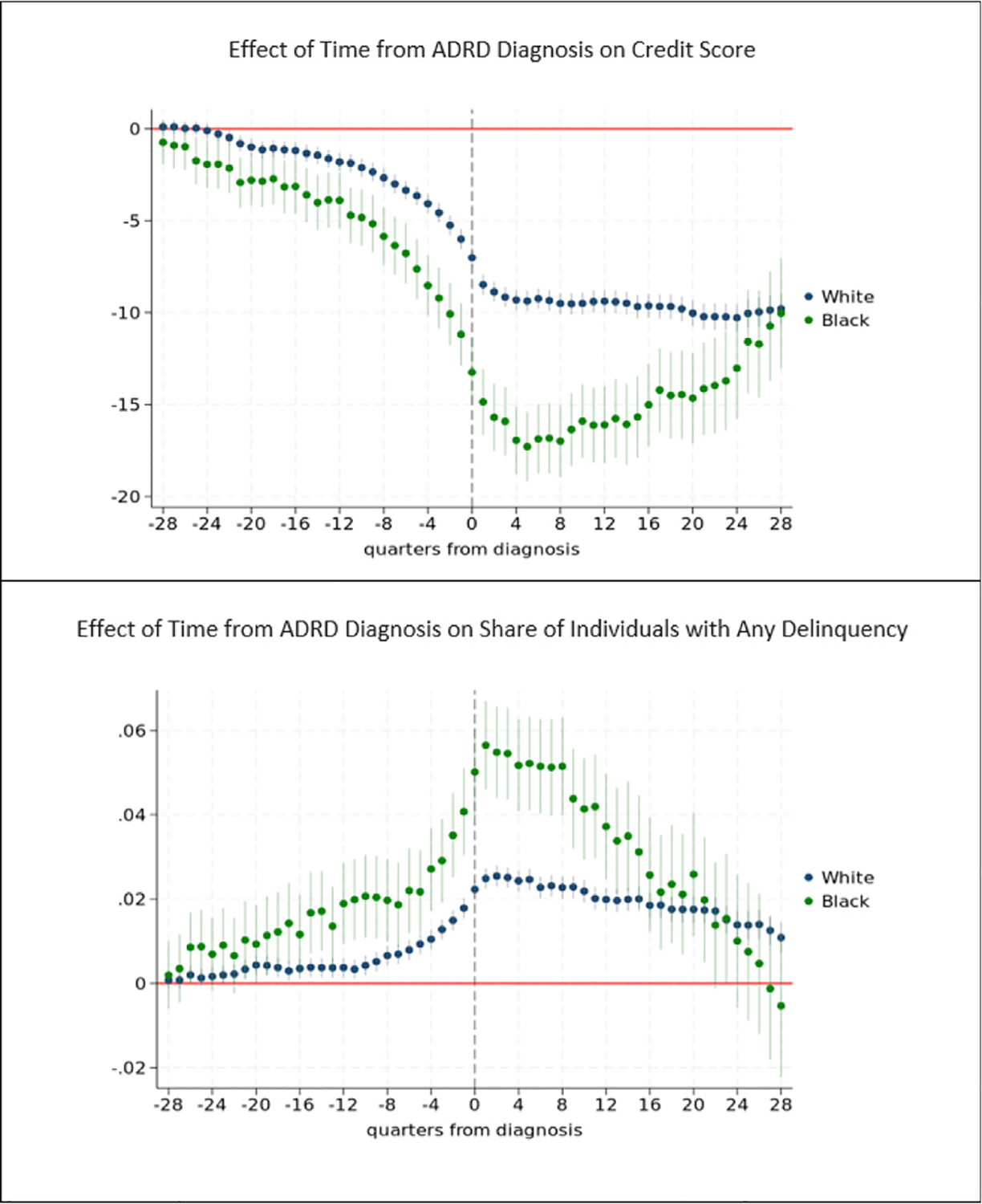
Effect of time from ADRD diagnosis on credit score and share of individuals with any delinquency by race/ethnicity: Non-Hispanic black vs. non-Hispanic white.

**Fig. 9.**
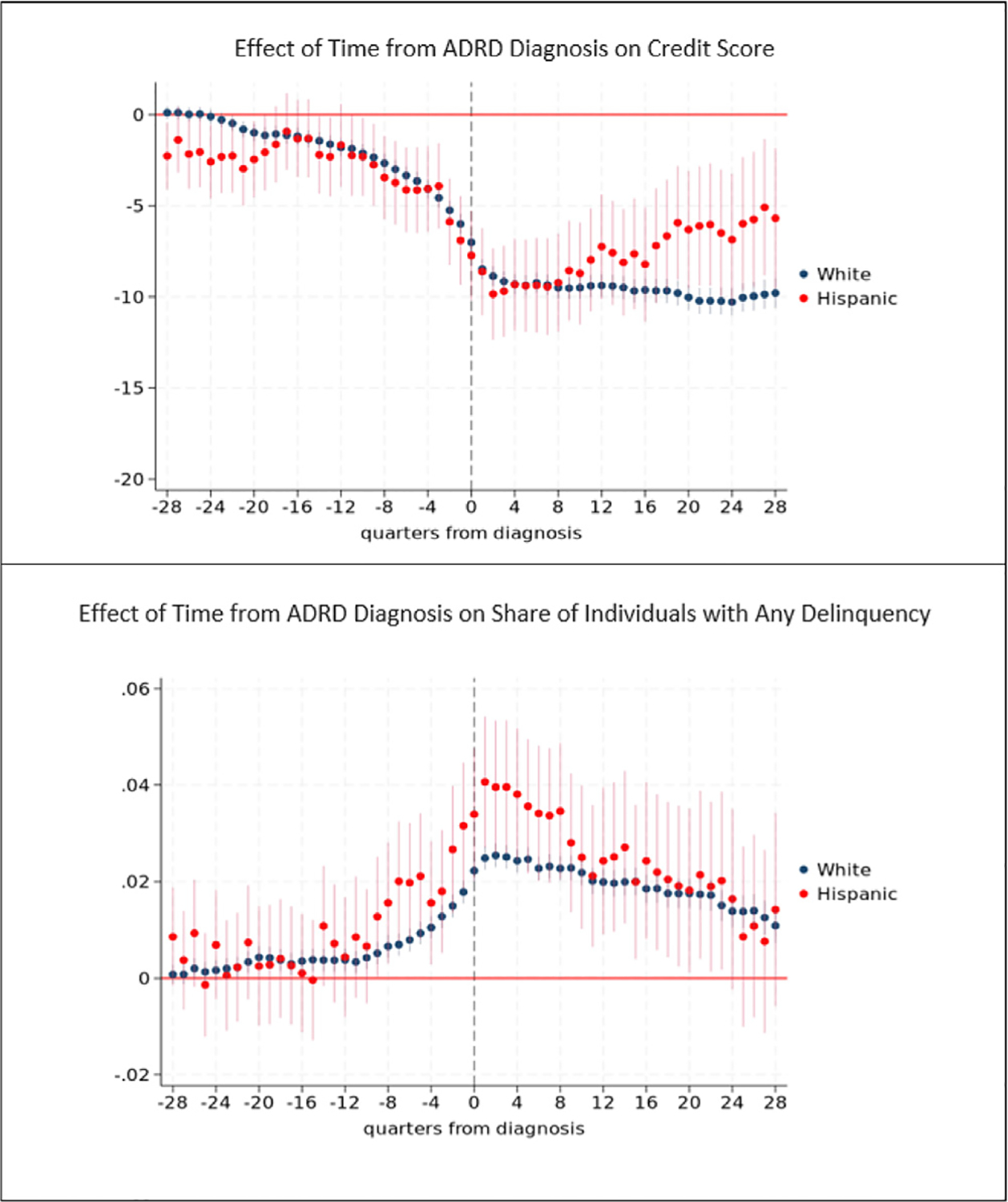
Effect of time from ADRD diagnosis on credit score and share of individuals with any delinquency by race/ethnicity: Hispanic vs. non-Hispanic white.

Figs. 8 and 9 provides results for analyses of the effect of early (pre-diagnosis) stage ADRD on credit score and the probability of any delinquency stratified by race/ethnicity. We focus on results for White, Black, and Hispanic subgroups as limited sample sizes for other racial subgroups (e.g., Asian, other) preclude precise estimation.

The pattern of effects are similar for White and Black subgroups, but the magnitude of the effect of ADRD prior to diagnosis is much larger among Black individuals. Two years (8 quarters) prior to diagnosis, for example, the effect of early stage ADRD on credit scores among Black individuals is more than double that among White individuals (5.8 vs 2.7 point reduction in credit score compared to baseline). One year prior to diagnosis, credit scores are 8.5 points and 4.1 points lower among Black and White individuals, respectively, compared to baseline. Following diagnosis, average credit scores drop to more than 17 points lower than baseline among Black individuals compared to a maximum drop of approximately 10 points from baseline among White individuals.

Similarly, the effects on the probability of any delinquency are on average larger among Black compared to White individuals in quarters prior to diagnosis. Two years prior to diagnosis, early stage ADRD increases the probability of any delinquency 1.97 and 0.6 percentage points among Black and White individuals, respectively. The probability of delinquency is highest for both groups in the few quarters just following diagnosis, but the maximum increase in the probability of delinquency is 5.7 percentage points among Black individuals whereas it is 2.5 percentage points among White individuals.

By contrast, the pattern of effects of ADRD prior to diagnosis on credit scores among Hispanic and non-Hispanic White individuals are similar. Effects in most years after diagnosis are also indistinguishable among these subgroups, as shown by the overlapping confidence intervals in most years. Results are also similar with respect to the probability of delinquency.

**Fig. 10.**
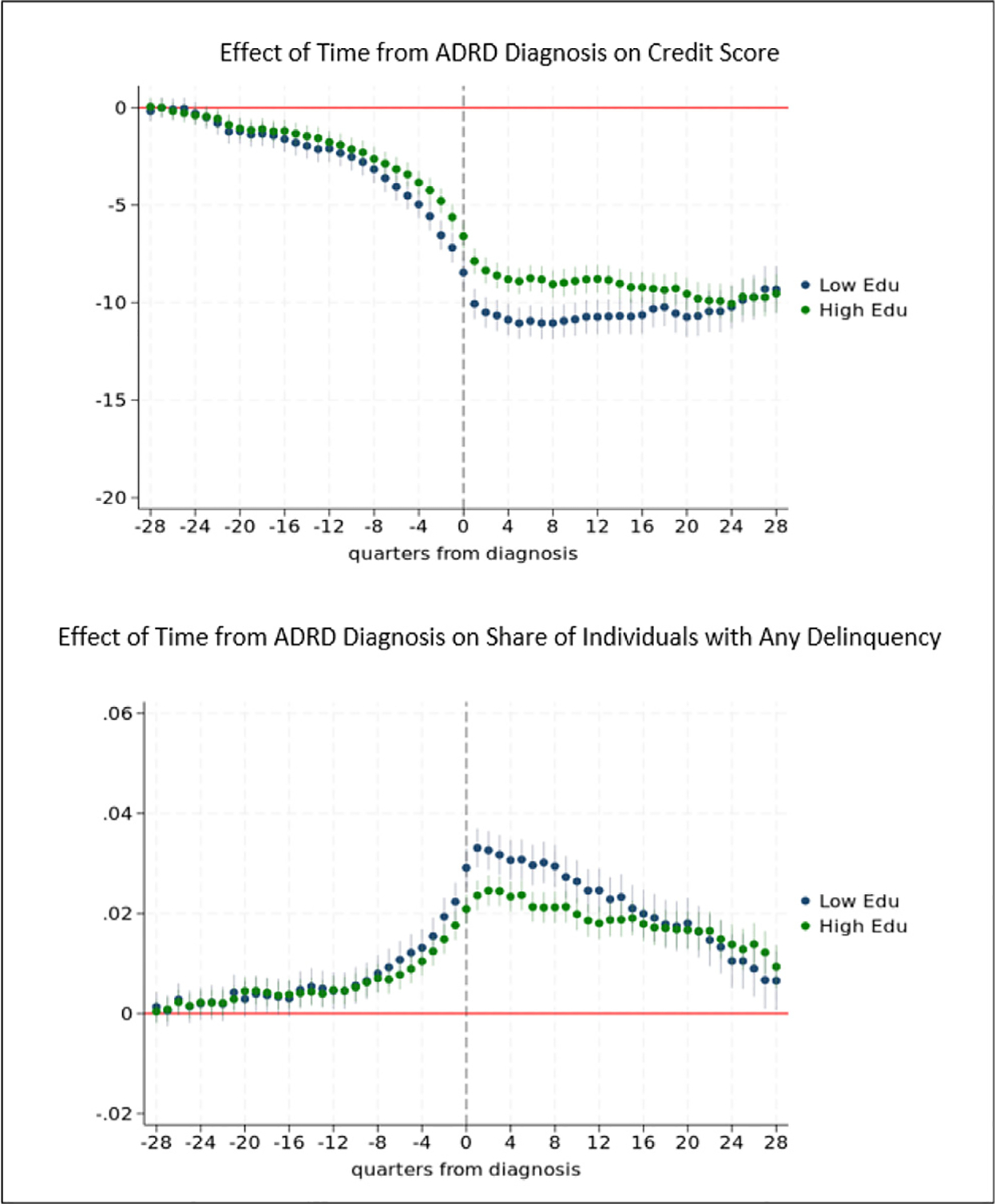
Effect of time from ADRD diagnosis on credit score and share of individuals with any delinquency by education.

Fig. 10 provides results for analyses stratified by education, where education is measured using an indicator for whether the Census tract for the location in which an individual resides at age 65 is below the median level of the percentage of adults who have a high school education or more. We find that ADRD affects individuals in both low and high education areas prior to diagnosis and no or very small (1 quarter) differences in the timing of effects by education. We find that early stage ADRD affects credit scores in the 22 quarters prior to diagnosis and delinquency in the 21 quarters prior to diagnosis among individuals in both low and high education areas. In contrast, Nicholas et al. (2021) find statistically significant effects of ADRD among those in higher education areas only up to 1 year (subprime credit score) or 2.5 years before diagnosis (delinquency). Further, we find no statistically significant differences in the magnitude of effects of ADRD on payment delinquency before diagnosis for individuals in lower vs. higher education areas. For credit scores, the pattern and magnitude of effects are similar for individuals in low vs. high education areas until just before diagnosis. In the two quarters (six months) immediately prior to diagnosis, we find statistically significant differences in effects by education, with larger magnitudes for those in lower vs higher education areas. One quarter before diagnosis, for example, ADRD decreases credit scores by 7.2 points among individuals in low education areas vs. 5.6 points among individuals in high education areas. At diagnosis and for several years after diagnosis, effects on payment delinquency and credit scores are larger for individuals in low education areas. For example, one year after diagnosis, the probability of delinquency is 3.1 points higher than baseline among those with lower education vs. 2.3 points higher among those with higher education.

### 3.5. Robustness checks and sensitivity analyses

**Fig. 11.**
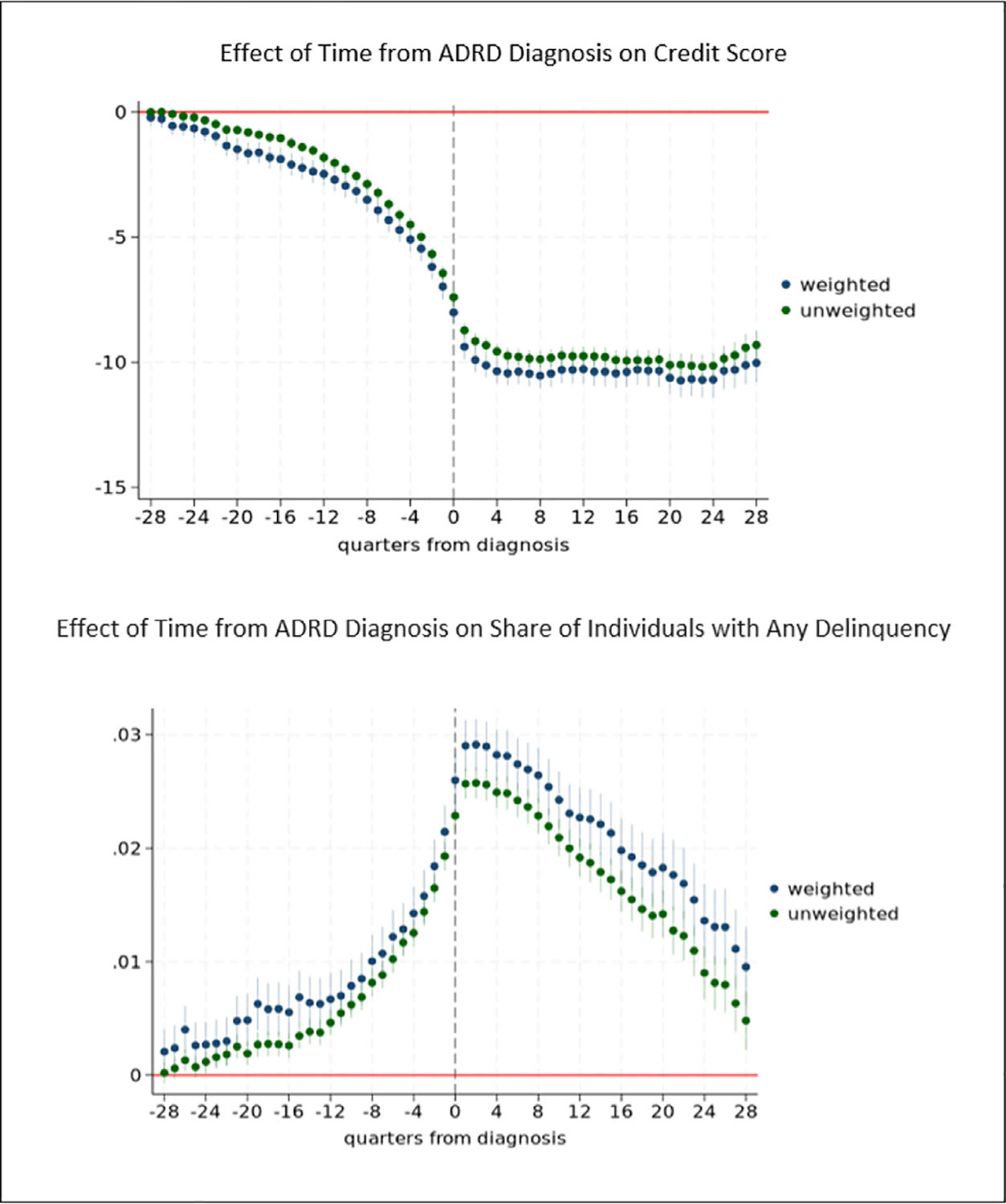
Effect of time from ADRD diagnosis on credit score and share of individuals with any delinquency: Unweighted vs. propensity score weighted results.

**Fig. 12.**
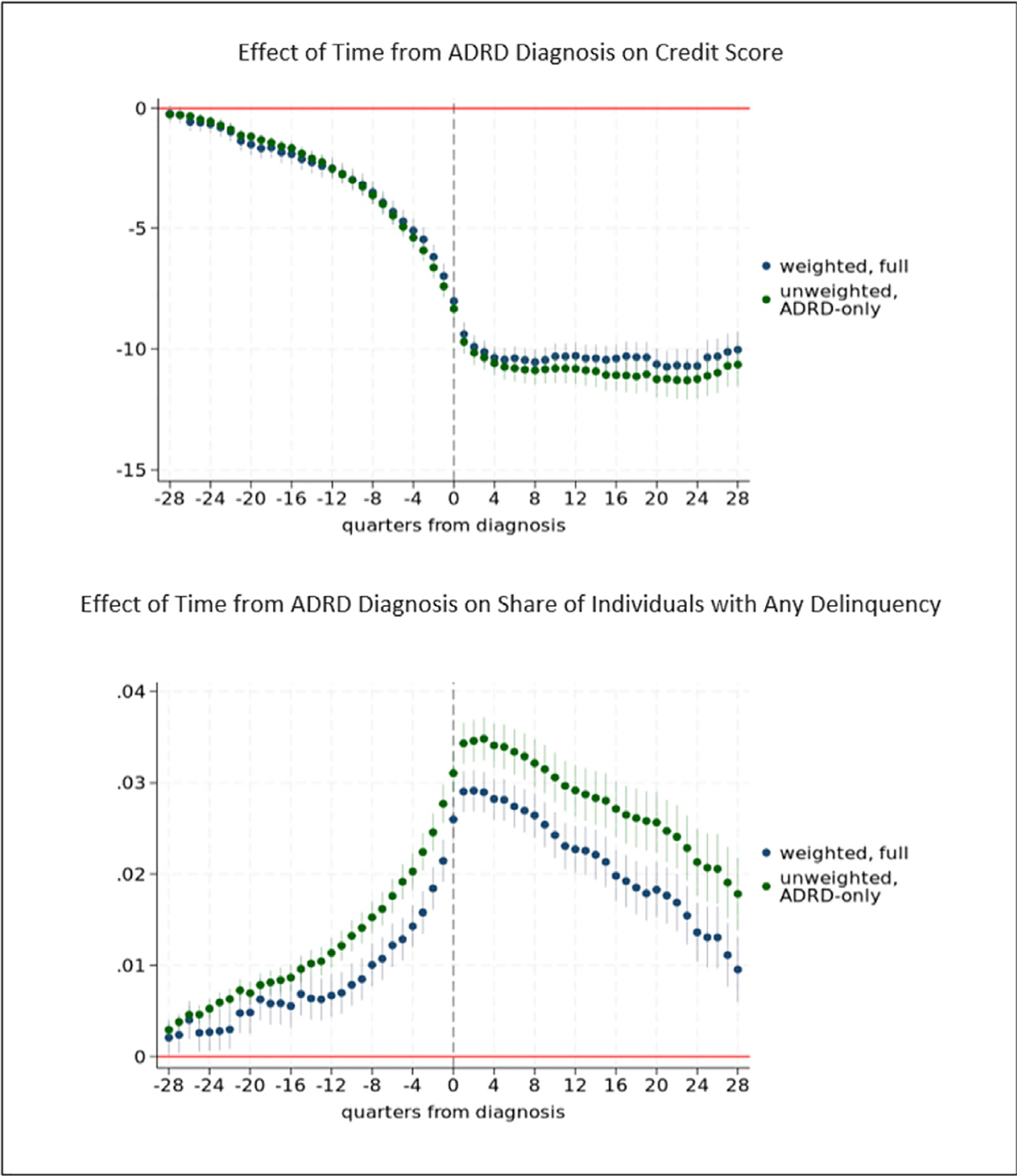
Effect of time from ADRD diagnosis on credit score and share of individuals with any delinquency: ADRD only.

The results are robust in specifications that are unweighted. Fig. 11 compares our propensity score weighted results with unweighted results for credit score and any delinquency. We also compare weighted and unweighted results for other outcomes (Supplemental Appendix Fig. A.2). In analyses that use only individuals who are ever diagnosed with ADRD and drop the comparison group, we find qualitatively consistent and statistically significant patterns of results, with coefficient estimates that are generally somewhat larger in magnitude for any delinquency (Fig. 12).

**Fig. 13.**
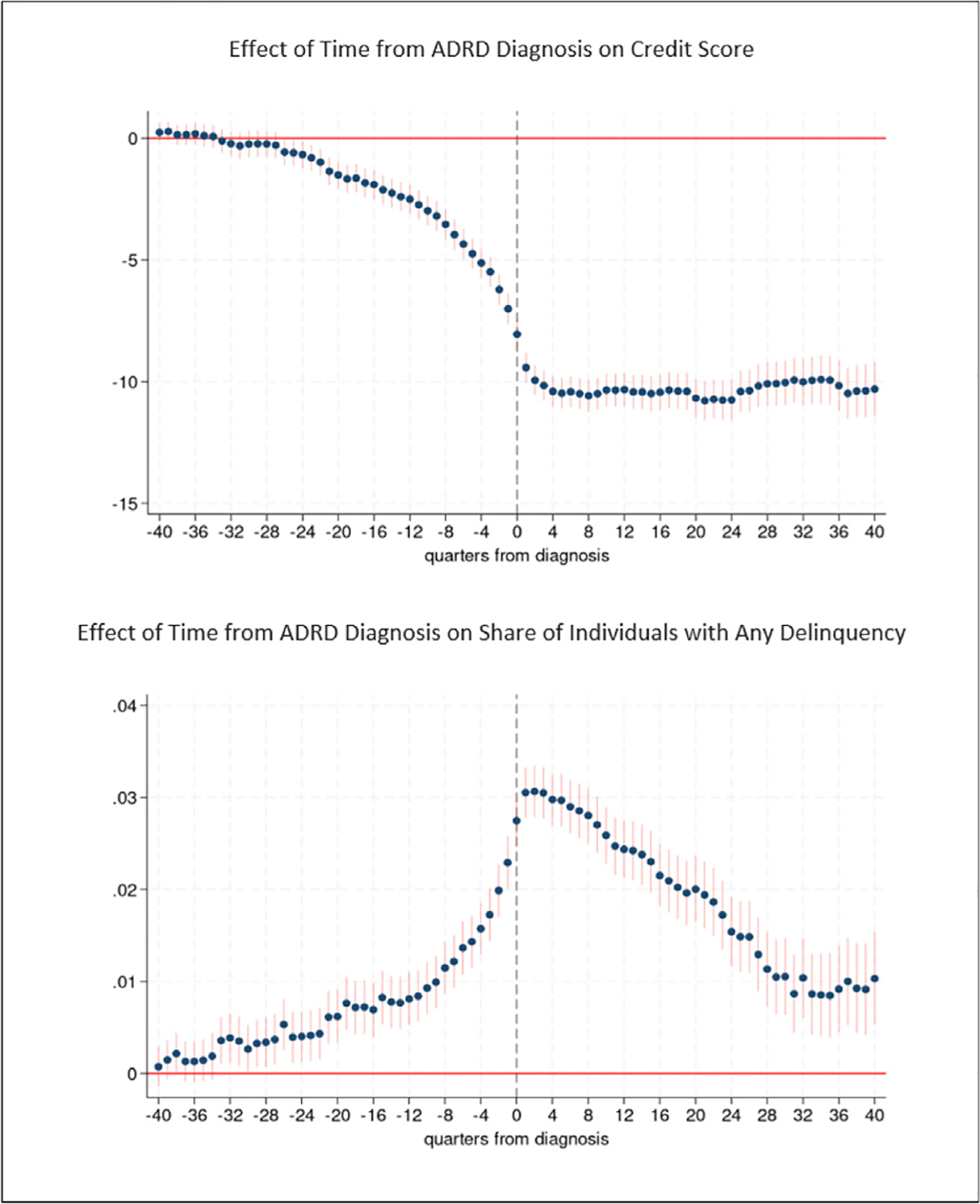
Effect of time from ADRD diagnosis on credit score and share of individuals with any delinquency: Extended time period prior to diagnosis.

We find no statistically significant differences in credit scores between those ever and never diagnosed with ADRD during the pre-period prior to the likely onset of symptoms, which we define as 8–10 years prior to diagnosis, and no or very small differences in payment delinquency (Fig. 13).

**Fig. 14.**
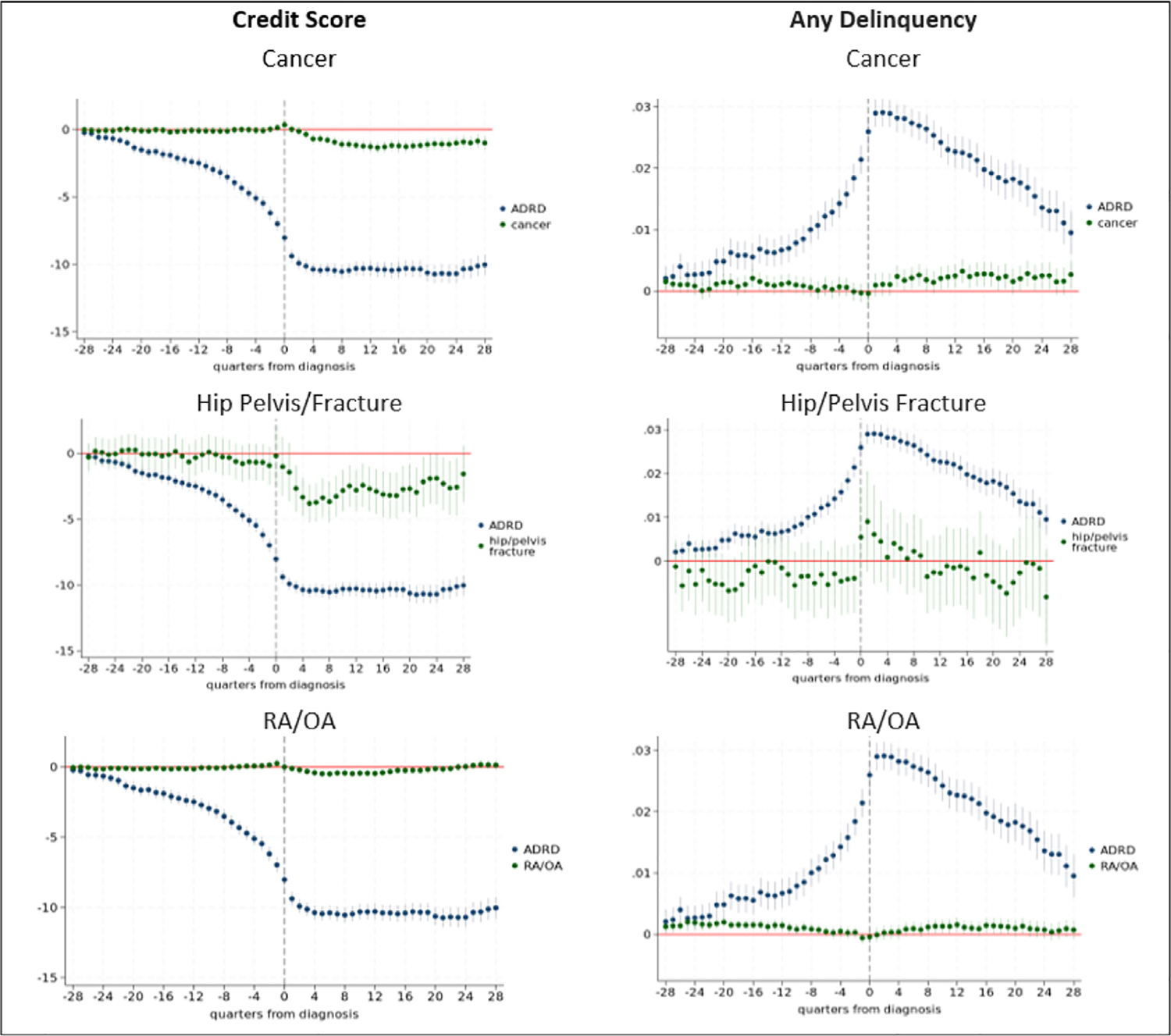
Effect of time from placebo condition (Cancer, RA/OA, Hip/Pelvis fracture) diagnosis on credit score and share of individuals with any delinquency.

In placebo treatment tests, we find no relationship between time to diagnosis and financial outcomes for cancer, hip/pelvis fracture, and RA/OA (Fig. 14). The Supplemental Appendix (Fig. A.7) provides results for other conditions tested. We find, as expected, that time to depression diagnosis is associated with financial outcomes, as this represents a pathway through which ADRD may affect outcomes. Two other conditions (hyperlipidemia and glaucoma) show effects of time to diagnosis on financial outcomes that operate in the same direction as ADRD, but these effects are much weaker.^10^ For glaucoma, these effects may reflect the impact of vision loss associated with the condition on bill paying and financial management (Medicine et al., 2016). For the other conditions (hypertension, ischemic heart disease, diabetes, AMI, one or more of a set of conditions), effect sizes are small relative to those we estimate for ADRD and in the opposite direction of those for ADRD. These findings could reflect reduced consumption from declining health associated with disease onset (e.g., less travel) that could reduce credit utilization and/or improve a household’s ability to repay outstanding credit.

The results are robust in specifications that include out-of-pocket costs (Supplemental Appendix Fig. A.3), include quarterly depression indicators (Supplemental Appendix Fig. A.4), and limit the endpoint of the data to 2013 (Supplemental Appendix Fig. A.5).

## 4. Discussion

Our results can be viewed as an extension of the literature examining the effect of health shocks (such as cancer) on financial well-being (Pak et al., 2020; Richard et al., 2021). Distinctively, however, we study the effect of an undiagnosed condition (memory disorder) for which the primary mechanism of effect is through impaired cognitive function. By contrast, the financial effects of other health shocks often occur after diagnosis from changes in health care spending for treatment.

Average credit scores begin to deteriorate during early stage ADRD, 6.5 years prior to diagnosis, and steadily weaken leading up to diagnosis. Credit scoring formulas are proprietary but a decline in credit scores can follow from the inception of a delinquency, an increase in the number of accounts delinquent, growth in the amount of delinquency, the origination of one or more new credit accounts, an increase in credit utilization rates and/or disproportionate growth in a particular type of credit account (e.g. a surge in credit card accounts as a share of all accounts). The predisposition of individuals with undiagnosed ADRD to these and related behaviors represent a serious potential threat to their financial well-being. This is especially concerning given the substantial financial demands imposed by the disease in its later stages (Hurd et al., 2013). Notably, however, credit scores among those with ADRD do not recover even years after diagnosis, which may reflect the long look-back period used in calculating credit score. The continued suppression of credit scores post-diagnosis may also reflect the negative impact of new resource demands associated with the disease, such as costs of caregiving or supportive living arrangements, on financial solvency.

We also find effects of ADRD prior to diagnosis on payment delinquency, overall and for mortgages and credit accounts, which are the largest components of debt for older adults. The propensity towards delinquency rises the nearer an individual is to diagnosis. These increases in the probability of payment delinquency are large, varying between 14 and 26 percent across total, mortgage, and credit card debt two years prior to diagnosis, for example. Our estimates imply that more than 900,000 delinquencies on some debt will occur over the next 10 years as a consequence of yet-to-be diagnosed ADRD. Although not everyone in early stage ADRD will experience a payment delinquency, for those who do, the scale of the change in delinquency is substantial. One year prior to diagnosis, average credit card balances in delinquency increase by more than 50 percent and average mortgage balances in delinquency are 24 percent higher compared to baseline.

We find that ADRD impacts credit card accounts early in the disease life cycle, as many as six years prior to diagnosis. While automatic bill payment can offer some protection against forgotten payments, credit card and other revolving accounts are less likely to be managed with automatic bill payment compared to installment accounts like mortgages (Greene and Stavins, 2020). At the same time, even when these accounts are managed through automatic payments, individuals with early stage ADRD may be less able to navigate interruptions to automatic bill payment systems, such as from card replacement following fraudulent use. Moreover, the cognitive limitations associated with early stage ADRD may increase the likelihood that individuals experience fraud (Judges et al., 2017). Beyond payment delinquency, we find that early stage ADRD affects individuals’ credit utilization rate during the six years prior to diagnosis. This could occur from accruing balances, which may signal changes in how payment for usual spending is managed, as well from aberrations in spending patterns, like changes in day-to-day or large, one-time purchasing behavior. Later in the disease life cycle, as many as three years prior to diagnosis, we find effects of ADRD on mortgages, a common type of installment account with a predictable repayment obligation. These effects may reflect forgotten bill payment, although automatic bill management is more common among installment accounts (Greene and Stavins, 2020), and also may reflect that individuals with early stage ADRD have become financially overextended and unable to meet their scheduled repayment obligations.

The probability of any delinquency falls after diagnosis, which is consistent with the signaling power that diagnosis may offer. Namely, a diagnosis alerts family members and loved ones that the affected individual is cognitively vulnerable and may require support with financial decision-making. Family members may not only provide oversight and ensure payment for existing debt, but also reduce credit account holding for affected family members.

We find early stage ADRD affects individuals in both single and coupled households. Those in coupled households enjoy some, but not complete protection against the effects of ADRD prior to diagnosis. We find substantially larger effects of early stage ADRD on credit score and the probability of delinquency for Black compared to White individuals, with effects generally estimated to be at least twice as large for Black individuals. Three years prior to diagnosis, Black individuals experience the same percentage decline in credit scores that White adults experience one year prior to diagnosis. These larger effects are compounded by the higher prevalence of ADRD among Black individuals (Chen and Zissimopoulos, 2018). Although access to Medicare at age 65 reduces racial and ethnic disparities in health and access to care, it does not eliminate them (Wallace et al., 2021). One consequence is that diagnosis typically occurs later in the disease life cycle among Black compared to White individuals (Lin et al., 2021; Tsoy et al., 2021), resulting in greater average symptoms at any point in time prior to diagnosis among Black adults. Our findings may also reflect the generally greater financial vulnerability among Black individuals.

We find that early stage ADRD affects individuals with higher education levels (proxied by the education level in the local area), as well as those with lower education levels, and we find no differences in the timing of effects by education. We also find limited differences in the pattern and magnitude of effects prior to diagnosis among those in areas with higher and lower levels of education. In the several years following diagnosis, effects are larger in magnitude among those with lower education levels. These heightened effects may reflect the generally more fragile financial standing of individuals with less education.

### 4.1. Limitations

There are several limitations to our research. First, Medicare claims data identify individuals who are diagnosed with ADRD, and not all individuals affected by ADRD receive a diagnosis. The consequence is that some individuals in our comparison group may have ADRD even though they have not received a diagnosis, which may understate the effect of ADRD on financial outcomes. Some physicians, for example, may not document the disease (Koller and Bynum, 2015). Amjad et al. (2018) find that individuals who are Hispanic, have less than high school education, attend medical visits alone, or have fewer functional impairments are more likely to be undiagnosed. The estimated sensitivity and specificity for Medicare claims data-based diagnoses of dementia are reassuring (Taylor et al., 2009). Moreover, we conducted analyses in which we drop the comparison group and include only individuals ever diagnosed with ADRD. In these analyses, we find qualitatively consistent and statistically significant patterns of results, with coefficient estimates that are generally somewhat larger in magnitude (Fig. 12), consistent with a modest attenuation bias due to ADRD underdiagnosis.

By including individual fixed effects, our estimation approach accounts for non-time varying individual characteristics – observed and unobserved – that may influence financial outcomes. We also control for a robust set of time-varying covariates, including indicators for the presence or absence of a number of chronic conditions. Although our main analyses exclude depression indicators for each quarter because depression represents a pathway through which ADRD may affect financial outcomes, our results are robust in analyses that include these indicators, with only slightly attenuated effect sizes (Supplemental Appendix Fig. A.4). We use propensity score weighting to account for observable differences between individuals who develop ADRD and those who do not, and our propensity score weighted means are similar across our treatment and comparison groups (Supplemental Appendix Table A.1). A limitation is that we use area-level measures of health behaviors such as smoking and alcohol consumption in our propensity score model as proxies for individual health characteristics (at age 65). Additionally, although it is impossible to rule out the existence of other, unobservable differences between these groups, we test the comparability of patterns in financial outcomes among individuals ever or never diagnosed with ADRD during the time period before the likely onset of symptoms associated with early stage ADRD. These analyses show similar patterns in outcomes among our treatment and comparison groups in the “pre” period (Fig. 13), which further buttress the validity of our approach and the interpretation of our estimates as causal effects.

We acknowledge the possibility that poor financial outcomes could hasten diagnosis if family members recognize the linkage between financial events and the possibility of an impairment and seek physician advice. This would be most likely to occur when those financial difficulties are more salient, such as when individuals experience bankruptcy, foreclosure, or the imposition of a lien. These are, however, rare events affecting fewer than 2 percent of individuals with ADRD in our sample. Additionally, the relationship between adverse financial outcomes and ADRD has only recently become recognized and thus is not likely to have been well-appreciated by families during the time period of our study.

Further, some individuals in our control group may be diagnosed with ADRD after the endpoint of our observation window. The extent to which this occurs represents contamination in the control group and leads to a downward bias on our estimates. Consistent with this, sensitivity analyses in which we identify the control and treatment groups using information on conditions through 2017 and drop observations after 2013 show effect sizes that are somewhat stronger in magnitude.

Our time to diagnosis variables proxy for the composite symptoms associated with ADRD at a given point in the disease life cycle relative to diagnosis. We recognize that the trajectory of symptoms can vary across individuals, both as a result of differences in the speed of diagnosis and differences in the slope of changes. Our main results should be understood as the effect of the average symptoms associated with each time period prior to ADRD diagnosis for those diagnosed with ADRD, while the stratified analyses we conduct by race and education provide insight into heterogeneity in the effects of ADRD prior to diagnosis consequent to differences in symptom severity.

Our data exclude individuals in Medicare Advantage plans, whose health care service utilization is tracked in encounter data vs. claims records. During the time frame of our data, fee-for-service accounted for the majority (between 65 and 83 percent) of Medicare enrollment (Freed et al., 2021) and claims data have been the basis of research for decades. The Center for Medicaid and Medicare Services (CMS) recently began releasing encounter data for Medicare Advantage enrollees; however, the completeness of these data has been identified as an issue and more generally the strengths and limitations of these data are only beginning to be understood (Jung et al., 2022).

Additionally, some individuals have no credit history and thus will not be represented in credit data. These may include, for example, individuals who are undocumented (who also will lack Medicare enrollment/claims data). However, credit data is available for the vast majority – an estimated 89 percent – of U.S. adults (Brevoort et al., 2015). Moreover, the exclusion of individuals without credit accounts from our analysis does not represent a potential source of bias in our estimates of the effect of ADRD on credit outcomes (since the universe of people with credit scores and credit accounts are included in analysis). Finally, credit history and credit scores can vary across credit bureaus and our data rely on only one source (Equifax). But, differences across the bureaus in their data are largely idiosyncratic, individual level differences, such as those related to disputes over particular items that may be removed from one but not another credit report, as opposed to systematic differences related to method or scope of data collection (Avery et al., 2003).

### 4.2. Conclusions

Our findings are important for several reasons. First, the adverse effects of undiagnosed ADRD on credit scores and payment delinquency carry consequent financial costs in both the short- and long-term. Immediate pecuniary costs include late fees and interest charges associated with payment delinquency, while future costs may include reduced access to the credit market and less advantageous terms of available credit such as restricted credit limits and – because credit scores are used by lenders in pricing credit – higher interest rates on credit cards and personal loans (Brevoort et al., 2020). Additionally, delinquency on mortgage payments can be a precursor to eventual foreclosure, a costly event for both households and communities (Gallagher et al., 2019; Campbell et al., 2011).

For individuals and households facing a memory disorder diagnosis, simultaneously confronting diminished access to credit, poorer credit terms, and/or a heightened potential of foreclosure is likely to substantially exacerbate an already destabilizing circumstance. Once diagnosed, average dementia-related caregiving and health care costs run in the tens of thousands of dollars annually (Hurd et al., 2013). As such, demand for credit among individuals and households affected by memory disorders may increase just as its supply is restricted.

Our findings lend urgency to the need for further consideration of the roles government agencies and financial institutions can play in reducing financial risk among older households with undiagnosed memory disorders (Chandra et al., 2023; DeLiema et al., 2020; Wood and Lichtenberg, 2017). The legal and regulatory framework for preventing elderly financial exploitation varies depending on the financial actors involved (i.e., investment advisors, banks, credit unions, money services businesses, credit card companies), but common elements include allowing institutions to delay suspicious transactions, recommending or requiring reporting, and promoting trusted contacts (Bureau, 2016). Trusted contacts offer account holders the opportunity to identify a third party who can be notified in the event of suspicious financial activity. For credit card companies, laws such as the Fair Credit Billing Act (FCBA) and Electronic Fund Transfer Act (EFTA), in conjunction with industry standards and state consumer protection laws, influence detection and reporting practices.

Regulation designed to mitigate elder financial exploitation has evolved in the last decade, including states’ adoption since 2016 of laws granting financial professionals authority to reach out to trusted contacts and delay suspicious transactions (following the North American Securities Administrators Association Model Act) (Carlin et al., 2023), and the 2018 implementation of Financial Industry Regulatory Authority (FINRA) regulations 2165 and 4512, which address the use of trusted contacts, transaction holds, and reporting for broker-dealers. Previous research finds that state laws adopted between 2016–2020 reduced financial abuse among seniors (Carlin et al., 2023). Whether these laws and other recent regulatory changes meaningfully affect adverse credit outcomes among people with undiagnosed memory disorders is an area for future research that requires additional information extending beyond the 2017 endpoint of our data.

Importantly, the adverse financial outcomes we observe could reflect not only the increased susceptibility of those with early stage ADRD to fraud and exploitation, but also the effect of ADRD on individuals’ own financial behavior, absent manipulation or malfeasance from others. In the case of the latter, trusted contacts may be especially salient in terms of preventing adverse financial outcomes. Previous work has also considered other possibilities. Moulton et al. (2014), for example, point to the potential of low-cost interventions in credit markets such as external monitoring while Agarwal et al. (2009) describe options that include financial “drivers’ licenses”, advance directives, and expanded fiduciary requirements.

Furthermore, our findings point to the importance of earlier diagnosis as a tool to help reduce or prevent poor financial outcomes. Agarwal and Muckley (2024) find money management difficulty can help identify vulnerable individuals with early stage dementia. Our findings further support the possibility that information on changes in financial behavior could be effectively used to identify individuals at risk for ADRD who should receive additional clinical evaluation.

## Supplementary Material

Appendix

Data Replication Files

## Figures and Tables

**Table 1:** Descriptive statistics.

	Full Sample		Ever ADRD		No ADRD	
	Mean	(std dev)	Mean	(std dev)	Mean	(std dev)
Demographics						
Female	0.548	0.498	0.600	0.490	0.537	0.499
White	0.832	0.374	0.852	0.355	0.828	0.377
Black	0.080	0.272	0.086	0.280	0.079	0.270
Hispanic	0.045	0.208	0.039	0.194	0.047	0.211
Asian	0.020	0.141	0.013	0.114	0.022	0.146
Other race/ethnicity	0.022	0.147	0.010	0.100	0.025	0.155
Low education (census tract)	0.427	0.495	0.447	0.497	0.422	0.494
Household structure						
Household size = 1	0.184	0.387	0.213	0.409	0.177	0.382
Household size = 2	0.397	0.489	0.383	0.486	0.400	0.490
Household size = 3	0.205	0.404	0.192	0.393	0.208	0.406
Household size = 4	0.097	0.296	0.091	0.288	0.098	0.297
Household size = 5	0.043	0.203	0.042	0.200	0.043	0.203
Household size = 6 or more	0.075	0.263	0.080	0.271	0.074	0.261
Probable spouse	0.593	0.491	0.508	0.500	0.612	0.487
Conditions						
Acute myocardial infarction (AMI)	0.006	0.080	0.009	0.095	0.006	0.074
Anemia	0.173	0.379	0.261	0.439	0.145	0.352
Asthma	0.034	0.182	0.041	0.198	0.032	0.177
Atrial fibrillation	0.068	0.252	0.103	0.304	0.057	0.232
Breast cancer	0.027	0.162	0.031	0.173	0.026	0.158
Colorectal cancer	0.012	0.107	0.014	0.119	0.011	0.103
Endometrial cancer	0.002	0.048	0.002	0.048	0.002	0.048
Lung cancer	0.008	0.088	0.007	0.084	0.008	0.089
Prostate cancer	0.032	0.176	0.037	0.188	0.030	0.171
Cataracts	0.202	0.402	0.237	0.425	0.191	0.393
Chronic heart failure (CHF)	0.119	0.324	0.194	0.396	0.095	0.293
Chronic kidney disease	0.101	0.302	0.141	0.348	0.089	0.284
Chronic obstructive pulmonary disorder (COPD)	0.088	0.284	0.120	0.325	0.078	0.268
Depression	0.090	0.286	0.158	0.364	0.068	0.251
Diabetes	0.216	0.411	0.260	0.439	0.201	0.401
Glaucoma	0.092	0.290	0.114	0.317	0.086	0.280
Hip or pelvis fracture	0.005	0.073	0.013	0.113	0.003	0.054
Hyperlipidemia	0.384	0.486	0.420	0.494	0.372	0.483
Hyperplasia (benign prostatic)	0.054	0.226	0.067	0.249	0.050	0.218
Hypertension	0.481	0.500	0.594	0.491	0.445	0.497
Hyperthyroidism (acquired)	0.109	0.312	0.145	0.352	0.097	0.297
Ischemic heart disease	0.268	0.443	0.367	0.482	0.235	0.424
Osteoporosis	0.054	0.227	0.085	0.279	0.044	0.206
Rheumatoid/osteoarthritis	0.244	0.430	0.327	0.469	0.218	0.413
Stroke/transient ischemic attack (TIA)	0.031	0.174	0.065	0.247	0.020	0.140
Count of chronic conditions	1.992	2.584	3.707	3.023	1.615	2.313

Notes: Summary statistics are calculated using person-quarter observations. Chronic conditions indicate the presence of a condition in the quarter. Full sample includes 2,437,144 individuals (137,252,711 person-quarter observations). 477,324 individuals (24,704,856 person-quarters) are in the ever diagnosed with ADRD group. 1,959,820 individuals (112,547,855 person-quarters) are in the never diagnosed with ADRD group. Low education census tract indicates a tract with a below-median percentage of the population age 25 and over with a high school diploma or more.

**Table 2:** Descriptive statistics: Financial outcomes.

	Full sample		Ever ADRD		No ADRD	
	Mean	Std Dev	Mean	Std Dev	Mean	Std Dev
Credit score	745.8	86.3	749.4	84.0	745.0	86.8
Any delinquency	0.081	0.272	0.082	0.274	0.080	0.272
Hold a credit card account	0.835	0.371	0.779	0.415	0.847	0.360
Any credit card delinquency^a^	0.057	0.231	0.064	0.245	0.055	0.228
Delinquent balance credit card^a^	352.3	3,126.0	393.1	3,148.5	344.1	3,121.4
Credit card utilization rate^a^	0.220	0.311	0.185	0.301	0.227	0.312
Credit card utilization rate > 90 percent^a^	0.078	0.269	0.074	0.262	0.079	0.270
Hold a mortgage account	0.305	0.46	0.177	0.382	0.333	0.471
Any mortgage delinquency^b^	0.025	0.156	0.031	0.172	0.024	0.154
Delinquent balance mortgage^b^	2,998.2	35,523.8	3,177.0	32,311.3	2,977.4	35,879.8

a Among credit card holders.

b Among mortgage borrowers.

Note: Calculated using person-quarter observations. The full sample includes 137,252,711 observations. The ADRD sample includes 24,704,856 and the non ADRD sample 112,547,855 observations.

## Data Availability

Financial Consequence_Replication (Original data) (Mendeley Data)

## Footnotes

3 ADRD typically includes subtypes such as vascular dementia, frontotemporal dementia and some types of unspecified dementia. Dementias that are temporary, caused by infection, or associated with traumatic brain injury, for example, are typically not included in ADRD. Specific symptoms can vary across individuals and with different subtypes of ADRD (National Institute on Aging (NIA), 2023), but ADRD is progressive and cognitive impairment becomes more severe over time.

4 We use the 27 CCW Chronic Conditions version of the file that spans 1999–2021. The conditions include acute myocardial infarction; cataract; rheumatoid/osteoarthritis; chronic obstructive pulmonary disease (COPD); atrial fibrillation; heart failure; diabetes; depression; glaucoma; osteoporosis; stroke/transient ischemic attack; chronic kidney disease; hip/pelvic fracture; ischemic heart disease; various cancers (breast, colorectal, endometrial, lung, prostate); anemia; asthma; hyperplasia (benign prostatic), hyperlipidemia; hypertension; and hyperthyroidism (acquired).

5 The ICD9-CM diagnosis codes for ADRD are: 331.0, 331.11, 331.19, 331.2, 331.7, 290.0, 290.10, 290.12, 290.13, 290.20, 290.21, 290.3, 290.4, 290.41, 290.42, 290.43, 294.0, 294.10, 294.11, 294.8, and 797. Beneficiaries must have at least one inpatient, skilled nursing facility, home health, Part B institutional, or Part B non-institutional claim with specified diagnosis code in any position.

6 Those not matched to claims data also include some individuals enrolled in fee-for-service who were never diagnosed with one of the 27 chronic conditions in the Chronic Conditions Warehouse. However, the vast majority of individuals 65 and over have at least one chronic condition, as (85.6%) have one or more of six chronic conditions (diabetes, asthma, COPD, cancer, arthritis, or cardiovascular disease) (National Center for Health Statistics, 2015).

7 Individuals who transition from MA to traditional Medicare during the observation window are included in analysis unless they have a diagnosis of ADRD in their first quarter in traditional Medicare (because their true date of diagnosis may have been earlier). We incorporate a flag signifying an MA quarter to account for incomplete information on the presence or absence of other chronic conditions during these time periods.

8 Rajan et al. (2019) report standardized incidence rates for AD of 0.4%, 3.2%, and 7.5% for the age groups 65–74, 75–84, 85 or over, respectively, using 1994 to 2012 data from 2,794 individuals in the Chicago Health and Aging Population study. Olfson et al. (2021) report incidence rates of dementia for 60 year old men and women (0.8%, 0.7%), 80 year old men and women (3.4%, 3.6%) and 90 year old men and women (8.8%, 9.6%) using Medicare data from 2007–2017.

9 The mean credit score in the entire CCP population is 715. Average credit scores increase with age: the average is 766 for those 65 and older, and 773 for those 75 and older at the end of 2023.

10 For hyperlipidemia, for example, there is a 0.3 point reduction in credit scores a year prior to diagnosis vs. a 5.3 point decrease a year prior to an ADRD diagnosis, and a 0.14 percentage point increase in the probability of payment delinquency a year prior to diagnosis vs. a 1.5 percentage point increase a year prior to ADRD diagnosis. With respect to glaucoma, credit scores are 0.8 points lower a year prior to glaucoma diagnosis vs 5.3 points lower a year prior to ADRD diagnosis, and the probability of a payment delinquency is 0.48 percentage points higher a year prior to glaucoma diagnosis vs 1.5 percentage points higher a year prior to ADRD diagnosis.

## Appendix A. Supplementary data

Supplementary material related to this article can be found online at https://doi.org/10.1016/j.jfineco.2025.104149.
