# Supplementary material for: The financial consequences of undiagnosed memory disorders
Source: J financ econ. Author manuscript; Available in PMC 2025 Dec 16. (PMC12705035; doi:10.1016/j.jfineco.2025.104149)
Supplement: Appendix [file NIHMS2128506-supplement-Appendix.pdf]

## A. Supplementary Appendix

Table A.1: Mean Values of Variables Before and After Propensity Score Weighting

| | Means (Unweighted) | | | Means (Weighted) | | |
|---|---|---|---|---|---|---|
| Variable | Never ADRD | Ever ADRD | Std Diff | Never ADRD | Ever ADRD | Std Diff |
| INDEPENDENT VARIABLES | | | | | | |
| Female | 0.530 | 0.599 | 0.138 | 0.544 | 0.528 | -0.032 |
| Probable spouse/partner (@ age 65) | 0.562 | 0.444 | -0.238 | 0.536 | 0.526 | -0.020 |
| Credit score (@age 65) | 745.4 | 747.4 | 0.024 | 745.6 | 742.1 | -0.042 |
| Household size=2 (@ age 65) | 0.404 | 0.385 | -0.038 | 0.399 | 0.392 | -0.015 |
| Household size=3 (@ age 65) | 0.195 | 0.164 | -0.081 | 0.188 | 0.189 | 0.001 |
| Household size=4 (@ age 65) | 0.089 | 0.073 | -0.058 | 0.085 | 0.087 | 0.005 |
| Household size=5 (@ age 65) | 0.039 | 0.033 | -0.031 | 0.038 | 0.038 | 0.002 |
| Household size=6+ (@ age 65) | 0.079 | 0.096 | 0.063 | 0.083 | 0.084 | 0.007 |
| Black | 0.084 | 0.091 | 0.028 | 0.086 | 0.09 | 0.015 |
| Asian | 0.013 | 0.008 | -0.046 | 0.012 | 0.012 | 0.000 |
| Hispanic | 0.012 | 0.014 | 0.012 | 0.013 | 0.013 | 0.006 |
| Other race | 0.022 | 0.011 | -0.085 | 0.02 | 0.019 | -0.002 |
| Low education tract (@ 65) | 0.426 | 0.445 | 0.037 | 0.431 | 0.439 | 0.017 |
| Low adult smoking county (@ 65) | 0.654 | 0.650 | -0.008 | 0.653 | 0.651 | -0.004 |
| Low adult obesity county (@ 65) | 0.715 | 0.710 | -0.010 | 0.714 | 0.712 | -0.003 |
| Low excessive drinking county(@65) | 0.274 | 0.270 | -0.009 | 0.273 | 0.273 | 0.000 |
| Low air pollution county (@ 65) | 0.359 | 0.358 | -0.002 | 0.359 | 0.358 | -0.001 |
| Low physical inactivity county (@ 65) | 0.765 | 0.758 | -0.017 | 0.763 | 0.761 | -0.004 |
| Hypertension (@ age 65) | 0.177 | 0.311 | 0.317 | 0.208 | 0.216 | 0.018 |
| Diabetes (@ age 65) | 0.087 | 0.133 | 0.146 | 0.099 | 0.107 | 0.026 |
| Depression (@ age 65) | 0.029 | 0.056 | 0.133 | 0.036 | 0.037 | 0.005 |
| Hyperlipidemia (@ age 65) | 0.126 | 0.195 | 0.190 | 0.142 | 0.143 | 0.004 |
| Birth year | 1936.5 | 1928.4 | -0.948 | 1934.8 | 1934.6 | -0.016 |
| OUTCOMES @ AGE 65 | | | | | | |
| Credit score | 745.4 | 747.4 | 0.024 | 745.6 | 742.1 | -0.042 |
| Any delinquency | 0.070 | 0.065 | -0.019 | 0.070 | 0.074 | 0.017 |
| Any mortgage delinquency | 0.022 | 0.019 | -0.020 | 0.022 | 0.022 | 0.003 |
| Delinquent mortgage balance | 2,837.4 | 1,612.4 | -0.044 | 2,665.4 | 2,436.7 | -0.008 |
| Any credit card delinquency | 0.049 | 0.048 | -0.007 | 0.049 | 0.053 | 0.020 |
| Delinquent credit card balance | 298.6 | 228.2 | -0.028 | 289.1 | 285.5 | -0.001 |
| Credit card utilization rate | 0.222 | 0.202 | -0.065 | 0.220 | 0.217 | -0.011 |

Note: Std Diff refers to the standardized difference.

Table A.2: Full Results (Models in Figure 2)

| | **(1) Risk Score** | **(2) Any Delinquency** |
|---|---|---|
| 1 quarter prior to diagnosis | -7.271*** | 0.0223*** |
| | (-27.87) | (18.72) |
| 2 quarters prior to diagnosis | -6.414*** | 0.0191*** |
| | (-24.94) | (15.98) |
| 3 quarters prior to diagnosis | -5.657*** | 0.0163*** |
| | (-22.55) | (13.77) |
| 4 quarters prior to diagnosis | -5.270*** | 0.0148*** |
| | (-21.50) | (12.61) |
| 5 quarters prior to diagnosis | -4.876*** | 0.0133*** |
| | (-19.92) | (11.24) |
| 6 quarters prior to diagnosis | -4.466*** | 0.0126*** |
| | (-18.11) | (10.42) |
| 7 quarters prior to diagnosis | -4.064*** | 0.0111*** |
| | (-16.37) | (9.31) |
| 8 quarters prior to diagnosis | -3.629*** | 0.0104*** |
| | (-14.69) | (8.76) |
| 9 quarters prior to diagnosis | -3.273*** | 0.00880*** |
| | (-13.39) | (7.50) |
| 10 quarters prior to diagnosis | -3.051*** | 0.00814*** |
| | (-12.62) | (6.87) |
| 11 quarters prior to diagnosis | -2.798*** | 0.00724*** |
| | (-11.88) | (6.17) |
| 12 quarters prior to diagnosis | -2.561*** | 0.00692*** |
| | (-10.96) | (5.80) |
| 13 quarters prior to diagnosis | -2.456*** | 0.00647*** |
| | (-10.54) | (5.53) |
| 14 quarters prior to diagnosis | -2.300*** | 0.00656*** |
| | (-9.94) | (5.54) |
| 15 quarters prior to diagnosis | -2.159*** | 0.00703*** |
| | (-9.37) | (5.82) |
| 16 quarters prior to diagnosis | -1.940*** | 0.00570*** |
| | (-8.52) | (4.76) |
| 17 quarters prior to diagnosis | -1.866*** | 0.00598*** |
| | (-8.33) | (5.05) |
| 18 quarters prior to diagnosis | -1.666*** | 0.00593*** |
| | (-7.58) | (5.04) |
| 19 quarters prior to diagnosis | -1.690*** | 0.00638*** |
| | (-7.66) | (5.40) |
| 20 quarters prior to diagnosis | -1.528*** | 0.00493*** |
| | (-6.91) | (4.19) |
| 21 quarters prior to diagnosis | -1.380*** | 0.00487*** |
| | (-6.51) | (4.34) |
| 22 quarters prior to diagnosis | -0.998*** | 0.00306** |
| | (-4.96) | (2.82) |
| 23 quarters prior to diagnosis | -0.816*** | 0.00287** |
| | (-4.21) | (2.71) |
| 24 quarters prior to diagnosis | -0.680*** | 0.00276** |
| | (-3.59) | (2.67) |
| 25 quarters prior to diagnosis | -0.606** | 0.00267* |
| | (-3.22) | (2.54) |
| 26 quarters prior to diagnosis | -0.575** | 0.00406*** |
| | (-3.05) | (3.80) |
| 27 quarters prior to diagnosis | -0.290 | 0.00243* |
| | (-1.61) | (2.34) |
| 28 quarters prior to diagnosis | -0.232 | 0.00211* |
| | (-1.34) | (2.13) |
| 0 quarters after diagnosis | -8.478*** | 0.0273*** |
| | (-32.80) | (22.95) |

| | (1) Risk Score | (2) Any Delinquency |
|---|---|---|
| 1 quarter after diagnosis | -9.904*** | 0.0305*** |
| | (-39.61) | (26.63) |
| 2 quarters after diagnosis | -10.45*** | 0.0307*** |
| | (-42.89) | (26.58) |
| 3 quarters after diagnosis | -10.65*** | 0.0305*** |
| | (-44.12) | (27.05) |
| 4 quarters after diagnosis | -10.86*** | 0.0297*** |
| | (-44.34) | (25.87) |
| 5 quarters after diagnosis | -10.92*** | 0.0295*** |
| | (-43.96) | (25.28) |
| 6 quarters after diagnosis | -10.86*** | 0.0288*** |
| | (-43.02) | (24.19) |
| 7 quarters after diagnosis | -10.94*** | 0.0283*** |
| | (-42.53) | (23.45) |
| 8 quarters after diagnosis | -11.02*** | 0.0278*** |
| | (-41.91) | (22.53) |
| 9 quarters after diagnosis | -10.94*** | 0.0268*** |
| | (-40.78) | (21.28) |
| 10 quarters after diagnosis | -10.79*** | 0.0257*** |
| | (-39.58) | (20.00) |
| 11 quarters after diagnosis | -10.80*** | 0.0245*** |
| | (-39.07) | (18.80) |
| 12 quarters after diagnosis | -10.78*** | 0.0241*** |
| | (-38.20) | (18.21) |
| 13 quarters after diagnosis | -10.88*** | 0.0240*** |
| | (-37.88) | (17.70) |
| 14 quarters after diagnosis | -10.88*** | 0.0236*** |
| | (-37.08) | (16.98) |
| 15 quarters after diagnosis | -10.95*** | 0.0228*** |
| | (-36.39) | (16.07) |
| 16 quarters after diagnosis | -10.90*** | 0.0213*** |
| | (-35.63) | (14.80) |
| 17 quarters after diagnosis | -10.82*** | 0.0207*** |
| | (-34.87) | (14.22) |
| 18 quarters after diagnosis | -10.86*** | 0.0200*** |
| | (-34.10) | (13.44) |
| 19 quarters after diagnosis | -10.87*** | 0.0194*** |
| | (-33.55) | (12.78) |
| 20 quarters after diagnosis | -11.16*** | 0.0198*** |
| | (-33.70) | (12.76) |
| 21 quarters after diagnosis | -11.26*** | 0.0192*** |
| | (-33.21) | (12.14) |
| 22 quarters after diagnosis | -11.22*** | 0.0184*** |
| | (-32.36) | (11.50) |
| 23 quarters after diagnosis | -11.25*** | 0.0170*** |
| | (-31.86) | (10.45) |
| 24 quarters after diagnosis | -11.25*** | 0.0152*** |
| | (-31.19) | (9.15) |
| 25 quarters after diagnosis | -10.90*** | 0.0147*** |
| | (-29.44) | (8.61) |
| 26 quarters after diagnosis | -10.86*** | 0.0147*** |
| | (-28.74) | (8.45) |
| 27 quarters after diagnosis | -10.68*** | 0.0127*** |
| | (-27.64) | (7.20) |
| 28 quarters after diagnosis | -10.59*** | 0.0112*** |
| | (-27.07) | (6.23) |
| 29 + quarters after diagnosis | -10.94*** | 0.0100*** |
| | (-26.81) | (5.75) |

| | (1) Risk Score | (2) Any Delinquency |
|---|---|---|
| Spouse | 2.063*** | -0.00417*** |
| | (15.77) | (-6.68) |
| Household size=2 | -0.849*** | 0.00341*** |
| | (-6.71) | (5.63) |
| Household size=3 | -2.058*** | 0.00570*** |
| | (-13.13) | (7.89) |
| Household size=4 | -3.478*** | 0.00917*** |
| | (-18.91) | (10.55) |
| Household size=5 | -4.508*** | 0.0125*** |
| | (-20.77) | (11.94) |
| Household size=6 or more | -4.202*** | 0.0117*** |
| | (-20.52) | (10.95) |
| Number of chronic conditions | -0.661*** | 0.00194*** |
| | (-13.06) | (8.03) |
| MA quarter | 0.0216 | 0.00424*** |
| | (0.14) | (5.00) |
| AMI | 0.111 | 0.00134 |
| | (0.51) | (1.16) |
| Anemia | 0.547*** | -0.00156*** |
| | (6.60) | (-3.94) |
| Asthma | 0.862*** | -0.00167* |
| | (5.12) | (-2.14) |
| Atrial fib | 1.020*** | -0.00174** |
| | (7.47) | (-2.76) |
| Breast cancer | 0.583** | -0.000986 |
| | (3.01) | (-1.18) |
| Colorectal cancer | 0.999*** | -0.00237 |
| | (3.55) | (-1.49) |
| Endometrial cancer | 0.989 | -0.00190 |
| | (1.27) | (-0.46) |
| Lung cancer | 0.0820 | 0.00431* |
| | (0.23) | (2.54) |
| Prostate cancer | 1.150*** | -0.00382*** |
| | (5.58) | (-3.99) |
| Cataracts | 1.063*** | -0.00400*** |
| | (15.92) | (-13.15) |
| CHF | -0.0677 | -0.000355 |
| | (-0.58) | (-0.63) |
| Chronic kidney disease | 0.299* | -0.00185** |
| | (2.42) | (-2.98) |
| COPD | 0.889*** | -0.00361*** |
| | (7.13) | (-5.88) |
| Diabetes | 1.098*** | -0.00350*** |
| | (8.31) | (-5.66) |
| Glaucoma | 2.051*** | -0.00524*** |
| | (18.88) | (-10.84) |
| Hip or pelvis fracture | -1.398*** | 0.00671*** |
| | (-6.48) | (6.48) |
| Hyperplasia | 0.841*** | -0.00249*** |
| | (7.00) | (-4.36) |
| Hypertension | 1.019*** | -0.00265*** |
| | (12.38) | (-6.73) |
| Hyperthyroidism | 0.304** | -0.000172 |
| | (2.61) | (-0.32) |
| Ischemic heart disease | 0.616*** | -0.00270*** |
| | (6.55) | (-6.13) |
| Osteoporosis | 0.780*** | -0.00208*** |
| | (7.60) | (-4.41) |

| | (1) Risk Score | (2) Any Delinquency |
|---|---|---|
| Rheumatoid/osteoarthritis | 0.707*** | -0.00134*** |
| | (8.45) | (-3.31) |
| Stroke/TIA | -0.145 | 0.00219** |
| | (-1.02) | (3.22) |
| Constant | 720.6*** | 0.0717*** |
| | (300.95) | (5.70) |
| N | 91,230,055 | 91,230,055 |

Notes: *p<0.05,** p<0.01,*** p<0.001. t statistics in parentheses. Coefficient estimates for state-time and age dummies not shown.

Table A.3: Propensity Score Model

| | **(1) Ever ADRD** |
|---|---|
| Female | 0.191*** |
| | (0.00417) |
| Spouse @65 | -0.0484*** |
| | (0.00515) |
| Credit score @ 65 | -0.00126*** |
| | (0.0000282) |
| Household size=2 (@65) | -0.0235*** |
| | (0.00632) |
| Household size=3 (@65) | -0.0318*** |
| | (0.00730) |
| Household size=4 (@65) | -0.0300** |
| | (0.00919) |
| Household size=5 (@65) | -0.0253* |
| | (0.0123) |
| Household size=6 or more (@65) | 0.0145 |
| | (0.00815) |
| Black | 0.0454*** |
| | (0.00792) |
| Asian | -0.156*** |
| | (0.0211) |
| Hispanic | 0.0519** |
| | (0.0183) |
| Other race | -0.166*** |
| | (0.0180) |
| Below median percentage of adults 25+ with HS or more | 0.0150*** |
| (Census tract, @65)^ | (0.00446) |
| Below median adult smoking rate (county, @65)^ | 0.0122* |
| | (0.00554) |
| Below median adult obesity rate (county, @65)^ | -0.0245*** |
| | (0.00649) |
| Below median excessive drinking rate (county, @65)^ | 0.00826 |
| | (0.00595) |
| Below median air pollution rate (county, @65)^ | -0.00271 |
| | (0.00549) |
| Below median physical inactivity rate (county, @65)^ | -0.0349*** |
| | (0.00669) |
| Hypertension (@65) | 0.138*** |
| | (0.00529) |
| Diabetes (@65) | 0.139*** |
| | (0.00686) |
| Depression (@65) | 0.703*** |
| | (0.0105) |
| Hyperlipidemia (@65) | 0.122*** |
| | (0.00590) |
| birthyear 1895-1899 | 3.839*** |
| | (0.862) |
| birthyear 1900-1904 | 4.573*** |
| | (0.159) |
| birthyear 1905-1909 | 4.612*** |
| | (0.136) |
| birthyear 1910-1914 | 4.711*** |
| | (0.134) |
| birthyear 1915-1919 | 4.654*** |
| | (0.134) |
| birthyear 1920-1924 | 4.467*** |
| | (0.133) |
| birthyear 1925-1929 | 4.073*** |
| | (0.133) |

| | (1) Ever ADRD |
|---|---|
| birthyear 1930-1934 | 3.542*** |
| | (0.133) |
| birthyear 1935-1939 | 3.116*** |
| | (0.133) |
| birthyear 1940-1944 | 2.322*** |
| | (0.134) |
| birthyear 1945-1949 | 1.751*** |
| | (0.139) |
| birthyear 1950 or later | 0 |
| | (.) |
| 1.states | 0 |
| | (.) |
| 3.states | -0.268*** |
| | (0.0230) |
| 4.states | 0.194*** |
| | (0.0253) |
| 5.states | -0.152*** |
| | (0.0184) |
| 6.states | -0.187*** |
| | (0.0256) |
| 7.states | -0.0402 |
| | (0.0233) |
| 8.states | 0.112** |
| | (0.0363) |
| 9.states | 0.226*** |
| | (0.0452) |
| 10.states | -0.0256 |
| | (0.0185) |
| 11.states | -0.0323 |
| | (0.0205) |
| 13.states | -0.373*** |
| | (0.0372) |
| 14.states | -0.0278 |
| | (0.0193) |
| 15.states | -0.137*** |
| | (0.0206) |
| 16.states | -0.237*** |
| | (0.0252) |
| 17.states | -0.0229 |
| | (0.0258) |
| 18.states | -0.0837*** |
| | (0.0232) |
| 19.states | -0.0617** |
| | (0.0228) |
| 20.states | -0.175*** |
| | (0.0335) |
| 21.states | 0.0383 |
| | (0.0209) |
| 22.states | -0.0235 |
| | (0.0211) |
| 23.states | -0.0435* |
| | (0.0193) |
| 24.states | -0.639*** |
| | (0.0256) |
| 25.states | 0.0778** |
| | (0.0252) |
| 26.states | -0.0737*** |
| | (0.0220) |

| | (1) Ever ADRD |
|---|---|
| 27.states | -0.247*** |
| | (0.0391) |
| 28.states | -0.156*** |
| | (0.0309) |
| 29.states | -0.153*** |
| | (0.0305) |
| 30.states | 0.0274 |
| | (0.0330) |
| 31.states | 0.0610** |
| | (0.0195) |
| 32.states | -0.165*** |
| | (0.0324) |
| 33.states | -0.193*** |
| | (0.0185) |
| 34.states | -0.101*** |
| | (0.0198) |
| 35.states | -0.156*** |
| | (0.0458) |
| 36.states | -0.249*** |
| | (0.0190) |
| 37.states | 0.0419 |
| | (0.0245) |
| 38.states | -0.460*** |
| | (0.0269) |
| 39.states | -0.254*** |
| | (0.0189) |
| 40.states | -0.234*** |
| | (0.0383) |
| 41.states | -0.0125 |
| | (0.0222) |
| 42.states | -0.309*** |
| | (0.0436) |
| 43.states | -0.114*** |
| | (0.0212) |
| 44.states | 0.0821*** |
| | (0.0187) |
| 45.states | -0.343*** |
| | (0.0320) |
| 46.states | -0.197*** |
| | (0.0480) |
| 47.states | 0.0339 |
| | (0.0205) |
| 48.states | -0.185*** |
| | (0.0220) |
| 49.states | -0.196*** |
| | (0.0305) |
| 50.states | -0.367*** |
| | (0.0227) |
| 51.states | -0.0622 |
| | (0.0505) |
| 2000.year | 0 |
| | (.) |
| 2001.year | -0.0161 |
| | (0.0130) |
| 2002.year | -0.134*** |
| | (0.0129) |
| 2003.year | -0.267*** |
| | (0.0131) |

| | (1) Ever ADRD |
|---|---|
| 2004.year | -0.406*** |
| | (0.0139) |
| 2005.year | 0.191*** |
| | (0.0207) |
| 2006.year | 0.0927*** |
| | (0.0210) |
| 2007.year | -0.0382 |
| | (0.0212) |
| 2008.year | -0.160*** |
| | (0.0220) |
| 2009.year | -0.304*** |
| | (0.0218) |
| 2010.year | 0.0858 |
| | (0.0443) |
| 2011.year | -0.125** |
| | (0.0453) |
| 2017.year | -0.541*** |
| | (0.121) |
| Constant | -3.706*** |
| | (0.136) |
| N | 1670313 |

Notes: Variables measured as of age 65 or the earliest age of observation. Ĉounty level data from County Health Rankings (as derived from BRFSS, CDC WONDER, and the CDC Diabetes Interactive Atlas). Census tract level information on education from the American Community Survey. Variables are dichotomous indicators for whether the area has a rate lower than the median. Standard errors in parentheses.
*p<0.05, **p<0.01, *** p<0.001.

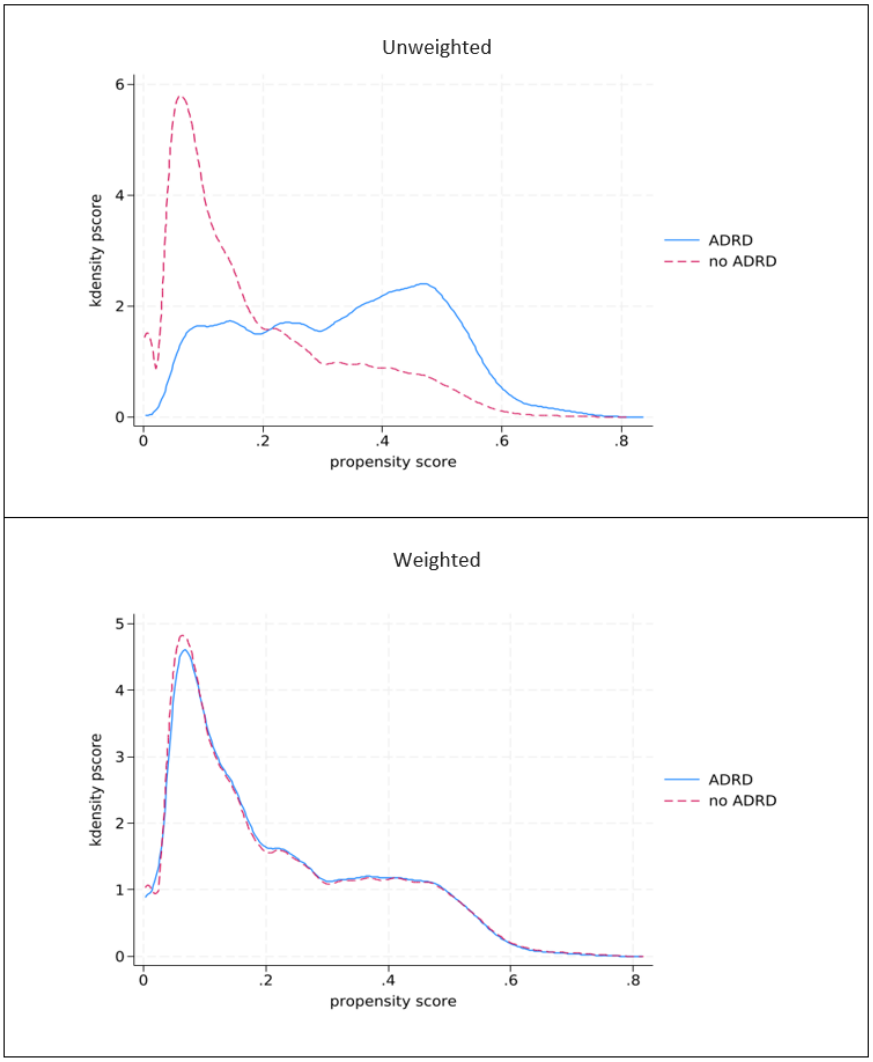


Figure A.1: Propensity Score Distribution for Treatment and Comparison Groups: Unweighted and Weighted

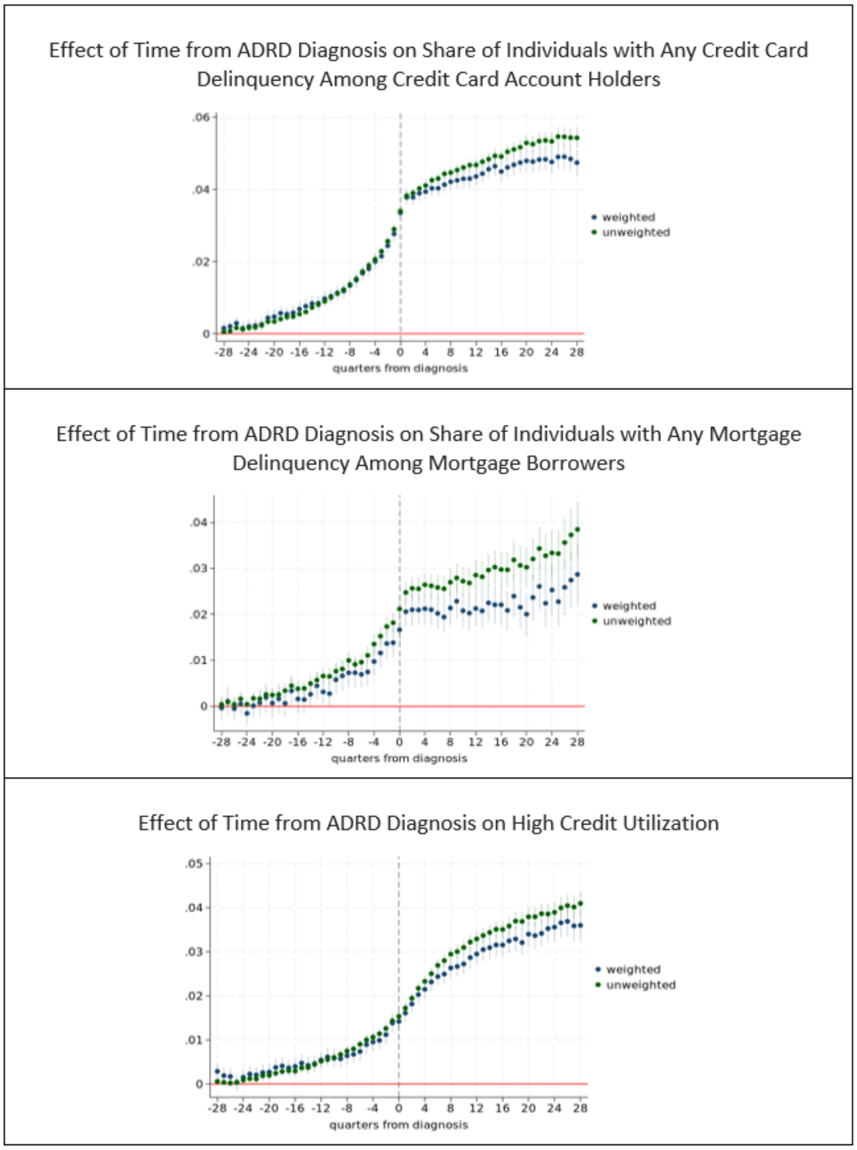


Figure A.2: Effect of Time from ADRD Diagnosis on Additional Outcomes: Unweighted vs. Propensity Score Weighted

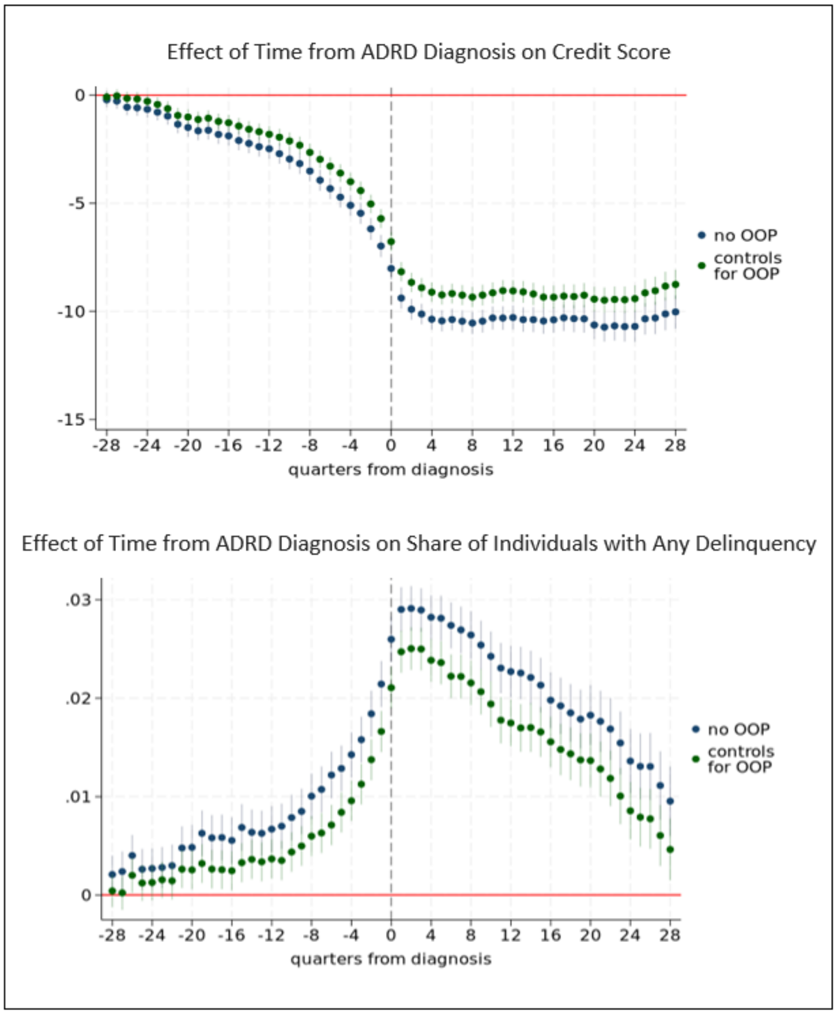


Figure A.3: Effect of Time from ADRD Diagnosis on Credit Score and Share of Individuals with Any Delinquency: Including Out of Pocket Costs

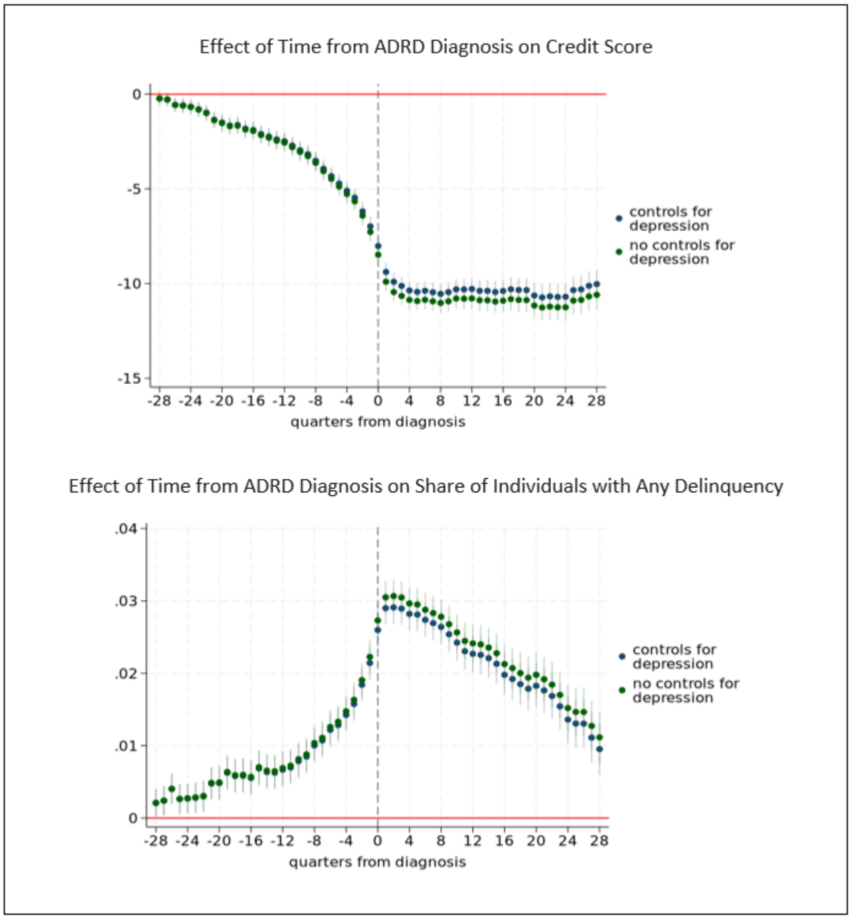


Figure A.4: Effect of Time from ADRD Diagnosis on Credit Score and Share of Individuals with Any Delinquency: Including Quarterly Depression Indicators

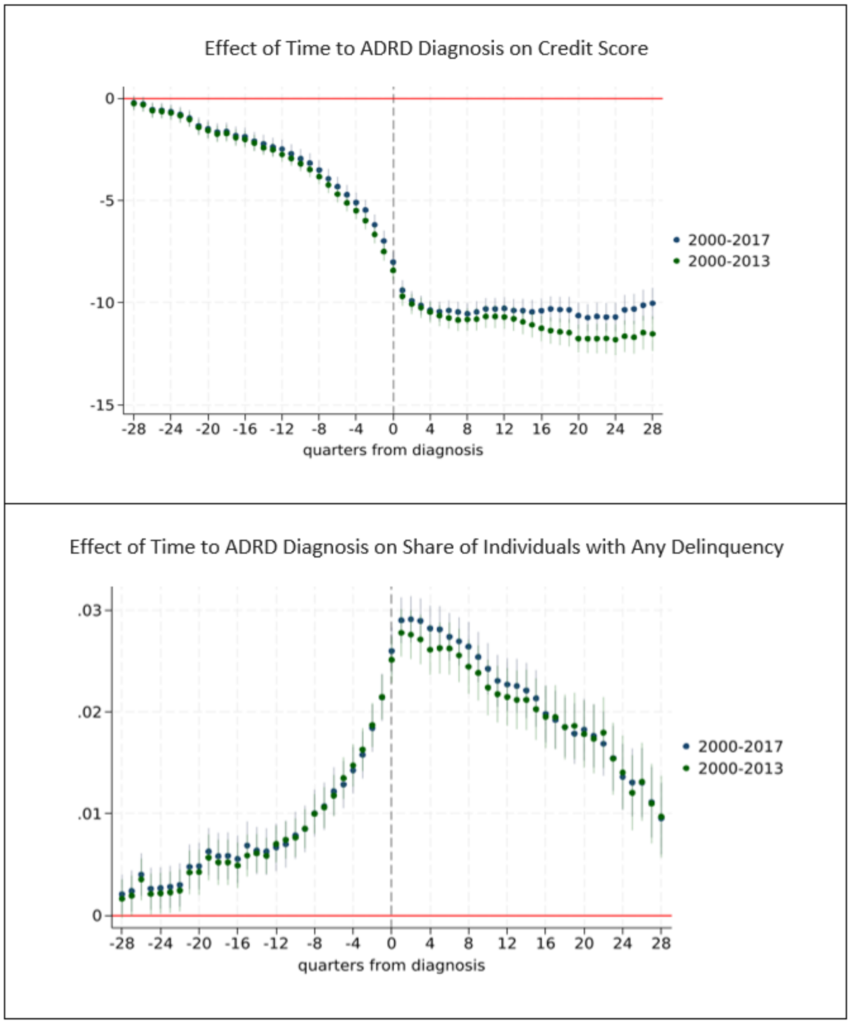


Figure A.5: Effect of Time from ADRD Diagnosis on Credit Score and Share of Individuals with Any Delinquency: Endpoint of Timeframe Limited to 2013

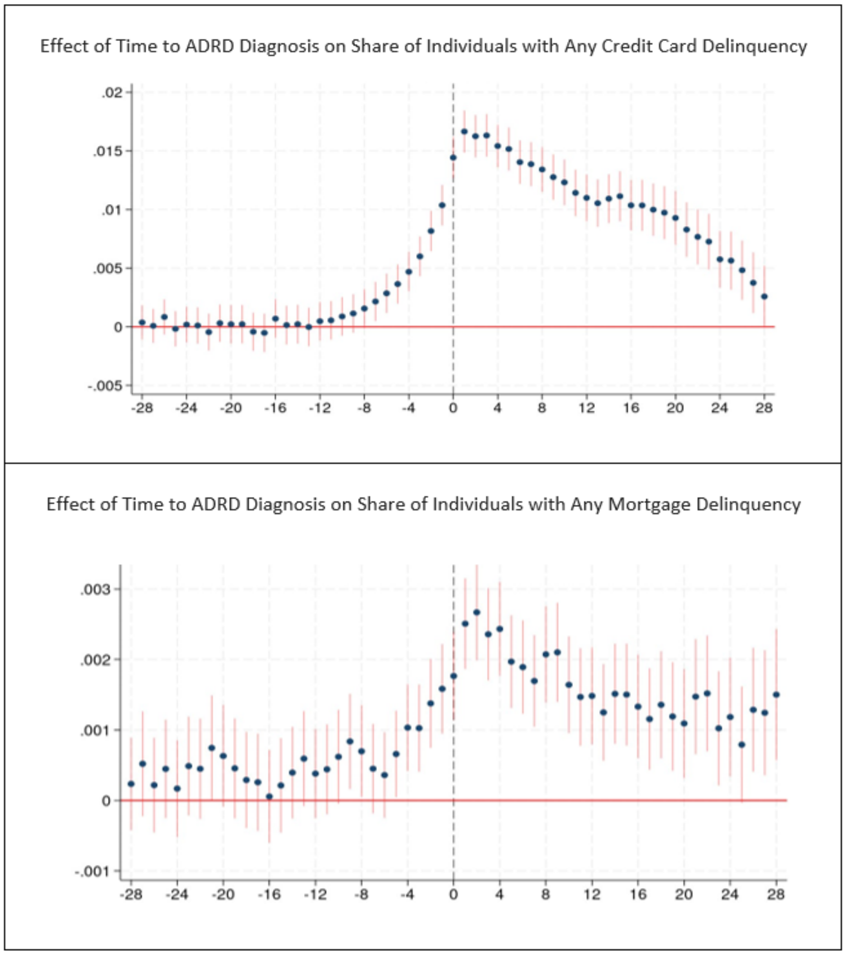


Figure A.6: Effect of Time from ADRD Diagnosis on Share of Individuals with Any Credit Card Delinquency and Share of Individuals with Any Mortgage Delinquency Among All Individuals (Not Limited to Account Holders)

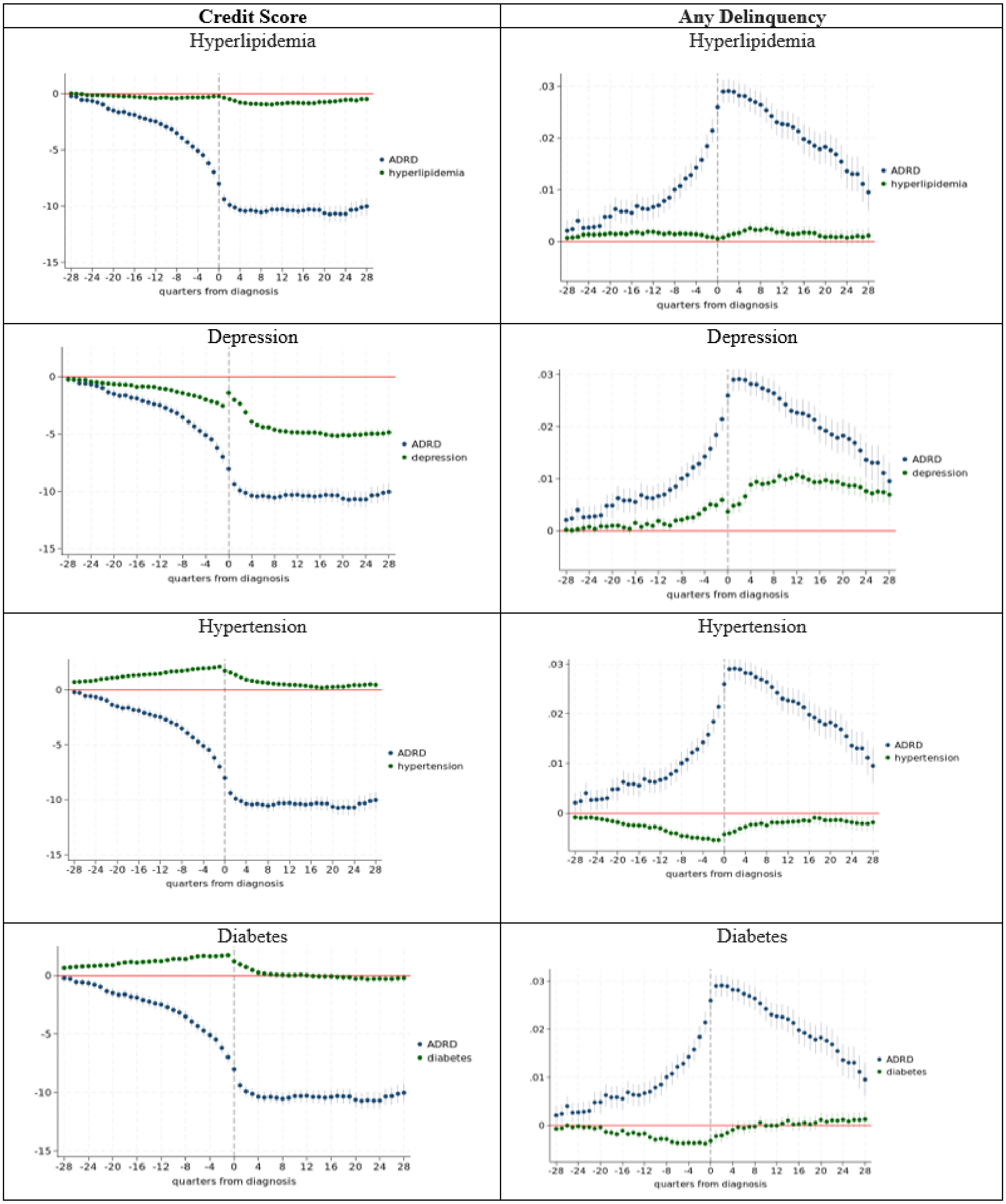


Figure A.7: Effect of Time to Diagnosis from Additional Placebo Conditions on Credit Score and Share of Individuals with Any Delinquency

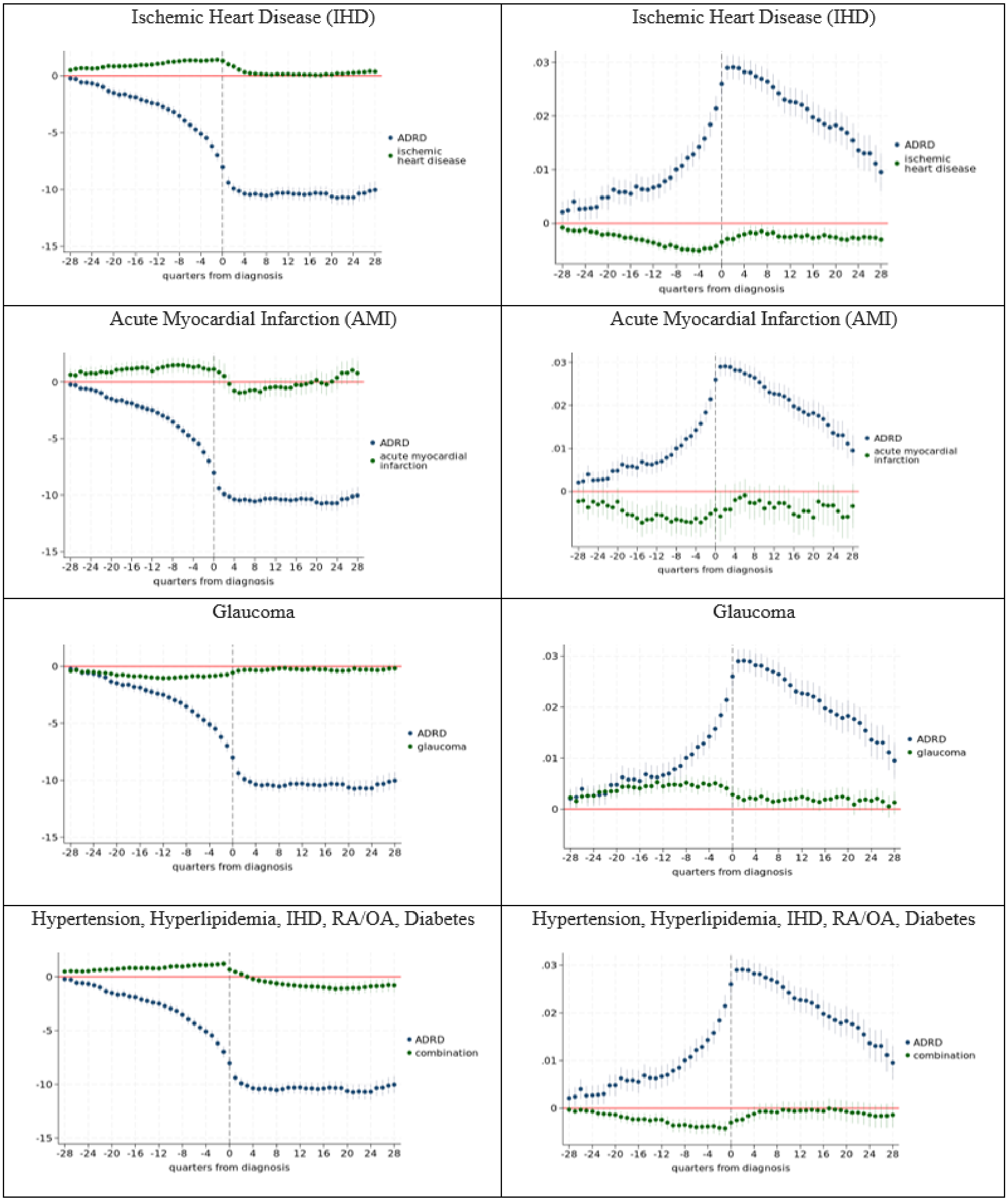


Note: Results are for the sample of individuals never diagnosed with ADRD.

Figure A.7: (Continued) Effect of Time to Diagnosis from Additional Placebo Conditions on Credit Score and Share of Individuals with Any Delinquency
