# Supplementary material for: The financial consequences of undiagnosed memory disorders
Source: J financ econ. Author manuscript; Available in PMC 2025 Dec 16. (PMC12705035; doi:10.1016/j.jfineco.2025.104149)
Supplement: Data Replication Files [file NIHMS2128506-supplement-Data_Replication_Files.zip › replication_final/data_raw/geo/health_rank/DataDictionary_2011.pdf]

County Health Rankings 2011 Data Dictionary

Variable Name	Measure
statecode	State FIPS Code
countycode	County FIPS Code
fipscode	5-digit FIPS Code
state	State Abbreviation
county	Name
year	Release Year
county_ranked	County Ranked (Yes=1/No=0)
v001_rawvalue	Premature death raw value
v001_numerator	Premature death numerator
v001_denominator	Premature death denominator
v001_cilow	Premature death CI low
v001_cihigh	Premature death CI high
v002_rawvalue	Poor or fair health raw value
v002_numerator	Poor or fair health numerator
v002_denominator	Poor or fair health denominator
v002_cilow	Poor or fair health CI low
v002_cihigh	Poor or fair health CI high
v036_rawvalue	Poor physical health days raw value
v036_numerator	Poor physical health days numerator
v036_denominator	Poor physical health days denominator
v036_cilow	Poor physical health days CI low
v036_cihigh	Poor physical health days CI high
v042_rawvalue	Poor mental health days raw value
v042_numerator	Poor mental health days numerator
v042_denominator	Poor mental health days denominator
v042_cilow	Poor mental health days CI low
v042_cihigh	Poor mental health days CI high
v037_rawvalue	Low birthweight raw value
v037_numerator	Low birthweight numerator
v037_denominator	Low birthweight denominator
v037_cilow	Low birthweight CI low

Measures are listed in order of appearance on the county snapshots and analytic datasets.
Measures with the description "CI low" or "CI high" indicate the lower and upper limits of the confidence interval.

Variable Name	Measure
v037_cihigh	Low birthweight CI high
v009_rawvalue	Adult smoking raw value
v009_numerator	Adult smoking numerator
v009_denominator	Adult smoking denominator
v009_cilow	Adult smoking CI low
v009_cihigh	Adult smoking CI high
v011_rawvalue	Adult obesity raw value
v011_numerator	Adult obesity numerator
v011_denominator	Adult obesity denominator
v011_cilow	Adult obesity CI low
v011_cihigh	Adult obesity CI high
v049_rawvalue	Excessive drinking raw value
v049_numerator	Excessive drinking numerator
v049_denominator	Excessive drinking denominator
v049_cilow	Excessive drinking CI low
v049_cihigh	Excessive drinking CI high
v039_rawvalue	Motor vehicle crash deaths raw value
v039_numerator	Motor vehicle crash deaths numerator
v039_denominator	Motor vehicle crash deaths denominator
v039_cilow	Motor vehicle crash deaths CI low
v039_cihigh	Motor vehicle crash deaths CI high
v045_rawvalue	Sexually transmitted infections raw value
v045_numerator	Sexually transmitted infections numerator
v045_denominator	Sexually transmitted infections denominator
v045_cilow	Sexually transmitted infections CI low
v045_cihigh	Sexually transmitted infections CI high
v014_rawvalue	Teen births raw value
v014_numerator	Teen births numerator
v014_denominator	Teen births denominator
v014_cilow	Teen births CI low
v014_cihigh	Teen births CI high

Variable Name	Measure
v003_rawvalue	Uninsured adults raw value
v003_numerator	Uninsured adults numerator
v003_denominator	Uninsured adults denominator
v003_cilow	Uninsured adults CI low
v003_cihigh	Uninsured adults CI high
v004_rawvalue	Primary care physicians raw value
v004_numerator	Primary care physicians numerator
v004_denominator	Primary care physicians denominator
v004_cilow	Primary care physicians CI low
v004_cihigh	Primary care physicians CI high
v004_other_data_1	Ratio of population to primary care physicians
v005_rawvalue	Preventable hospital stays raw value
v005_numerator	Preventable hospital stays numerator
v005_denominator	Preventable hospital stays denominator
v005_cilow	Preventable hospital stays CI low
v005_cihigh	Preventable hospital stays CI high
v007_rawvalue	Diabetes monitoring raw value
v007_numerator	Diabetes monitoring numerator
v007_denominator	Diabetes monitoring denominator
v007_cilow	Diabetes monitoring CI low
v007_cihigh	Diabetes monitoring CI high
v050_rawvalue	Mammography screening raw value
v050_numerator	Mammography screening numerator
v050_denominator	Mammography screening denominator
v050_cilow	Mammography screening CI low
v050_cihigh	Mammography screening CI high
v021_rawvalue	High school graduation raw value
v021_numerator	High school graduation numerator
v021_denominator	High school graduation denominator
v021_cilow	High school graduation CI low
v021_cihigh	High school graduation CI high

Measures are listed in order of appearance on the county snapshots and analytic datasets.
Measures with the description "CI low" or "CI high" indicate the lower and upper limits of the confidence interval.

Variable Name	Measure
v069_rawvalue	Some college raw value
v069_numerator	Some college numerator
v069_denominator	Some college denominator
v069_cilow	Some college CI low
v069_cihigh	Some college CI high
v023_rawvalue	Unemployment raw value
v023_numerator	Unemployment numerator
v023_denominator	Unemployment denominator
v023_cilow	Unemployment CI low
v023_cihigh	Unemployment CI high
v024_rawvalue	Children in poverty raw value
v024_numerator	Children in poverty numerator
v024_denominator	Children in poverty denominator
v024_cilow	Children in poverty CI low
v024_cihigh	Children in poverty CI high
v040_rawvalue	Inadequate social support raw value
v040_numerator	Inadequate social support numerator
v040_denominator	Inadequate social support denominator
v040_cilow	Inadequate social support CI low
v040_cihigh	Inadequate social support CI high
v082_rawvalue	Children in single-parent households raw value
v082_numerator	Children in single-parent households numerator
v082_denominator	Children in single-parent households denominator
v082_cilow	Children in single-parent households CI low
v082_cihigh	Children in single-parent households CI high
v043_rawvalue	Violent crime raw value
v043_numerator	Violent crime numerator
v043_denominator	Violent crime denominator
v043_cilow	Violent crime CI low
v043_cihigh	Violent crime CI high
v015_rawvalue	Homicides raw value

Variable Name	Measure
v015_numerator	Homicides numerator
v015_denominator	Homicides denominator
v015_cilow	Homicides CI low
v015_cihigh	Homicides CI high
v046_rawvalue	Air pollution-particulate matter days raw value
v046_numerator	Air pollution-particulate matter days numerator
v046_denominator	Air pollution-particulate matter days denominator
v046_cilow	Air pollution-particulate matter days CI low
v046_cihigh	Air pollution-particulate matter days CI high
v029_rawvalue	Air pollution-ozone days raw value
v029_numerator	Air pollution-ozone days numerator
v029_denominator	Air pollution-ozone days denominator
v029_cilow	Air pollution-ozone days CI low
v029_cihigh	Air pollution-ozone days CI high
v030_rawvalue	Access to healthy foods raw value
v030_numerator	Access to healthy foods numerator
v030_denominator	Access to healthy foods denominator
v030_cilow	Access to healthy foods CI low
v030_cihigh	Access to healthy foods CI high
v068_rawvalue	Access to recreational facilities raw value
v068_numerator	Access to recreational facilities numerator
v068_denominator	Access to recreational facilities denominator
v068_cilow	Access to recreational facilities CI low
v068_cihigh	Access to recreational facilities CI high
v060_rawvalue	Diabetes prevalence raw value
v060_numerator	Diabetes prevalence numerator
v060_denominator	Diabetes prevalence denominator
v060_cilow	Diabetes prevalence CI low
v060_cihigh	Diabetes prevalence CI high
v061_rawvalue	HIV prevalence raw value
v061_numerator	HIV prevalence numerator

Variable Name	Measure
v061_denominator	HIV prevalence denominator
v061_cilow	HIV prevalence CI low
v061_cihigh	HIV prevalence CI high
v070_rawvalue	Physical inactivity raw value
v070_numerator	Physical inactivity numerator
v070_denominator	Physical inactivity denominator
v070_cilow	Physical inactivity CI low
v070_cihigh	Physical inactivity CI high
v012_rawvalue	Binge drinking raw value
v012_numerator	Binge drinking numerator
v012_denominator	Binge drinking denominator
v012_cilow	Binge drinking CI low
v012_cihigh	Binge drinking CI high
v062_rawvalue	Mental health providers raw value
v062_numerator	Mental health providers numerator
v062_denominator	Mental health providers denominator
v062_cilow	Mental health providers CI low
v062_cihigh	Mental health providers CI high
v062_other_data_1	Ratio of population to mental health providers
v066_rawvalue	Illiteracy raw value
v066_numerator	Illiteracy numerator
v066_denominator	Illiteracy denominator
v066_cilow	Illiteracy CI low
v066_cihigh	Illiteracy CI high
v063_rawvalue	Median household income raw value
v063_numerator	Median household income numerator
v063_denominator	Median household income denominator
v063_cilow	Median household income CI low
v063_cihigh	Median household income CI high
v064_rawvalue	High housing costs raw value
v064_numerator	High housing costs numerator

Variable Name	Measure
v064_denominator	High housing costs denominator
v064_cilow	High housing costs CI low
v064_cihigh	High housing costs CI high
v065_rawvalue	Children eligible for free lunch raw value
v065_numerator	Children eligible for free lunch numerator
v065_denominator	Children eligible for free lunch denominator
v065_cilow	Children eligible for free lunch CI low
v065_cihigh	Children eligible for free lunch CI high
v041_rawvalue	Liquor store density raw value
v041_numerator	Liquor store density numerator
v041_denominator	Liquor store density denominator
v041_cilow	Liquor store density CI low
v041_cihigh	Liquor store density CI high
v067_rawvalue	Driving alone to work raw value
v067_numerator	Driving alone to work numerator
v067_denominator	Driving alone to work denominator
v067_cilow	Driving alone to work CI low
v067_cihigh	Driving alone to work CI high
v051_rawvalue	Population raw value
v051_numerator	Population numerator
v051_denominator	Population denominator
v051_cilow	Population CI low
v051_cihigh	Population CI high
v052_rawvalue	% below 18 years of age raw value
v052_numerator	% below 18 years of age numerator
v052_denominator	% below 18 years of age denominator
v052_cilow	% below 18 years of age CI low
v052_cihigh	% below 18 years of age CI high
v053_rawvalue	% 65 and older raw value
v053_numerator	% 65 and older numerator
v053_denominator	% 65 and older denominator

Variable Name	Measure
v053_cilow	% 65 and older CI low
v053_cihigh	% 65 and older CI high
v054_rawvalue	% Non-Hispanic African American raw value
v054_numerator	% Non-Hispanic African American numerator
v054_denominator	% Non-Hispanic African American denominator
v054_cilow	% Non-Hispanic African American CI low
v054_cihigh	% Non-Hispanic African American CI high
v055_rawvalue	% American Indian and Alaskan Native raw value
v055_numerator	% American Indian and Alaskan Native numerator
v055_denominator	% American Indian and Alaskan Native denominator
v055_cilow	% American Indian and Alaskan Native CI low
v055_cihigh	% American Indian and Alaskan Native CI high
v081_rawvalue	% Asian raw value
v081_numerator	% Asian numerator
v081_denominator	% Asian denominator
v081_cilow	% Asian CI low
v081_cihigh	% Asian CI high
v080_rawvalue	% Native Hawaiian/Other Pacific Islander raw value
v080_numerator	% Native Hawaiian/Other Pacific Islander numerator
v080_denominator	% Native Hawaiian/Other Pacific Islander denominator
v080_cilow	% Native Hawaiian/Other Pacific Islander CI low
v080_cihigh	% Native Hawaiian/Other Pacific Islander CI high
v056_rawvalue	% Hispanic raw value
v056_numerator	% Hispanic numerator
v056_denominator	% Hispanic denominator
v056_cilow	% Hispanic CI low
v056_cihigh	% Hispanic CI high
v059_rawvalue	% not proficient in English raw value
v059_numerator	% not proficient in English numerator
v059_denominator	% not proficient in English denominator
v059_cilow	% not proficient in English CI low

Variable Name	Measure
v059_cihigh	% not proficient in English CI high
v057_rawvalue	% Females raw value
v057_numerator	% Females numerator
v057_denominator	% Females denominator
v057_cilow	% Females CI low
v057_cihigh	% Females CI high
v058_rawvalue	% Rural raw value
v058_numerator	% Rural numerator
v058_denominator	% Rural denominator
v058_cilow	% Rural CI low
v058_cihigh	% Rural CI high
v091_rawvalue	Fair or poor health (NY) raw value
v091_numerator	Fair or poor health (NY) numerator
v091_denominator	Fair or poor health (NY) denominator
v091_cilow	Fair or poor health (NY) CI low
v091_cihigh	Fair or poor health (NY) CI high
v010_rawvalue	Smoking during pregnancy raw value
v010_numerator	Smoking during pregnancy numerator
v010_denominator	Smoking during pregnancy denominator
v010_cilow	Smoking during pregnancy CI low
v010_cihigh	Smoking during pregnancy CI high
v074_rawvalue	Adult smoking (FL) raw value
v074_numerator	Adult smoking (FL) numerator
v074_denominator	Adult smoking (FL) denominator
v074_cilow	Adult smoking (FL) CI low
v074_cihigh	Adult smoking (FL) CI high
v095_rawvalue	Adult smoking (NY) raw value
v095_numerator	Adult smoking (NY) numerator
v095_denominator	Adult smoking (NY) denominator
v095_cilow	Adult smoking (NY) CI low
v095_cihigh	Adult smoking (NY) CI high

Variable Name	Measure
v075_rawvalue	Overweight or obese adults (FL) raw value
v075_numerator	Overweight or obese adults (FL) numerator
v075_denominator	Overweight or obese adults (FL) denominator
v075_cilow	Overweight or obese adults (FL) CI low
v075_cihigh	Overweight or obese adults (FL) CI high
v076_rawvalue	Fruit and vegetable consumption (FL) raw value
v076_numerator	Fruit and vegetable consumption (FL) numerator
v076_denominator	Fruit and vegetable consumption (FL) denominator
v076_cilow	Fruit and vegetable consumption (FL) CI low
v076_cihigh	Fruit and vegetable consumption (FL) CI high
v078_rawvalue	Adults engaging in moderate physical activity (F raw value
v078_numerator	Adults engaging in moderate physical activity (F numerator
v078_denominator	Adults engaging in moderate physical activity (F denominator
v078_cilow	Adults engaging in moderate physical activity (F CI low
v078_cihigh	Adults engaging in moderate physical activity (F CI high
v094_rawvalue	Obese adults (NY) raw value
v094_numerator	Obese adults (NY) numerator
v094_denominator	Obese adults (NY) denominator
v094_cilow	Obese adults (NY) CI low
v094_cihigh	Obese adults (NY) CI high
v093_rawvalue	No leisure time physical activity (NY) raw value
v093_numerator	No leisure time physical activity (NY) numerator
v093_denominator	No leisure time physical activity (NY) denominator
v093_cilow	No leisure time physical activity (NY) CI low
v093_cihigh	No leisure time physical activity (NY) CI high
v072_rawvalue	Excessive drinking (FL) raw value
v072_numerator	Excessive drinking (FL) numerator
v072_denominator	Excessive drinking (FL) denominator
v072_cilow	Excessive drinking (FL) CI low
v072_cihigh	Excessive drinking (FL) CI high
v089_rawvalue	Binge drinking (NY) raw value

Variable Name	Measure
v089_numerator	Binge drinking (NY) numerator
v089_denominator	Binge drinking (NY) denominator
v089_cilow	Binge drinking (NY) CI low
v089_cihigh	Binge drinking (NY) CI high
v092_rawvalue	Excessive drinking (NY) raw value
v092_numerator	Excessive drinking (NY) numerator
v092_denominator	Excessive drinking (NY) denominator
v092_cilow	Excessive drinking (NY) CI low
v092_cihigh	Excessive drinking (NY) CI high
v018_rawvalue	Motor vehicle crash occupancy rate raw value
v018_numerator	Motor vehicle crash occupancy rate numerator
v018_denominator	Motor vehicle crash occupancy rate denominator
v018_cilow	Motor vehicle crash occupancy rate CI low
v018_cihigh	Motor vehicle crash occupancy rate CI high
v019_rawvalue	On-road motor vehicle crash-related ER visits raw value
v019_numerator	On-road motor vehicle crash-related ER visits numerator
v019_denominator	On-road motor vehicle crash-related ER visits denominator
v019_cilow	On-road motor vehicle crash-related ER visits CI low
v019_cihigh	On-road motor vehicle crash-related ER visits CI high
v020_rawvalue	Off-road motor vehicle crash-related ER visits raw value
v020_numerator	Off-road motor vehicle crash-related ER visits numerator
v020_denominator	Off-road motor vehicle crash-related ER visits denominator
v020_cilow	Off-road motor vehicle crash-related ER visits CI low
v020_cihigh	Off-road motor vehicle crash-related ER visits CI high
v006_rawvalue	No recent dental visit raw value
v006_numerator	No recent dental visit numerator
v006_denominator	No recent dental visit denominator
v006_cilow	No recent dental visit CI low
v006_cihigh	No recent dental visit CI high
v047_rawvalue	Did not get needed health care raw value
v047_numerator	Did not get needed health care numerator

Variable Name	Measure
v047_denominator	Did not get needed health care denominator
v047_cilow	Did not get needed health care CI low
v047_cihigh	Did not get needed health care CI high
v077_rawvalue	Adults who have a personal doctor (FL) raw value
v077_numerator	Adults who have a personal doctor (FL) numerator
v077_denominator	Adults who have a personal doctor (FL) denominator
v077_cilow	Adults who have a personal doctor (FL) CI low
v077_cihigh	Adults who have a personal doctor (FL) CI high
v079_rawvalue	Insured adults (FL) raw value
v079_numerator	Insured adults (FL) numerator
v079_denominator	Insured adults (FL) denominator
v079_cilow	Insured adults (FL) CI low
v079_cihigh	Insured adults (FL) CI high
v090_rawvalue	Dental visit within the past year (NY) raw value
v090_numerator	Dental visit within the past year (NY) numerator
v090_denominator	Dental visit within the past year (NY) denominator
v090_cilow	Dental visit within the past year (NY) CI low
v090_cihigh	Dental visit within the past year (NY) CI high
v073_rawvalue	Adequate social/emotional support (FL) raw value
v073_numerator	Adequate social/emotional support (FL) numerator
v073_denominator	Adequate social/emotional support (FL) denominator
v073_cilow	Adequate social/emotional support (FL) CI low
v073_cihigh	Adequate social/emotional support (FL) CI high
v034_rawvalue	Lead poisoned children (WI) raw value
v034_numerator	Lead poisoned children (WI) numerator
v034_denominator	Lead poisoned children (WI) denominator
v034_cilow	Lead poisoned children (WI) CI low
v034_cihigh	Lead poisoned children (WI) CI high
v033_rawvalue	Municipal water (WI) raw value
v033_numerator	Municipal water (WI) numerator
v033_denominator	Municipal water (WI) denominator

Variable Name	Measure
v033_cilow	Municipal water (WI) CI low
v033_cihigh	Municipal water (WI) CI high
v048_rawvalue	Contaminants in municipal water (WI) raw value
v048_numerator	Contaminants in municipal water (WI) numerator
v048_denominator	Contaminants in municipal water (WI) denominator
v048_cilow	Contaminants in municipal water (WI) CI low
v048_cihigh	Contaminants in municipal water (WI) CI high
