# Supplementary material for: The financial consequences of undiagnosed memory disorders
Source: J financ econ. Author manuscript; Available in PMC 2025 Dec 16. (PMC12705035; doi:10.1016/j.jfineco.2025.104149)
Supplement: Data Replication Files [file NIHMS2128506-supplement-Data_Replication_Files.zip › replication_final/data_raw/geo/health_rank/DataDictionary_2017.pdf]

County Health Rankings 2017 Data Dictionary

Variable Name	Measure
statecode	State FIPS Code
countycode	County FIPS Code
fipscode	5-digit FIPS Code
state	State Abbreviation
county	Name
year	Release Year
county_ranked	County Ranked (Yes=1/No=0)
v001_rawvalue	Premature death raw value
v001_numerator	Premature death numerator
v001_denominator	Premature death denominator
v001_cilow	Premature death CI low
v001_cihigh	Premature death CI high
v002_rawvalue	Poor or fair health raw value
v002_numerator	Poor or fair health numerator
v002_denominator	Poor or fair health denominator
v002_cilow	Poor or fair health CI low
v002_cihigh	Poor or fair health CI high
v036_rawvalue	Poor physical health days raw value
v036_numerator	Poor physical health days numerator
v036_denominator	Poor physical health days denominator
v036_cilow	Poor physical health days CI low
v036_cihigh	Poor physical health days CI high
v042_rawvalue	Poor mental health days raw value
v042_numerator	Poor mental health days numerator
v042_denominator	Poor mental health days denominator
v042_cilow	Poor mental health days CI low
v042_cihigh	Poor mental health days CI high
v037_rawvalue	Low birthweight raw value
v037_numerator	Low birthweight numerator
v037_denominator	Low birthweight denominator
v037_cilow	Low birthweight CI low

Variable Name	Measure
v037_cihigh	Low birthweight CI high
v009_rawvalue	Adult smoking raw value
v009_numerator	Adult smoking numerator
v009_denominator	Adult smoking denominator
v009_cilow	Adult smoking CI low
v009_cihigh	Adult smoking CI high
v011_rawvalue	Adult obesity raw value
v011_numerator	Adult obesity numerator
v011_denominator	Adult obesity denominator
v011_cilow	Adult obesity CI low
v011_cihigh	Adult obesity CI high
v133_rawvalue	Food environment index raw value
v133_numerator	Food environment index numerator
v133_denominator	Food environment index denominator
v133_cilow	Food environment index CI low
v133_cihigh	Food environment index CI high
v070_rawvalue	Physical inactivity raw value
v070_numerator	Physical inactivity numerator
v070_denominator	Physical inactivity denominator
v070_cilow	Physical inactivity CI low
v070_cihigh	Physical inactivity CI high
v132_rawvalue	Access to exercise opportunities raw value
v132_numerator	Access to exercise opportunities numerator
v132_denominator	Access to exercise opportunities denominator
v132_cilow	Access to exercise opportunities CI low
v132_cihigh	Access to exercise opportunities CI high
v049_rawvalue	Excessive drinking raw value
v049_numerator	Excessive drinking numerator
v049_denominator	Excessive drinking denominator
v049_cilow	Excessive drinking CI low
v049_cihigh	Excessive drinking CI high

Variable Name	Measure
v134_rawvalue	Alcohol-impaired driving deaths raw value
v134_numerator	Alcohol-impaired driving deaths numerator
v134_denominator	Alcohol-impaired driving deaths denominator
v134_cilow	Alcohol-impaired driving deaths CI low
v134_cihigh	Alcohol-impaired driving deaths CI high
v045_rawvalue	Sexually transmitted infections raw value
v045_numerator	Sexually transmitted infections numerator
v045_denominator	Sexually transmitted infections denominator
v045_cilow	Sexually transmitted infections CI low
v045_cihigh	Sexually transmitted infections CI high
v014_rawvalue	Teen births raw value
v014_numerator	Teen births numerator
v014_denominator	Teen births denominator
v014_cilow	Teen births CI low
v014_cihigh	Teen births CI high
v085_rawvalue	Uninsured raw value
v085_numerator	Uninsured numerator
v085_denominator	Uninsured denominator
v085_cilow	Uninsured CI low
v085_cihigh	Uninsured CI high
v004_rawvalue	Primary care physicians raw value
v004_numerator	Primary care physicians numerator
v004_denominator	Primary care physicians denominator
v004_cilow	Primary care physicians CI low
v004_cihigh	Primary care physicians CI high
v004_other_data_1	Ratio of population to primary care physicians
v088_rawvalue	Dentists raw value
v088_numerator	Dentists numerator
v088_denominator	Dentists denominator
v088_cilow	Dentists CI low
v088_cihigh	Dentists CI high

Measures are listed in order of appearance on the county snapshots and analytic datasets.
Measures with the description "CI low" or "CI high" indicate the lower and upper limits of the confidence interval.

Variable Name	Measure
v088_other_data_1	Ratio of population to dentists
v062_rawvalue	Mental health providers raw value
v062_numerator	Mental health providers numerator
v062_denominator	Mental health providers denominator
v062_cilow	Mental health providers CI low
v062_cihigh	Mental health providers CI high
v062_other_data_1	Ratio of population to mental health providers
v005_rawvalue	Preventable hospital stays raw value
v005_numerator	Preventable hospital stays numerator
v005_denominator	Preventable hospital stays denominator
v005_cilow	Preventable hospital stays CI low
v005_cihigh	Preventable hospital stays CI high
v007_rawvalue	Diabetes monitoring raw value
v007_numerator	Diabetes monitoring numerator
v007_denominator	Diabetes monitoring denominator
v007_cilow	Diabetes monitoring CI low
v007_cihigh	Diabetes monitoring CI high
v050_rawvalue	Mammography screening raw value
v050_numerator	Mammography screening numerator
v050_denominator	Mammography screening denominator
v050_cilow	Mammography screening CI low
v050_cihigh	Mammography screening CI high
v021_rawvalue	High school graduation raw value
v021_numerator	High school graduation numerator
v021_denominator	High school graduation denominator
v021_cilow	High school graduation CI low
v021_cihigh	High school graduation CI high
v069_rawvalue	Some college raw value
v069_numerator	Some college numerator
v069_denominator	Some college denominator
v069_cilow	Some college CI low

Variable Name	Measure
v069_cihigh	Some college CI high
v023_rawvalue	Unemployment raw value
v023_numerator	Unemployment numerator
v023_denominator	Unemployment denominator
v023_cilow	Unemployment CI low
v023_cihigh	Unemployment CI high
v024_rawvalue	Children in poverty raw value
v024_numerator	Children in poverty numerator
v024_denominator	Children in poverty denominator
v024_cilow	Children in poverty CI low
v024_cihigh	Children in poverty CI high
v044_rawvalue	Income inequality raw value
v044_numerator	Income inequality numerator
v044_denominator	Income inequality denominator
v044_cilow	Income inequality CI low
v044_cihigh	Income inequality CI high
v082_rawvalue	Children in single-parent households raw value
v082_numerator	Children in single-parent households numerator
v082_denominator	Children in single-parent households denominator
v082_cilow	Children in single-parent households CI low
v082_cihigh	Children in single-parent households CI high
v140_rawvalue	Social associations raw value
v140_numerator	Social associations numerator
v140_denominator	Social associations denominator
v140_cilow	Social associations CI low
v140_cihigh	Social associations CI high
v043_rawvalue	Violent crime raw value
v043_numerator	Violent crime numerator
v043_denominator	Violent crime denominator
v043_cilow	Violent crime CI low
v043_cihigh	Violent crime CI high

Variable Name	Measure
v135_rawvalue	Injury deaths raw value
v135_numerator	Injury deaths numerator
v135_denominator	Injury deaths denominator
v135_cilow	Injury deaths CI low
v135_cihigh	Injury deaths CI high
v125_rawvalue	Air pollution - particulate matter raw value
v125_numerator	Air pollution - particulate matter numerator
v125_denominator	Air pollution - particulate matter denominator
v125_cilow	Air pollution - particulate matter CI low
v125_cihigh	Air pollution - particulate matter CI high
v124_rawvalue	Drinking water violations raw value
v124_numerator	Drinking water violations numerator
v124_denominator	Drinking water violations denominator
v124_cilow	Drinking water violations CI low
v124_cihigh	Drinking water violations CI high
v136_rawvalue	Severe housing problems raw value
v136_numerator	Severe housing problems numerator
v136_denominator	Severe housing problems denominator
v136_cilow	Severe housing problems CI low
v136_cihigh	Severe housing problems CI high
v067_rawvalue	Driving alone to work raw value
v067_numerator	Driving alone to work numerator
v067_denominator	Driving alone to work denominator
v067_cilow	Driving alone to work CI low
v067_cihigh	Driving alone to work CI high
v137_rawvalue	Long commute - driving alone raw value
v137_numerator	Long commute - driving alone numerator
v137_denominator	Long commute - driving alone denominator
v137_cilow	Long commute - driving alone CI low
v137_cihigh	Long commute - driving alone CI high
v127_rawvalue	Premature age-adjusted mortality raw value

Variable Name	Measure
v127_numerator	Premature age-adjusted mortality numerator
v127_denominator	Premature age-adjusted mortality denominator
v127_cilow	Premature age-adjusted mortality CI low
v127_cihigh	Premature age-adjusted mortality CI high
v128_rawvalue	Child mortality raw value
v128_numerator	Child mortality numerator
v128_denominator	Child mortality denominator
v128_cilow	Child mortality CI low
v128_cihigh	Child mortality CI high
v129_rawvalue	Infant mortality raw value
v129_numerator	Infant mortality numerator
v129_denominator	Infant mortality denominator
v129_cilow	Infant mortality CI low
v129_cihigh	Infant mortality CI high
v144_rawvalue	Frequent physical distress raw value
v144_numerator	Frequent physical distress numerator
v144_denominator	Frequent physical distress denominator
v144_cilow	Frequent physical distress CI low
v144_cihigh	Frequent physical distress CI high
v145_rawvalue	Frequent mental distress raw value
v145_numerator	Frequent mental distress numerator
v145_denominator	Frequent mental distress denominator
v145_cilow	Frequent mental distress CI low
v145_cihigh	Frequent mental distress CI high
v060_rawvalue	Diabetes prevalence raw value
v060_numerator	Diabetes prevalence numerator
v060_denominator	Diabetes prevalence denominator
v060_cilow	Diabetes prevalence CI low
v060_cihigh	Diabetes prevalence CI high
v061_rawvalue	HIV prevalence raw value
v061_numerator	HIV prevalence numerator

Variable Name	Measure
v061_denominator	HIV prevalence denominator
v061_cilow	HIV prevalence CI low
v061_cihigh	HIV prevalence CI high
v139_rawvalue	Food insecurity raw value
v139_numerator	Food insecurity numerator
v139_denominator	Food insecurity denominator
v139_cilow	Food insecurity CI low
v139_cihigh	Food insecurity CI high
v083_rawvalue	Limited access to healthy foods raw value
v083_numerator	Limited access to healthy foods numerator
v083_denominator	Limited access to healthy foods denominator
v083_cilow	Limited access to healthy foods CI low
v083_cihigh	Limited access to healthy foods CI high
v138_rawvalue	Drug overdose deaths raw value
v138_numerator	Drug overdose deaths numerator
v138_denominator	Drug overdose deaths denominator
v138_cilow	Drug overdose deaths CI low
v138_cihigh	Drug overdose deaths CI high
v039_rawvalue	Motor vehicle crash deaths raw value
v039_numerator	Motor vehicle crash deaths numerator
v039_denominator	Motor vehicle crash deaths denominator
v039_cilow	Motor vehicle crash deaths CI low
v039_cihigh	Motor vehicle crash deaths CI high
v143_rawvalue	Insufficient sleep raw value
v143_numerator	Insufficient sleep numerator
v143_denominator	Insufficient sleep denominator
v143_cilow	Insufficient sleep CI low
v143_cihigh	Insufficient sleep CI high
v003_rawvalue	Uninsured adults raw value
v003_numerator	Uninsured adults numerator
v003_denominator	Uninsured adults denominator

Variable Name	Measure
v003_cilow	Uninsured adults CI low
v003_cihigh	Uninsured adults CI high
v122_rawvalue	Uninsured children raw value
v122_numerator	Uninsured children numerator
v122_denominator	Uninsured children denominator
v122_cilow	Uninsured children CI low
v122_cihigh	Uninsured children CI high
v086_rawvalue	Health care costs raw value
v086_numerator	Health care costs numerator
v086_denominator	Health care costs denominator
v086_cilow	Health care costs CI low
v086_cihigh	Health care costs CI high
v131_rawvalue	Other primary care providers raw value
v131_numerator	Other primary care providers numerator
v131_denominator	Other primary care providers denominator
v131_cilow	Other primary care providers CI low
v131_cihigh	Other primary care providers CI high
v131_other_data_1	Ratio of population to primary care providers other than physicians
v149_rawvalue	Disconnected youth raw value
v149_numerator	Disconnected youth numerator
v149_denominator	Disconnected youth denominator
v149_cilow	Disconnected youth CI low
v149_cihigh	Disconnected youth CI high
v063_rawvalue	Median household income raw value
v063_numerator	Median household income numerator
v063_denominator	Median household income denominator
v063_cilow	Median household income CI low
v063_cihigh	Median household income CI high
v065_rawvalue	Children eligible for free or reduced price lunch raw value
v065_numerator	Children eligible for free or reduced price lunch numerator
v065_denominator	Children eligible for free or reduced price lunch denominator

Variable Name	Measure
v065_cilow	Children eligible for free or reduced price lunch CI low
v065_cihigh	Children eligible for free or reduced price lunch CI high
v141_rawvalue	Residential segregation - black/white raw value
v141_numerator	Residential segregation - black/white numerator
v141_denominator	Residential segregation - black/white denominator
v141_cilow	Residential segregation - black/white CI low
v141_cihigh	Residential segregation - black/white CI high
v142_rawvalue	Residential segregation - non-white/white raw value
v142_numerator	Residential segregation - non-white/white numerator
v142_denominator	Residential segregation - non-white/white denominator
v142_cilow	Residential segregation - non-white/white CI low
v142_cihigh	Residential segregation - non-white/white CI high
v015_rawvalue	Homicides raw value
v015_numerator	Homicides numerator
v015_denominator	Homicides denominator
v015_cilow	Homicides CI low
v015_cihigh	Homicides CI high
v148_rawvalue	Firearm fatalities raw value
v148_numerator	Firearm fatalities numerator
v148_denominator	Firearm fatalities denominator
v148_cilow	Firearm fatalities CI low
v148_cihigh	Firearm fatalities CI high
v051_rawvalue	Population raw value
v051_numerator	Population numerator
v051_denominator	Population denominator
v051_cilow	Population CI low
v051_cihigh	Population CI high
v052_rawvalue	% below 18 years of age raw value
v052_numerator	% below 18 years of age numerator
v052_denominator	% below 18 years of age denominator
v052_cilow	% below 18 years of age CI low

Variable Name	Measure
v052_cihigh	% below 18 years of age CI high
v053_rawvalue	% 65 and older raw value
v053_numerator	% 65 and older numerator
v053_denominator	% 65 and older denominator
v053_cilow	% 65 and older CI low
v053_cihigh	% 65 and older CI high
v054_rawvalue	% Non-Hispanic African American raw value
v054_numerator	% Non-Hispanic African American numerator
v054_denominator	% Non-Hispanic African American denominator
v054_cilow	% Non-Hispanic African American CI low
v054_cihigh	% Non-Hispanic African American CI high
v055_rawvalue	% American Indian and Alaskan Native raw value
v055_numerator	% American Indian and Alaskan Native numerator
v055_denominator	% American Indian and Alaskan Native denominator
v055_cilow	% American Indian and Alaskan Native CI low
v055_cihigh	% American Indian and Alaskan Native CI high
v081_rawvalue	% Asian raw value
v081_numerator	% Asian numerator
v081_denominator	% Asian denominator
v081_cilow	% Asian CI low
v081_cihigh	% Asian CI high
v080_rawvalue	% Native Hawaiian/Other Pacific Islander raw value
v080_numerator	% Native Hawaiian/Other Pacific Islander numerator
v080_denominator	% Native Hawaiian/Other Pacific Islander denominator
v080_cilow	% Native Hawaiian/Other Pacific Islander CI low
v080_cihigh	% Native Hawaiian/Other Pacific Islander CI high
v056_rawvalue	% Hispanic raw value
v056_numerator	% Hispanic numerator
v056_denominator	% Hispanic denominator
v056_cilow	% Hispanic CI low
v056_cihigh	% Hispanic CI high

Variable Name	Measure
v126_rawvalue	% Non-Hispanic white raw value
v126_numerator	% Non-Hispanic white numerator
v126_denominator	% Non-Hispanic white denominator
v126_cilow	% Non-Hispanic white CI low
v126_cihigh	% Non-Hispanic white CI high
v059_rawvalue	% not proficient in English raw value
v059_numerator	% not proficient in English numerator
v059_denominator	% not proficient in English denominator
v059_cilow	% not proficient in English CI low
v059_cihigh	% not proficient in English CI high
v057_rawvalue	% Females raw value
v057_numerator	% Females numerator
v057_denominator	% Females denominator
v057_cilow	% Females CI low
v057_cihigh	% Females CI high
v058_rawvalue	% Rural raw value
v058_numerator	% Rural numerator
v058_denominator	% Rural denominator
v058_cilow	% Rural CI low
v058_cihigh	% Rural CI high
v104_rawvalue	Communicable disease raw value
v104_numerator	Communicable disease numerator
v104_denominator	Communicable disease denominator
v104_cilow	Communicable disease CI low
v104_cihigh	Communicable disease CI high
v100_rawvalue	Coronary heart disease hospitalizations raw value
v100_numerator	Coronary heart disease hospitalizations numerator
v100_denominator	Coronary heart disease hospitalizations denominator
v100_cilow	Coronary heart disease hospitalizations CI low
v100_cihigh	Coronary heart disease hospitalizations CI high
v101_rawvalue	Cerebrovascular disease hospitalizations raw value

Variable Name	Measure
v101_numerator	Cerebrovascular disease hospitalizations numerator
v101_denominator	Cerebrovascular disease hospitalizations denominator
v101_cilow	Cerebrovascular disease hospitalizations CI low
v101_cihigh	Cerebrovascular disease hospitalizations CI high
v105_rawvalue	Self-inflicted injury hospitalizations raw value
v105_numerator	Self-inflicted injury hospitalizations numerator
v105_denominator	Self-inflicted injury hospitalizations denominator
v105_cilow	Self-inflicted injury hospitalizations CI low
v105_cihigh	Self-inflicted injury hospitalizations CI high
v099_rawvalue	Cancer incidence raw value
v099_numerator	Cancer incidence numerator
v099_denominator	Cancer incidence denominator
v099_cilow	Cancer incidence CI low
v099_cihigh	Cancer incidence CI high
v010_rawvalue	Smoking during pregnancy raw value
v010_numerator	Smoking during pregnancy numerator
v010_denominator	Smoking during pregnancy denominator
v010_cilow	Smoking during pregnancy CI low
v010_cihigh	Smoking during pregnancy CI high
v108_rawvalue	Drug arrests raw value
v108_numerator	Drug arrests numerator
v108_denominator	Drug arrests denominator
v108_cilow	Drug arrests CI low
v108_cihigh	Drug arrests CI high
v109_rawvalue	Alcohol-related hospitalizations raw value
v109_numerator	Alcohol-related hospitalizations numerator
v109_denominator	Alcohol-related hospitalizations denominator
v109_cilow	Alcohol-related hospitalizations CI low
v109_cihigh	Alcohol-related hospitalizations CI high
v018_rawvalue	Motor vehicle crash occupancy rate raw value
v018_numerator	Motor vehicle crash occupancy rate numerator

Variable Name	Measure
v018_denominator	Motor vehicle crash occupancy rate denominator
v018_cilow	Motor vehicle crash occupancy rate CI low
v018_cihigh	Motor vehicle crash occupancy rate CI high
v019_rawvalue	On-road motor vehicle crash-related ER visits raw value
v019_numerator	On-road motor vehicle crash-related ER visits numerator
v019_denominator	On-road motor vehicle crash-related ER visits denominator
v019_cilow	On-road motor vehicle crash-related ER visits CI low
v019_cihigh	On-road motor vehicle crash-related ER visits CI high
v020_rawvalue	Off-road motor vehicle crash-related ER visits raw value
v020_numerator	Off-road motor vehicle crash-related ER visits numerator
v020_denominator	Off-road motor vehicle crash-related ER visits denominator
v020_cilow	Off-road motor vehicle crash-related ER visits CI low
v020_cihigh	Off-road motor vehicle crash-related ER visits CI high
v006_rawvalue	No recent dental visit raw value
v006_numerator	No recent dental visit numerator
v006_denominator	No recent dental visit denominator
v006_cilow	No recent dental visit CI low
v006_cihigh	No recent dental visit CI high
v047_rawvalue	Did not get needed health care raw value
v047_numerator	Did not get needed health care numerator
v047_denominator	Did not get needed health care denominator
v047_cilow	Did not get needed health care CI low
v047_cihigh	Did not get needed health care CI high
v103_rawvalue	Childhood immunizations raw value
v103_numerator	Childhood immunizations numerator
v103_denominator	Childhood immunizations denominator
v103_cilow	Childhood immunizations CI low
v103_cihigh	Childhood immunizations CI high
v112_rawvalue	Local health department staffing raw value
v112_numerator	Local health department staffing numerator
v112_denominator	Local health department staffing denominator

Variable Name	Measure
v112_cilow	Local health department staffing CI low
v112_cihigh	Local health department staffing CI high
v116_rawvalue	Reading proficiency raw value
v116_numerator	Reading proficiency numerator
v116_denominator	Reading proficiency denominator
v116_cilow	Reading proficiency CI low
v116_cihigh	Reading proficiency CI high
v117_rawvalue	W-2 enrollment raw value
v117_numerator	W-2 enrollment numerator
v117_denominator	W-2 enrollment denominator
v117_cilow	W-2 enrollment CI low
v117_cihigh	W-2 enrollment CI high
v118_rawvalue	Poverty raw value
v118_numerator	Poverty numerator
v118_denominator	Poverty denominator
v118_cilow	Poverty CI low
v118_cihigh	Poverty CI high
v120_rawvalue	Older adults living alone raw value
v120_numerator	Older adults living alone numerator
v120_denominator	Older adults living alone denominator
v120_cilow	Older adults living alone CI low
v120_cihigh	Older adults living alone CI high
v121_rawvalue	Hate crimes raw value
v121_numerator	Hate crimes numerator
v121_denominator	Hate crimes denominator
v121_cilow	Hate crimes CI low
v121_cihigh	Hate crimes CI high
v119_rawvalue	Child abuse raw value
v119_numerator	Child abuse numerator
v119_denominator	Child abuse denominator
v119_cilow	Child abuse CI low

Variable Name	Measure
v119_cihigh	Child abuse CI high
v106_rawvalue	Injury hospitalizations raw value
v106_numerator	Injury hospitalizations numerator
v106_denominator	Injury hospitalizations denominator
v106_cilow	Injury hospitalizations CI low
v106_cihigh	Injury hospitalizations CI high
v107_rawvalue	Fall fatalities 65+ raw value
v107_numerator	Fall fatalities 65+ numerator
v107_denominator	Fall fatalities 65+ denominator
v107_cilow	Fall fatalities 65+ CI low
v107_cihigh	Fall fatalities 65+ CI high
v123_rawvalue	Year structure built raw value
v123_numerator	Year structure built numerator
v123_denominator	Year structure built denominator
v123_cilow	Year structure built CI low
v123_cihigh	Year structure built CI high
v008_rawvalue	Male population 0-17 raw value
v013_rawvalue	Male population 18-44 raw value
v016_rawvalue	Male population 45-64 raw value
v017_rawvalue	Male population 65+ raw value
v025_rawvalue	Total male population raw value
v026_rawvalue	Female population 0-17 raw value
v027_rawvalue	Female population 18-44 raw value
v031_rawvalue	Female population 45-64 raw value
v032_rawvalue	Female population 65+ raw value
v035_rawvalue	Total female population raw value
v097_rawvalue	Population growth raw value
