# Supplementary material for: The financial consequences of undiagnosed memory disorders
Source: J financ econ. Author manuscript; Available in PMC 2025 Dec 16. (PMC12705035; doi:10.1016/j.jfineco.2025.104149)
Supplement: Data Replication Files [file NIHMS2128506-supplement-Data_Replication_Files.zip › replication_final/README.docx]

Replication Package for “The Financial Consequences of Undiagnosed Memory Disorders”

Carole Roan Gresenz, Jean M. Mitchell, Belicia Rodriguez, Crystal Wang, R. Scott Turner, Wilbert van der Klaauw

**Overview**

The code in this replication package uses Stata to construct the tables and figures in the paper using pseudo credit and medical data as well as public American Community Survey and County Health Rankings data. One file runs three programs that clean data, run regression analyses, and create output. The replicator should expect the code to take 35.5 hours to run and require 11GB memory.

Note that the pseudo data does not follow the distribution of the original data sources, so the tables and figures produced do not resemble those in the paper. Moreover, the variable names in the pseudo data do not match those in the original data. Authorized users may request a crosswalk from Francis Mahoney ([francis.mahoney@ny.frb.org](mailto:francis.mahoney@ny.frb.org)).

**Data**

Our paper analyzes microdata from the Consumer Credit Panel (CCP) merged with Center for Medicare and Medicaid Services (CMS) Medicare administrative files using Social Security number (SSN).

**CMS Data:** The CMS data we requested and received are Medicare Master Beneficiary Summary File (MBSF) base, chronic conditions, and cost and utilization segments from 2000-2017 for a custom cohort. Researchers interested in accessing research identifiable files (RIFs) from the Center for Medicare and Medicaid Services (CMS) must consult the Research Data Assistance Center (ResDAC). More information on ResDAC and the application process for CMS data is available at [https://resdac.org/](https://gcc02.safelinks.protection.outlook.com/?url=https%3A%2F%2Fresdac.org%2F&data=05%7C02%7CCrystal.Wang%40ny.frb.org%7Cea54d7932ee44218cff508ddad24a8cb%7Cb397c6535b19463fb9fcaf658ded9128%7C1%7C0%7C638857095145874168%7CUnknown%7CTWFpbGZsb3d8eyJFbXB0eU1hcGkiOnRydWUsIlYiOiIwLjAuMDAwMCIsIlAiOiJXaW4zMiIsIkFOIjoiTWFpbCIsIldUIjoyfQ%3D%3D%7C0%7C%7C%7C&sdata=NnCmwRGaSDGaoVk3c%2FwyYiQcXFrsUlw0qmT0XRS%2FRY0%3D&reserved=0).

**CCP Data:** We used the 5% household sample of the CCP. Access to the CCP microdata is limited to Federal Reserve System researchers and their coauthors, due to contractual limitations with the data provider (Equifax). More information about the design and structure of the CCP is available at [https://fedinprint.org/item/fednsr/12522](https://gcc02.safelinks.protection.outlook.com/?url=https%3A%2F%2Ffedinprint.org%2Fitem%2Ffednsr%2F12522&data=05%7C02%7CCrystal.Wang%40ny.frb.org%7Cea54d7932ee44218cff508ddad24a8cb%7Cb397c6535b19463fb9fcaf658ded9128%7C1%7C0%7C638857095145896203%7CUnknown%7CTWFpbGZsb3d8eyJFbXB0eU1hcGkiOnRydWUsIlYiOiIwLjAuMDAwMCIsIlAiOiJXaW4zMiIsIkFOIjoiTWFpbCIsIldUIjoyfQ%3D%3D%7C0%7C%7C%7C&sdata=YUjimeAuTddj7qN6L6%2FrE1V1Jskx3Zle5hnoSGIaJsk%3D&reserved=0).

**Census Data:** American Community Survey data is publicly available at <https://data.census.gov>. Census crosswalk data is publicly available at <https://www.census.gov>.

**CHR:** County Health Rankings data is publicly available at <https://www.countyhealthrankings.org/>.

**Data Creation Process and Timeline:**

To create our data, a Federal Reserve Bank of New York (FRBNY) analyst identified primary members of the CCP who were born in 1954 or earlier and all their household members (regardless of birth year) on December 11, 2020. The FRBNY sent a list of the anonymous CCP IDs for these individuals to Equifax. Equifax took from this list the individuals who had an active or inactive credit record in December 2019, then developed a list of SSNs for these individuals. Equifax transmitted a file that included the SSNs and the anonymous CCP ID to CMS (through its contractor who handles CMS data processing, GDIT) on September 10, 2021.

CMS used the Equifax file as a “finder file” and identified individuals in the Medicare Master Beneficiary Summary File (MBSF) whose SSN matched that on the finder file. CMS sent a file with the anonymous CCP ID and the CMS data for the individuals in the finder file to FRBNY on November 9, 2021. FRBNY merged the Medicare data from CMS to the CCP data using the anonymous CCP ID on December 16, 2021.

Data for the 2005-2010 ACS was downloaded from Census table S1501 on March 16, 2022. The 2010 Census Tract Relationship data was downloaded on April 8, 2022. CHR data for 2011 and 2017 was downloaded from the County Health Rankings & Roadmap national data & documentation on August 30, 2024.

| File | Source | Notes |
| --- | --- | --- |
| data_raw/ccp/  ccp_raw.dta | Pseudo data. Original data from CCP |  |
| data_raw/cms/  unique_bene_xwalk.dta | Pseudo data. Original data from CMS |  |
| data_raw/cms/  adrd_episodes_fin.dta | Pseudo data. Original data from CMS | CMS sent flat files separately for the years 2000-2017 and SAS code to process them. This file has all the years appended |
| data_raw/cms/  cms_yearly_enrollment.dta | Pseudo data. Original data from CMS |  |
| data_raw/cms/  cms_cc_mid_eoy.dta | Pseudo data. Original data from CMS |  |
| data_raw/geo/census/  tractxw/us2010trf.txt | Census ([www2.census.gov/geo/docs/maps-data/data/rel/trf_txt/us2010trf.txt](https://www2.census.gov/geo/docs/maps-data/data/rel/trf_txt/us2010trf.txt)) | Data dictionary (us2010trf.dct) in same folder |
| data_raw/geo/census/  tractlevel/  ACSST5Y[year].S1501_data_with_overlays_2022-03-14T134847 | Census (<https://data.census.gov/table?q=S1501:%20Educational%20Attainment>) | [year] is 2000-2017 |
| data_raw/geo/  health_rank/  health_rank.dta | County Health Rankings & Roadmap (<https://www.countyhealthrankings.org/health-data/methodology-and-sources/data-documentation/national-data-documentation-2010-2023>), 2011 and 2017 | Data dictionaries in same folder |
| data_raw/Nicholas Table A3.xlsx | JAMA Internal Medicine (<https://jamanetwork.com/journals/jamainternalmedicine/fullarticle/2773241>), Appendix Table 3 in PDF supplement |  |

**Code**

**Software Requirements:** The code is run using Stata MP 16. The first section of the program “setup_cmsccp.do” installs dependencies.

**Memory, Runtime, Storage Requirements:** The code requires 11GB memory and 35.5 hours to run. The raw data and code require 7.1GB storage space. The intermediate data and final outputs require 57GB storage.

**Programs:**

- code/setup_cmsccp.do installs dependencies and defines globals and Stata programs necessary for all other do-files. The replicator must redefine the global “work” to that of their working directory.
- code/MAIN.do runs all relevant code- the setup, the data cleaning, the regression analyses, and the table and figure generation. The replicator may have to change the line that runs setup_cmsccp.do so that it points to the file in the correct directory.
  - code/1_process_data/PROCESSDATA.do runs all data cleaning and analysis sample creation code in order. It also creates as output the values in Figure 1. It takes 17 minutes to run.
    - code/1_process_data/cms_queries.sql is not to be run by the replicator but shows how FRBNY created the anonymous CCP ID and selected the CCP members for CMS matching.
  - code/2_run_regressions/REGRESSIONS.do runs all analysis code in order. It runs the propensity score matching that determines regression weights for each sample, then runs event studies under 23 specifications. It also creates as output Figure A.1 and the values in Tables A.2 and A.3. It takes 35 hours and 20 minutes to run.
  - code/3_create_output/CREATEOUTPUT.do runs code that creates the remaining tables and figures in the paper. Output is named intuitively (tab2.csv, figA7_ami_dqtot_flag.png,) and lies in the folders output/tables and output/figures. It takes less than one minute to run.
    - Note that in two of the sensitivity tests (figA3 and figA4), the results between the two specifications are so similar that only one set of coefficients appears visible.
    - Also note that Figure 1 and all the tables in the paper were manually converted from csv to png or LaTeX form. The code only generates the csv.

**List of Tables and Programs**

| Figure/Table # | Program | Line Number | Output File |
| --- | --- | --- | --- |
| Figure 1 | code/1_process_data/merge_all.do | 381 | fig1.csv |
| Figure 2 | code/3_create_output/Fig2_14.do | 15 | fig2_riskscore_f.png,  fig2_dqtot_flag.png |
| Figure 3 | code/3_create_output/Fig2_14.do | 28 | fig3_riskscore_f.png,  fig3_dqtot_flag.png |
| Figure 4 | code/3_create_output/Fig2_14.do | 41 | fig4_subprime_riskscore_f.png,  fig4_dqtot_flag.png |
| Figure 5 | code/3_create_output/Fig2_14.do | 50 | fig5_dqcc_flag_cond.png,  fig5_dqcc_bal_cond.png |
| Figure 6 | code/3_create_output/Fig2_14.do | 59 | fig6_util_cc_cond.png,  fig6_util_cc_cond_high.png |
| Figure 7 | code/3_create_output/Fig2_14.do | 68 | fig7_dqm_flag_cond.png,  fig7_dqm_bal_cond.png |
| Figure 8 | code/3_create_output/Fig2_14.do | 81 | fig8_riskscore_f.png,  fig8_dqtot_flag.png |
| Figure 9 | code/3_create_output/Fig2_14.do | 94 | fig9_riskscore_f.png,  fig9_dqtot_flag.png |
| Figure 10 | code/3_create_output/Fig2_14.do | 107 | fig10_riskscore_f.png,  fig10_dqtot_flag.png |
| Figure 11 | code/3_create_output/Fig2_14.do | 120 | fig11_riskscore_f.png,  fig11_dqtot_flag.png |
| Figure 12 | code/3_create_output/Fig2_14.do | 133 | fig12_riskscore_f.png,  fig12_dqtot_flag.png |
| Figure 13 | code/3_create_output/Fig2_14.do | 142 | fig13_riskscore_f.png,  fig13_dqtot_flag.png |
| Figure 14 | code/3_create_output/Fig2_14.do | 156 | fig14_cancer_riskscore_f.png,  fig14_hip_fracture_riskscore_f.png,  fig14_ra_oa_riskscore_f.png,  fig14_cancer_dqtot_flag.png,  fig14_hip_fracture_dqtot_flag.png,  fig14_ra_oa_dqtot_flag.png |
| Table 1 | code/3_create_output/tab1_2.do | 59 | tab1.csv |
| Table 2 | code/3_create_output/tab1_2.do | 116 | tab2.csv |
| Figure A1 | code/2_run_regressions/psm_main.do | 17 | figA1_unweighted.png, figA1_weighted.png |
| Figure A2 | code/3_create_output/figA2_A7.do | 17 | figA2_dqcc_flag_cond.png,  figA2_dqm_flag_cond.png,  figA2_util_cc_cond_high.png |
| Figure A3 | code/3_create_output/figA2_A7.do | 30 | figA3_riskscore_f.png,  figA3_dqtot_flag.png |
| Figure A4 | code/3_create_output/figA2_A7.do | 43 | figA4_riskscore_f.png,  figA4_dqtot_flag.png |
| Figure A5 | code/3_create_output/figA2_A7.do | 56 | figA5_riskscore_f.png,  figA5_dqtot_flag.png |
| Figure A6 | code/3_create_output/figA2_A7.do | 65 | figA6_dqcc_flag.png,  figA6_dqm_flag.png |
| Figure A7 | code/3_create_output/figA2_A7.do | 80, 84 | figA7_hyperl_riskscore_f.png,  figA7_depression_riskscore_f.png,  figA7_hypert_riskscore_f.png,  figA7_diabetes_riskscore_f.png,  figA7_ischemicheart_riskscore_f.png,  figA7_ami_riskscore_f.png,  figA7_glaucoma_riskscore_f.png,  figA7_combo, figA7_hyperl_dqtot_flag.png,  figA7_depression_ dqtot_flag.png,  figA7_hypert_ dqtot_flag.png,  figA7_diabetes_ dqtot_flag.png,  figA7_ischemicheart_ dqtot_flag.png,  figA7_ami_ dqtot_flag.png,  figA7_glaucoma_ dqtot_flag.png,  figA7_combo dqtot_flag.png |
| Table A1 | code/3_create_output/tabA1.do | 77 | tabA1.csv |
| Table A2 | code/2_run_regressions/reg_main.do | 39 | tabA2.csv |
| Table A3 | code/2_run_regressions/psm_main.do | 44 | tabA3.csv |
